# Global, regional, and national burden and projections to 2050 of occupational carcinogen-attributable nasopharyngeal and laryngeal cancer: a comprehensive analysis from the GBD 2021 study

**DOI:** 10.3389/fpubh.2025.1615378

**Published:** 2025-07-04

**Authors:** Hao Zhang, Rong Xue, Xinru Kong, Manyi Zhao, Zhanguo Jin

**Affiliations:** ^1^Department of Otorhinolaryngology Head and Neck Surgery, Air Force Medical Center, Air Force Medical University, Beijing, China; ^2^Department of Vertigo Center, Air Force Medical Center, Air Force Medical University, Beijing, China

**Keywords:** occupational carcinogens, nasopharyngeal cancer, laryngeal cancer, global burden of disease, trends, decomposition analysis

## Abstract

**Background:**

Occupational carcinogens contribute to the burden of nasopharyngeal (NPC) and laryngeal cancer (LC), yet their global impact and long-term trends remain insufficiently explored. This study estimates the burden, temporal trends, and future projections of occupational carcinogen-attributable NPC and LC from 1990 to 2050.

**Methods:**

Using data from Global Burden of Disease (GBD) 2021, we analyzed death and disability-adjusted life years (DALYs) across 204 countries and territories. Age-standardized death rate (ASDR), age-standardized DALYs rate, and estimated annual percentage change (EAPC) were used to assess trends. Decomposition analysis quantified the contributions of epidemiological changes, population growth, and aging. Future projections (2022–2050) were estimated using autoregressive integrated moving average (ARIMA) and exponential smoothing (ES) models.

**Results:**

Between 1990 and 2021, global deaths due to occupational carcinogen-attributable NPC increased by 25.4%, while DALYs rose by 17.0%, despite a 33.3% decline in the age-standardized rate (ASR) of DALYs. For LC, deaths cases increased by 29.4%, and DALYs rose by 22.0%, alongside a 42.9% reduction in ASDR and 42.7% in ASR of DALYs. Sex-specific trends revealed that males bore a disproportionately higher burden, particularly in high-risk occupational sectors. Across socio-demographic index (SDI) regions, the most significant increases in absolute burden were observed in low-middle and middle SDI regions, whereas high SDI regions exhibited the greatest declines in death and DALY rates. Decomposition analysis showed that population growth and aging were the primary drivers of increased burden in low- and middle-income regions, whereas epidemiological improvements helped offset burden in high-income regions. Projections to 2050 suggest that despite further reductions in age-standardized rates, the absolute number of deaths and DALYs will continue rising, particularly in regions experiencing rapid industrialization.

**Conclusion:**

The increasing absolute burden despite declining rates underscores the need for sustained occupational health interventions, particularly in low- and middle-income regions. Efforts such as expanding occupational health surveillance systems, tailoring region-specific exposure controls, and improving early-warning prediction tools will be essential to mitigating future occupational cancer risks.

## Introduction

Nasopharyngeal carcinoma (NPC) is an epithelial malignancy arising in the nasopharyngeal mucosa, characterized by a distinct geographic distribution and etiological profile. It is endemic in East and Southeast Asia, with particularly high incidence rates observed in countries such as China, Singapore, and Taiwan, while remaining rare in most Western populations. The development of NPC involves a multifactorial interplay of Epstein–Barr virus (EBV) infection, genetic susceptibility, dietary exposure to nitrosamine-rich preserved foods, and environmental and occupational carcinogens such as formaldehyde (1). Recent perspectives have reframed NPC as an ecological disease rather than a purely genetic malignancy, emphasizing the complex interactions between biological and environmental determinants. The “cancer ecology tree” has been proposed as a conceptual model to illustrate how viral, dietary, genetic, and occupational risk factors converge in the tumorigenic process (2).

Globally, nasopharyngeal and laryngeal cancers pose substantial public health burdens. According to GBD 2019, there were approximately 176,502 new cases and 71,610 deaths from NPC worldwide, leading to 2.34 million DALYs. Laryngeal cancer accounted for 209,149 new cases, 123,356 deaths, and 3.26 million DALYs. These burdens were most prominent in East and Southeast Asia (for NPC) and in Central and Eastern Europe and Latin America (for laryngeal cancer) (3).

Occupational exposure to carcinogens remains a critical yet underrecognized contributor to the global cancer burden, particularly for malignancies with complex etiological pathways such as nasopharyngeal (NPC) and laryngeal cancers(LC) (4, 5). Among these, formaldehyde has been identified as a relevant occupational carcinogen associated with elevated risk of NPC, particularly in high-exposure occupations such as furniture and textile manufacturing (6). While significant progress has been made in understanding occupational hazards, existing studies often lack granular analyses of demographic, regional, and socioeconomic disparities in occupational carcinogen-attributable nasopharyngeal and laryngeal cancer burden (7, 8). The interplay of population growth, aging, and epidemiological transitions remains poorly quantified, especially in low- and middle-income countries (LMICs) undergoing rapid industrialization (9).

This study addresses these gaps by comprehensively analyzing the global, regional, and national burden of occupational carcinogen-attributable NPC and LC from 1990 to 2021, employing decomposition methods to disentangle the contributions of demographic shifts and epidemiological changes. Our findings reveal that while age-standardized death and disability-adjusted life years (DALYs) rates declined globally for both cancers, absolute burdens increased substantially. Sex-specific disparities and socioeconomic stratification emerged as defining features of these malignancies, with males disproportionately affected. By projecting trends to 2050 and identifying region-specific vulnerabilities, this analysis provides evidence to guide targeted occupational health policies, emphasizing the urgent need to reconcile industrial growth with worker safety in an era of accelerating technological and demographic change.

## Methods

### Overview and methodological details

This study is a retrospective, population-based, secondary-data observational analysis utilizing publicly available data from the Global Burden of Disease (GBD) 2021 database to evaluate the burden of occupational carcinogen-attributable nasopharyngeal (NPC) and laryngeal cancer (LC) across 204 countries and territories from 1990 to 2021. The GBD study provides standardized estimates for death and disability-adjusted life years (DALYs) through a rigorous methodological framework, ensuring comparability over time and across geographic regions. In the GBD 2021 study, occupational carcinogen-attributable cancers were estimated using population-attributable fractions. Formaldehyde was the only occupational carcinogen attributed to nasopharyngeal cancer, while asbestos and sulfuric acid mists were attributed to laryngeal cancer. Occupational exposure was defined as long-term exposure in workplace settings exceeding background levels. Detailed descriptions of data sources, model development, and validation techniques used in GBD 2021 have been published elsewhere (10).

Key epidemiological indicators extracted for NPC and LC included annual deaths and DALYs, as well as age-standardized death rates (ASDR) and age-standardized DALYs rates (ASR of DALYs). To quantify temporal trends, the estimated annual percentage change (EAPC) was calculated using log-linear regression (11). The formula applied was:


EAPC=(e^β−1)×100

where *β* is the slope of the natural logarithm of the ASR over time, derived from the linear regression model:


ln(ASR)=β×Year+α

Here, α is the intercept. A positive EAPC indicates an increasing trend, while a negative EAPC suggests a decline. Statistical significance was defined as 95% CIs not crossing zero.

### Socio-demographic and regional analysis

To examine variations in disease burden by socioeconomic status, countries and regions were categorized according to the socio-demographic index (SDI), a composite measure incorporating fertility rates, educational attainment, and income per capita (12). This classification enabled an assessment of the relationship between socio-economic development and occupational carcinogen-related cancer burden. Additionally, regional comparisons were conducted across GBD-defined regions to evaluate geographical disparities in NPC and LC burden. Hierarchical clustering analysis was applied to identify regions exhibiting similar burden patterns.

### Decomposition analysis

To decompose the factors driving changes in death and DALYs, a decomposition analysis was performed to separate the contributions of epidemiological changes, population growth, and aging to the observed trends. This allowed for a clearer understanding of whether burden shifts were primarily driven by demographic expansion, workforce aging, or improvements in prevention and treatment (13).

### Predictive modeling

To forecast the future burden of NPC and LC from 2022 to 2050, we applied time-series modeling techniques based on historical ASR and number data. Specifically, we used autoregressive integrated moving average (ARIMA) models to capture short-term fluctuations, and exponential smoothing (ES) models for long-term trend estimation. Model selection was based on standard diagnostic criteria including AIC, BIC, residual autocorrelation checks, and visual inspection of fitted vs. actual plots. Goodness-of-fit was evaluated using mean absolute percentage error (MAPE) and root mean square error (RMSE). These models were implemented in accordance with established guidelines for time-series forecasting in global epidemiology. All projections are reported with 95% uncertainty intervals (UIs) derived from bootstrapped simulations to reflect estimation variability.

### Statistical analysis

All statistical analyses were performed using R software (version 4.4.2). Descriptive statistics for death and DALYs were presented as means with 95% UIs derived from 1,000 Monte Carlo simulations. Hierarchical clustering was performed using the *hclust* function in R, with Euclidean distance used to measure dissimilarity and Ward’s minimum variance method (*ward. D2*) applied to minimize intra-cluster variance. Log-linear regression was employed to compute EAPC, with statistical significance set at *p* < 0.05.

## Results

### Global burden of occupational carcinogen-attributable nasopharyngeal and laryngeal cancer in 2021

#### Nasopharyngeal cancer

**Figure 1:**
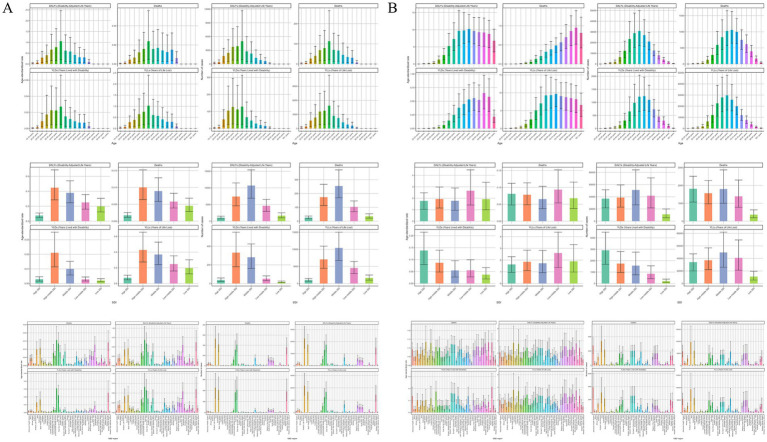
Numbers and age-standardized rates of occupational carcinogens-attributable deaths, DALYs, YLLs, and YLDs for **(A)** nasopharynx cancer and **(B)** larynx cancers by age group, SDI region, and GBD region in 2021. DALYs, disability-adjusted life years; YLLs, years of life lost; YLDs, years lived with disability; SDI, Socio-Demographic Index; GBD, Global Burden of Disease.

Occupational carcinogen-attributable NPC accounted for 592 deaths globally in 2021, with an ASR of 0.01 per 100,000. Males experienced a notably higher burden compared to females, comprising the majority of these deaths. Mortality increased progressively with age, peaking among those aged 45–49 years, and declining thereafter (Supplementary Table 1; Figure 1).

Across SDI regions, the absolute number of attributable deaths was highest in middle SDI regions, followed by high-middle and low-middle SDI regions; however, ASRs were generally consistent across these groups (0.01 per 100,000). At a regional level, Asia bore the heaviest burden, particularly East Asia, which reported the highest age-standardized death rate among all regions. Notably, many other regions, such as Australasia and North America, reported minimal or no deaths attributable to occupational carcinogens (Supplementary Table 1; Figure 1).

**Figure 2:**
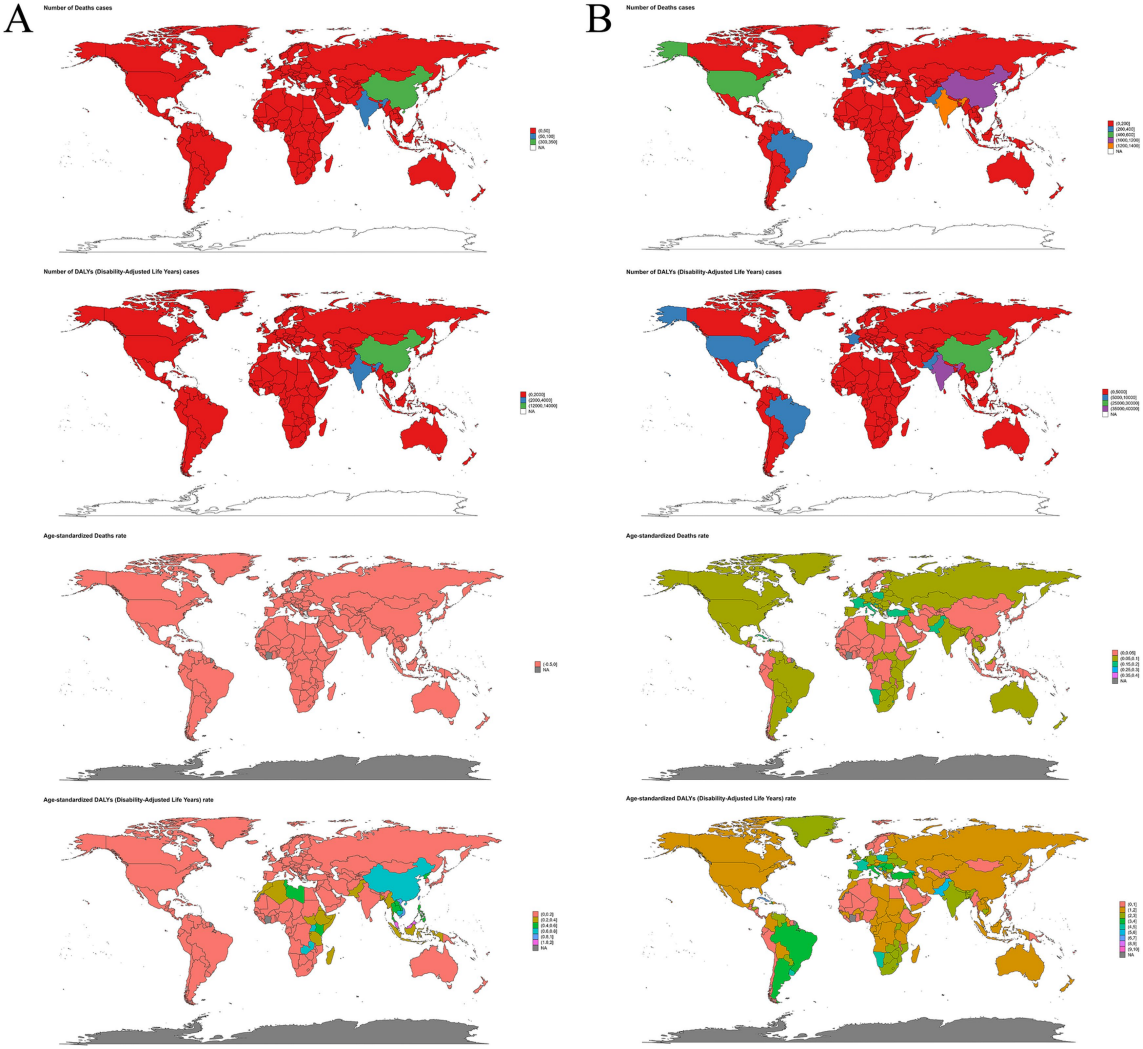
Numbers and age-standardized rates of occupational carcinogens-attributable deaths and DALYs for **(A)** nasopharynx cancer and **(B)** larynx cancer across countries and territories in 2021. DALYs, disability-adjusted life years.

China and India recorded the highest absolute numbers of attributable deaths globally in 2021, with China showing the second-highest age-standardized death rate among countries analyzed. However, Malaysia exhibited the highest country-specific age-standardized DALY rate, underscoring significant regional variations even within similar SDI contexts. Conversely, the majority of countries reported negligible or no attributable burden (Supplementary Table 2; Figure 2).

In terms of DALYs, occupational carcinogens accounted for 25,383 DALYs globally in 2021, corresponding to an age-standardized rate of 0.43 per 100,000. The DALYs burden was substantially higher among males compared to females. Age-wise, the greatest DALYs were observed in younger adults (ages 30–49), reflecting both greater workforce engagement and prolonged exposure to occupational carcinogens during working years (Supplementary Table 3; Figure 1).

The DALYs burden varied substantially across SDI regions, with the highest absolute DALYs occurring in middle and high-middle SDI regions. However, the highest age-standardized DALYs rates were seen in high-middle SDI countries, notably in East Asia, particularly China and Malaysia. In contrast, high-SDI regions, such as Australasia and North America, exhibited the lowest DALYs burdens, reflecting effective occupational health interventions and stringent regulatory environments. At the national level, China exhibited the highest age-standardized DALYs rates, followed by Malaysia and India, whereas numerous countries, including Montenegro, Norway, and Ghana, reported negligible attributable DALYs (Supplementary Tables 3, 4; Figures 1, 2).

#### Laryngeal cancer

In 2021, occupational carcinogens contributed to approximately 6,946 global deaths from laryngeal cancer, translating to an ASR of 0.08 per 100,000. The burden was notably higher among males compared to females. Deaths primarily occurred in older age groups, peaking among individuals aged 65–69 years, while younger individuals under 35 experienced negligible death (Supplementary Table 5; Figure 1).

When assessed by SDI, high and middle SDI regions experienced the greatest number of deaths. Regionally, Asia had the highest absolute death burden, followed by regions with advanced healthcare systems, such as Western Europe. Tropical Latin America and Western Europe had the highest ASRs, indicating significant ongoing occupational exposure risks despite advanced regulatory frameworks. Regions such as Oceania, High-income Asia Pacific, and Western Sub-Saharan Africa had substantially lower death rates (Supplementary Table 5; Figure 1).

At the national level, India and China recorded the highest numbers of occupational carcinogen-attributable deaths from laryngeal cancer. Monaco and Lesotho showed notably high ASRs, though their absolute death counts were minimal. Additionally, numerous countries reported no attributable deaths (Supplementary Table 6; Figure 2).

Globally, occupational carcinogens accounted for roughly 179,887 DALYs due to laryngeal cancer, with males bearing the vast majority of this burden compared to females. The highest DALYs occurred among adults aged 55–64 years, coinciding with prolonged occupational exposures (Table 1, Figure 1).

**Table 1:** Decomposition analysis of occupational carcinogen-attributable LC DALYs (1990–2021) sex and SDI disparities.

Location name	Increase	Aging (%)	Population growth (%)	Epidemiological change (%)
Global	4138.98	79.04	3930.76	−3909.8
Female	6395.11	17.86	167.92	−85.78
Male	−40283.07	43.8	−486.5	542.7
High-middle SDI	−48052.62	1.61	−45.07	143.46
Low-middle SDI	35820.92	30.68	83.03	−13.71
High SDI	−32604.1	1.65	−62.72	161.06
Low SDI	14273.83	13.29	119.17	−32.47
Middle SDI	21240.26	92.93	138.01	−130.94

By SDI classification, middle and high SDI regions faced the greatest DALY burdens, while low SDI regions had relatively lower burdens. Among GBD regions, Asia and regions characterized by basic healthcare systems had the highest absolute DALYs, whereas the Caribbean and Tropical Latin America reported the highest age-standardized DALY rates. Oceania and High-income Asia Pacific had the lowest burdens overall (Supplementary Table 7; Figure 1).

Country-specific analysis revealed that India and China had the highest absolute DALY burdens, while Monaco and Lesotho recorded the highest ASRs despite relatively few absolute DALYs. Several countries, including Samoa, Kiribati, and Guam, reported minimal or no attributable DALYs (Supplementary Table 8; Figure 2).

### Temporal trends in occupational carcinogen-attributable burden from 1990 to 2021

#### Nasopharyngeal cancer

Between 1990 and 2021, deaths due to occupational carcinogen-attributable nasopharyngeal cancer rose by 25.4%, while the ASDR remained unchanged. Similarly, the number of DALYs increased by 17.0%, though the ASR declined by 33.3% (Supplementary Tables 1, 3; Supplementary Figure 1).

Patterns by sex aligned with overall trends, with males experiencing higher absolute numbers of deaths and DALYs, while ASRs decreased in both sexes. Age-specific trends showed a consistent decline in ASRs, yet deaths and DALYs increased across most age groups, particularly among individuals aged 40–49, where the greatest fluctuations were observed (Supplementary Figure 2).

By SDI classification, ASRs for both deaths and DALYs declined across all levels, with the sharpest reductions recorded in high-middle and middle SDI regions. However, the absolute burden continued to grow, particularly in low-middle SDI regions, where deaths and DALYs more than doubled (Supplementary Figure 2).

**Figure 3:**
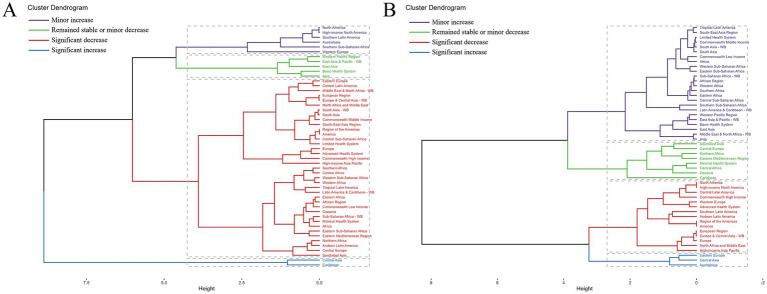
Results of cluster analysis based on the EAPC values of age-standardized death and DALYs rates attributable to occupational carcinogens for **(A)** nasopharynx cancer and **(B)** larynx cancer, 1990–2021. EAPC, estimated annual percentage change; DALYs, disability-adjusted life years.

Regional variations revealed distinct patterns in the occupational carcinogen-attributable burden of nasopharyngeal cancer. While most regions—including South Asia, Africa, Europe, the Americas, and Southeast Asia—exhibited declines, Central Asia and the Caribbean experienced substantial increases (Figure 3).

At the country level, Kenya and Pakistan recorded the largest increases in death, while Cameroon showed the greatest rise in DALYs, followed by Benin, Niger, and Togo. Conversely, significant reductions in burden were observed in multiple European nations, where some experienced a complete elimination of occupational carcinogen-attributable DALYs. The most pronounced declines in ASDR occurred in Ghana and Switzerland, while the highest increases in ASR were recorded in Cabo Verde and the Solomon Islands (Supplementary Tables 2, 4; Supplementary Figure 3).

#### Laryngeal cancer

Between 1990 and 2021, global deaths due to occupational carcinogen-attributable laryngeal cancer increased by 29.4%, while the ASDR declined by 42.9%. Similarly, DALYs increased by 22.0%, whereas the ASR of DALYs fell by 42.7%. These trends indicate a growing absolute burden despite overall progress in reducing death rates (Supplementary Tables 5, 7; Supplementary Figure 1).

Sex-specific patterns followed similar trajectories, with males experiencing greater absolute increases in deaths and DALYs, while females showed smaller but consistent changes. Across all age groups, ASRs declined, whereas the number of deaths and DALYs continued to rise, particularly among individuals aged 55–74 years. The most notable variations in ASRs were observed in the oldest age groups, whereas absolute death and DALY fluctuations were most pronounced in middle-aged and older populations (Supplementary Figure 2).

At the SDI level, ASRs for deaths and DALYs showed a downward trend across all regions, with the sharpest declines in high-middle and high SDI regions. However, absolute deaths and DALYs continued to rise in low, low-middle, and middle SDI regions, reflecting persistent occupational exposure risks in these settings (Supplementary Figure 2).

Geographically, regional disparities were evident. While declines were observed across North America, Central Latin America, Western Europe, and High-income Asia Pacific, increasing burdens were recorded in Eastern Europe, Central Asia, and Australasia (Figure 3).

National-level variations further illustrated this divergence. Kenya and Paraguay recorded the largest increases in death, while Honduras exhibited the greatest rise in DALYs. Monaco, Kazakhstan, and Estonia showed the most significant reductions. Changes in age-standardized rates followed similar patterns, with Kiribati and Greenland experiencing the highest increases, whereas Kazakhstan and Singapore demonstrated the most substantial declines (Tables 2, 3; Supplementary Figure 3).

**Table 2:** Decomposition analysis of occupational carcinogen-attributable NPC deaths (1990–2021) sex and SDI disparities.

Location name	Increase	Aging (%)	Population growth (%)	Epidemiological change (%)
Global	91.36	47.76	308.17	−255.93
Female	2.71	290.11	2837.16	−3027.27
Male	91.43	37.55	225.37	−162.92
High-middle SDI	−13.26	−114.67	−302.81	517.47
Low-middle SDI	55.09	33.58	65.43	1
High SDI	−5.29	0.32	−124.46	224.14
Low SDI	15.25	8.17	102.67	−10.85
Middle SDI	30.14	299.45	289.88	−489.32

**Table 3:** Decomposition analysis of occupational carcinogen-attributable NPC DALYs (1990–2021) sex and SDI disparities.

Location name	Increase	Aging (%)	Population growth (%)	Epidemiological change (%)
Global	−720.79	81.21	−1762.82	1781.6
Female	−1413.98	23.49	−246.95	323.46
Male	859.27	−21.1	1072.34	−951.24
High-middle SDI	−3408.9	69.09	−48.86	79.77
Low-middle SDI	2,907	3.72	96.1	0.18
High SDI	−460.25	53.02	−55.25	102.23
Low SDI	1337.47	10.73	99.74	−10.47
Middle SDI	−5549.01	33.89	−103.5	169.61

### Decomposition analysis of changes in occupational carcinogen-attributable burden

#### Nasopharyngeal cancer

**Figure 4:**
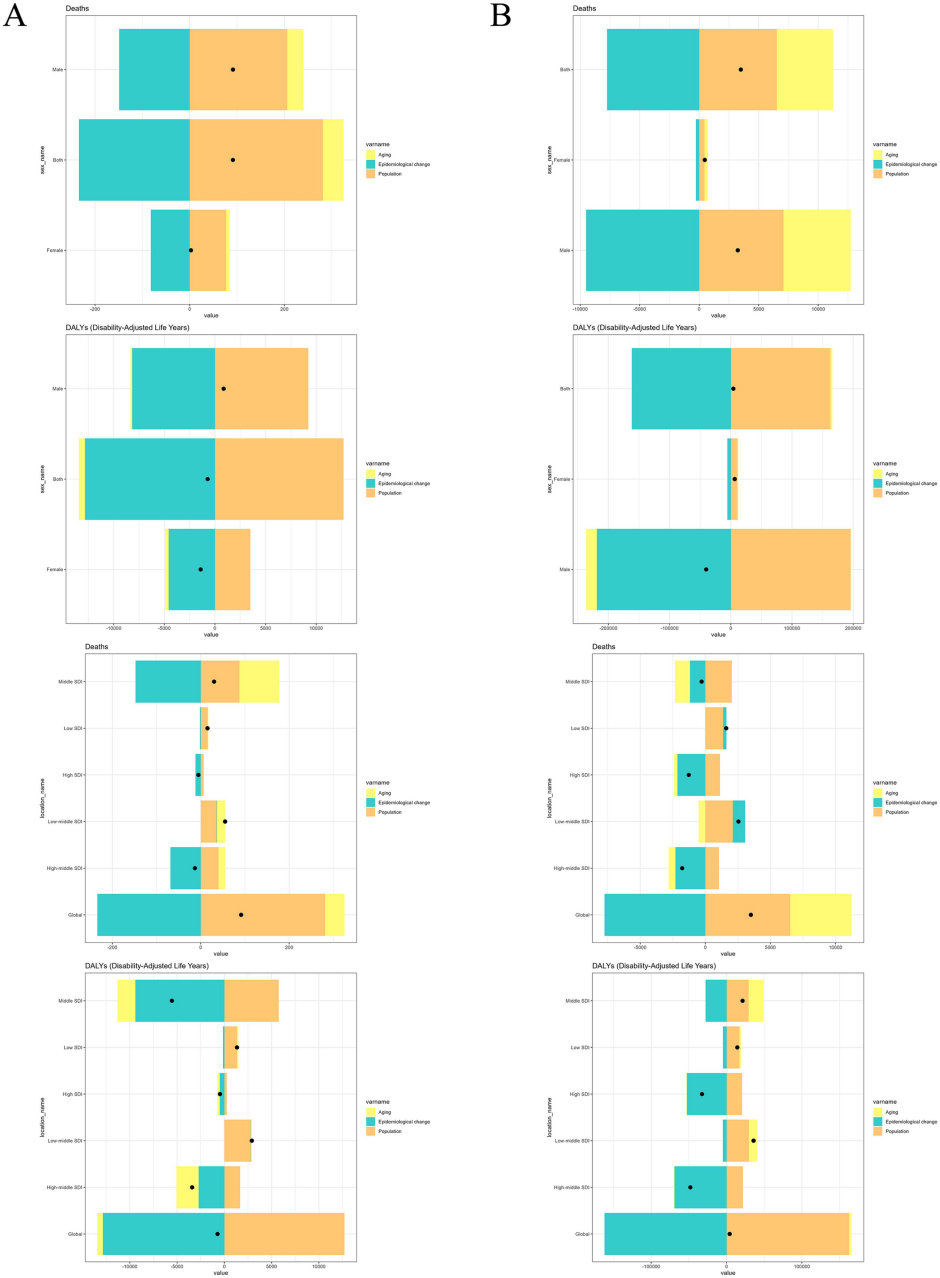
Decomposition analysis of changes in occupational carcinogens-attributable deaths and DALYs for **(A)** nasopharynx cancer and **(B)** larynx cancer from 1990 to 2021, stratified by sex and SDI regions. DALYs, disability-adjusted life years; SDI, Socio-Demographic Index.

From 1990 to 2021, population growth was the primary driver of the increase in occupational carcinogen-attributable deaths globally, followed by aging, while epidemiological improvements contributed to a reduction in death. The impact varied by sex, with population growth and aging contributing more substantially to female death, whereas epidemiological changes played a greater role in reducing deaths among males. Across SDI regions, epidemiological improvements led to death reductions in most high-income regions, whereas population growth and aging were the dominant contributors to increased deaths in low- and middle-SDI regions. The most pronounced death increase was observed in middle SDI regions, where population growth and aging had the strongest impact. For DALYs, epidemiological factors contributed the most to global changes, with aging playing a smaller but positive role, while population growth had a mixed impact across different regions. In high-middle and high SDI regions, epidemiological improvements helped offset increases in DALYs, whereas in low- and middle-SDI regions, population growth and aging continued to drive increases in burden (Tables 1, 4; Figure 4).

**Table 4:** Decomposition analysis of occupational carcinogen-attributable LC deaths (1990–2021) sex and SDI disparities.

Location name	Increase	Aging (%)	Population growth (%)	Epidemiological change (%)
Global	3499.65	134.69	186.46	−221.15
Female	465.37	62.8	95.13	−57.93
Male	3241.72	174.34	218.51	−292.85
High-middle SDI	−1779.13	29.37	−58.35	128.97
Low-middle SDI	2542.81	−20.12	83.23	36.9
High SDI	−1274.9	20.64	−88.04	167.39
Low SDI	1602.3	−0.91	85.45	15.47
Middle SDI	−287.32	399.1	−709.56	410.46

#### Laryngeal cancer

From 1990 to 2021, population growth and aging were the main contributors to the increase in occupational carcinogen-attributable laryngeal cancer deaths globally, while epidemiological improvements offset a portion of this increase. The impact varied by sex, with population growth and aging contributing more substantially to male death, whereas epidemiological changes played a greater role in reducing deaths among males compared to females. Across SDI regions, epidemiological improvements had a positive effect in high-middle and high SDI regions, while population growth and aging drove death increases in low- and middle-SDI regions. Middle SDI regions exhibited the strongest impact of aging and epidemiological changes, while population growth had a smaller influence in these settings. For DALYs, population growth was the most significant factor globally, followed by aging, whereas epidemiological changes contributed to a notable reduction. Sex-specific patterns showed that among females, population growth and aging played a greater role, while among males, epidemiological factors had a larger impact on DALY reductions. Regional differences revealed that epidemiological factors were the dominant contributors to DALY increases in high-middle and high SDI regions, while population growth and aging remained the primary drivers in low- and middle-SDI regions. In middle SDI regions, aging and population growth contributed to increased DALYs, whereas epidemiological improvements mitigated some of the burden (Tables 1, 4; Figure 4).

### Projections of future burden of occupational carcinogen-attributable nasopharyngeal and laryngeal cancer

**Figure 5:**
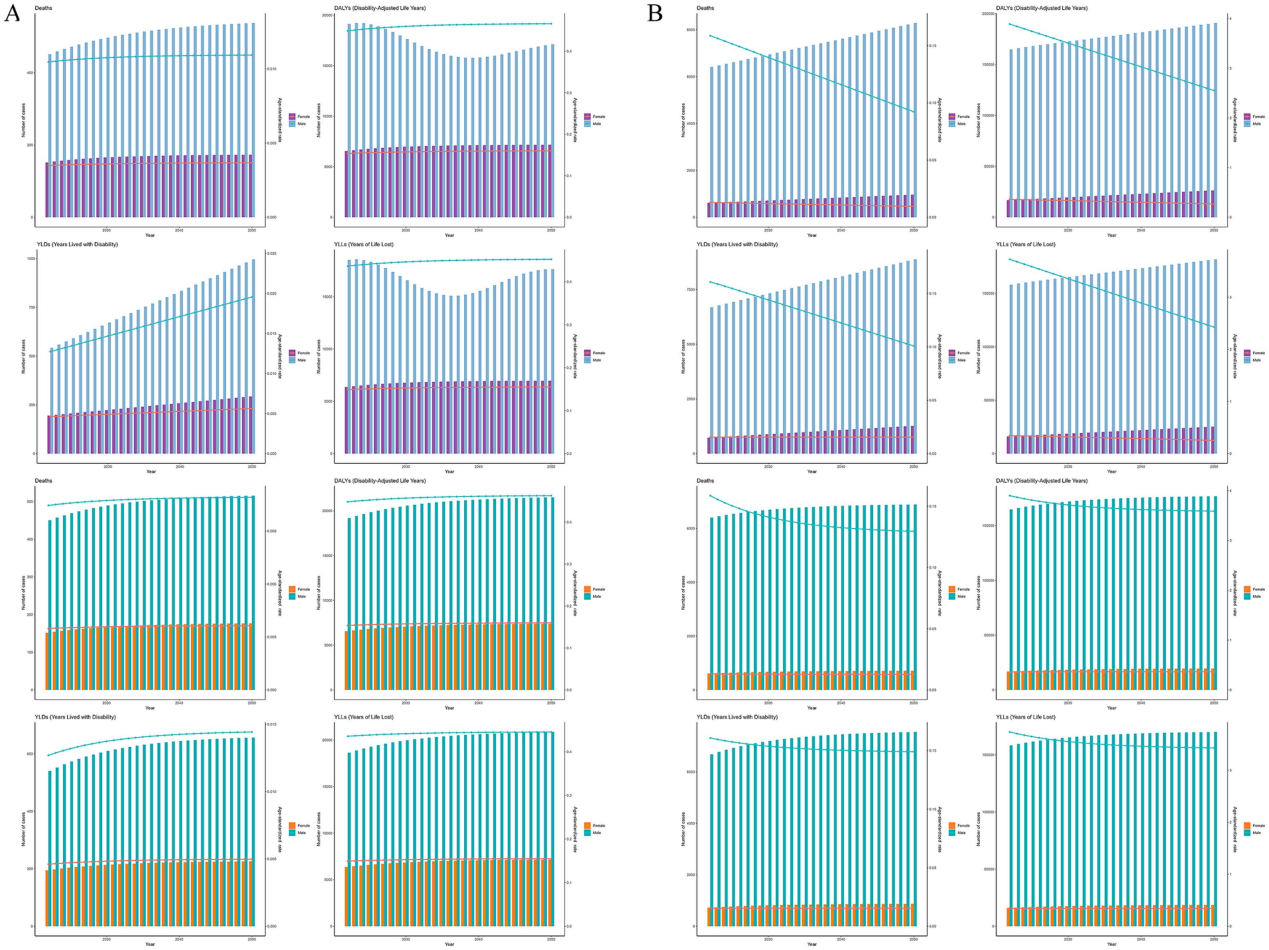
Forecasted trends of occupational carcinogens-attributable deaths, DALYs, YLLs, and YLDs for **(A)** nasopharynx cancer and **(B)** larynx cancer from 2022 to 2050 using ARIMA (the first row) and ES (the second row) models. DALYs, disability-adjusted life years; YLLs, years of life lost; YLDs, years lived with disability; ASMR, age-standardized mortality rate; ASDR, age-standardized DALY rate; ARIMA, autoregressive integrated moving average; ES, exponential smoothing.

For nasopharyngeal cancer, projections using both ARIMA and ES models indicate that by 2050, the number and ASR of deaths and DALYs attributable to occupational carcinogens will remain generally stable, with slight increases. However, the ARIMA model specifically predicts a slight decrease in DALYs number. For larynx cancer, projections using ARIMA and ES models indicate that by 2050, the number of deaths and DALYs attributable to occupational carcinogens will continue to increase, whereas their corresponding age-standardized rates (ASR) will decrease (Figure 5).

While the main focus is on deaths and DALYs, years of life lost (YLLs) and years lived with disability (YLDs) were also analyzed following GBD 2021 methods which are presented in Figures 1, 5, Supplementary Figures 1, 2, 4, 5.

## Discussion

The global burden of occupational carcinogen-attributable nasopharyngeal and laryngeal cancers from 1990 to 2021 highlights a paradox of progress and persistent public health challenges. While age-standardized death and DALYs rates for laryngeal cancer have declined significantly by 42.9 and 42.7% respectively, the ASDR for nasopharyngeal cancer has remained largely unchanged, despite a 33.3% reduction in its DALYs rate. This trend reflects the dual impact of epidemiological advancements and systemic disparities. As previously documented (14), improved occupational safety regulations have contributed to reduced rates in high-SDI regions. However, the rising absolute burden underscores demographic drivers such as population growth and workforce aging, which continue to offset these gains. Pronounced disparities exist across sex, age, and geographic regions, with males and older populations disproportionately affected. These findings reinforce the need for context-specific, evidence-based interventions that address the persistent occupational risks, regulatory gaps, and demographic pressures driving these malignancies.

Both cancers exhibit pronounced gender disparities, with males consistently experiencing higher attributable burdens across global regions. This pattern is consistent with existing findings that link male predominance to occupational segregation, as men are overrepresented in high-risk industries such as manufacturing, mining, and construction (15). These gender-specific trends may further reflect both occupational and biological vulnerability differentials. While structural workforce patterns explain much of this disparity, previous studies have also highlighted the potential influence of sex-related biological factors—such as immune response, hormonal regulation, and carcinogen metabolism—on individual susceptibility to carcinogenesis (16, 17). Importantly, although both cancers share occupational determinants, their etiologies diverge in notable ways. In nasopharyngeal carcinogenesis, it is plausible that latent Epstein–Barr virus (EBV) infection interacts synergistically with long-term occupational exposures, particularly in enclosed or poorly ventilated workplace environments commonly seen in high-incidence regions (18). In contrast, laryngeal cancer is more strongly associated with cumulative exposure to occupational carcinogens, and its risk profile is further exacerbated by behavioral cofactors such as tobacco smoking (19).

Age disparities also play a critical role in the epidemiological patterns of these cancers. Nasopharyngeal cancer predominantly affects middle-aged adults, with a peak burden observed among individuals aged 45–49 years. This reflects a critical “exposure window,” where cumulative occupational exposures may interact with latent viral infections, which could be reactivated under chronic exposure conditions (20). In regions with a high prevalence of EBV and significant woodworking industries, this interplay between viral and chemical carcinogenesis is especially evident, further reinforcing the need for comprehensive workplace exposure controls and targeted screening programs (21, 22). Laryngeal cancer is more prevalent among older adults, peaking in the 60–74 age group. This pattern is indicative of the long latency period associated with cumulative occupational exposures in high-risk industries where many affected individuals have had decades of cumulative exposure, exacerbated by prolonged employment in hazardous occupations (23). The predominance of older males in high-risk industries underscores the importance of integrated occupational health policies that include smoking cessation programs and post-exposure health monitoring (24).

The burden of occupational carcinogen-attributable nasopharyngeal and laryngeal cancers varies significantly across regions, reflecting differences in industrial policies, occupational safety regulations, economic development, and healthcare infrastructure. In high-income regions, such as North America and Western Europe, stringent occupational safety regulations, including asbestos bans and diesel exhaust controls, have led to significant reductions in age-standardized rates of both cancers (25). However, these achievements are tempered by persistently high absolute burdens, largely due to legacy exposures and aging populations (26). Western Europe, in particular, continues to report elevated ASRs for laryngeal cancer, highlighting the long latency of occupational carcinogenic effects and the need for sustained epidemiological surveillance (27). Low- and middle-income countries (LMICs) face substantial challenges stemming from rapid industrialization and insufficient regulatory frameworks. Countries such as Kenya and Paraguay have seen dramatic increases in occupational carcinogen-attributable laryngeal cancer, with death rising by 500 and 400%, respectively. These surges reflect gaps in occupational safety enforcement, widespread exposure to unregulated industrial pollutants, and the lack of protective measures in rapidly expanding industries (28). Nasopharyngeal cancer burdens in these low-resource settings remain comparatively stable, likely due to regionally limited exposure scenarios and varying co-carcinogenic factors such as pollution from coal burning and human papillomaviruses (HPVs) (29, 30).

Middle-income regions exhibit complex epidemiological patterns, with some nations experiencing high burdens due to occupational exposure dynamics. In East Asia, particularly China and Malaysia, nasopharyngeal cancer ASRs remain notably high, driven by furniture industries and the interplay of chemical and viral carcinogenic factors (6). Laryngeal cancer burdens in parts of Latin America, including Brazil and Mexico, remain elevated due to ongoing exposure to mining-related carcinogens, poor regulatory enforcement, and informal labor market conditions (31, 32). Addressing these disparities requires a regionally tailored approach. In high-resource settings, continued monitoring of historical exposure cohorts and enhanced occupational health surveillance are essential, while in LMICs, immediate interventions should focus on occupational hazard recognition, provision of protective equipment, and the establishment of enforceable safety regulations.

Decomposition analysis provides critical insights into the driving factors behind the observed trends in occupational carcinogen-attributable nasopharyngeal and laryngeal cancer burdens. For NPC, population growth dominated global death increases, with females disproportionately affected, reflecting unregulated industrialization in low-resource settings where occupational exposures intersect with rapid demographic expansion (33). Epidemiological improvements, such as enhanced workplace safety protocols, reduced death globally, increased deaths in high-middle SDI regions. This reversal suggests emerging risks in advanced industries, such as formaldehyde use in composite material manufacturing, which may outpace current regulatory frameworks (34). Aging played a modest global role but was amplified in middle SDI regions, underscoring prolonged exposures in aging workforces within industrializing economies like China, where delayed retirement policies exacerbate cumulative risks (35, 36). For LC, high SDI regions demonstrated the efficacy of regulations, with epidemiological changes reducing LC death by 167.39%, yet persistent burdens from legacy asbestos exposures emphasize the need for long-term surveillance (37, 38). In low SDI regions, population growth and weak regulatory enforcement drove rising LC death, as seen in Kenya’s unregulated construction sector (39).

Despite the strengths of the GBD methodology, several limitations should be acknowledged. The accuracy of occupational carcinogen-attributable burden estimates depends on the quality of underlying data, which can vary substantially across countries. In regions with limited occupational exposure surveillance or underdeveloped cancer registries, estimates may rely heavily on model assumptions and extrapolations from comparable settings. Additionally, the exposure-response relationships used in the GBD study are derived from historical data, which may not fully capture current exposure profiles in rapidly industrializing regions.

Nonetheless, this study also possesses several methodological strengths. The use of standardized definitions and modeling techniques from the GBD 2021 framework ensures comparability of estimates across geographies and time. The integration of decomposition analysis enables the identification of specific demographic or epidemiological drivers underlying observed burden trends. Moreover, the application of validated time-series forecasting models (ARIMA and exponential smoothing), along with diagnostic checks and uncertainty intervals, allows for credible future projections. Together, these methods offer a robust foundation for informing policy interventions aimed at mitigating occupational cancer risks.

The distinct occupational and mechanistic profiles of these cancers necessitate targeted prevention strategies. Improved ventilation, routine workplace monitoring of carcinogenic agents such as benzene and formaldehyde, and integration of EBV screening into occupational health programs are essential (40, 41). Existing regulations inadequately address the viral-chemical interaction hypothesis, which needs further research especially in chemically exposed workers.

While our analysis was limited to occupational carcinogens captured in the GBD 2021 framework, we acknowledge that lifestyle-related factors, such as poor sleep, sedentary behavior, psychological stress, and unhealthy dietary patterns, may influence nasopharyngeal and laryngeal carcinogenesis through pathways involving chronic inflammation, immune suppression, and hormonal dysregulation (42–44). These indirect effects, though not currently quantified in GBD, warrant further investigation for their potential synergism with occupational exposures.

## Conclusion

This study delineates the evolving global burden of occupational carcinogen-attributable laryngeal and nasopharyngeal cancers, marked by a paradoxical rise in absolute cases alongside declining age-standardized rates. These trends underscore the dual forces of demographic expansion and partial success in occupational hazard mitigation, particularly in high-resource regions. Persistent disparities across SDI tiers and sexes emphasize the urgent need for equitable policies targeting low-resource industrializing economies and historically marginalized workforce groups. Moving forward, adaptive surveillance systems integrating real-time exposure monitoring, coupled with context-specific interventions, are critical to curbing preventable occupational cancers. For low- and middle-income countries, priorities should include strengthening occupational health surveillance, enforcing exposure-specific regulations, and incorporating workplace safety into vocational training. Addressing data gaps in informal sectors and refining predictive models to capture dynamic industrial shifts will be pivotal in shaping future prevention strategies.

## Data Availability

The datasets presented in this study can be found in online repositories. The names of the repository/repositories and accession number(s) can be found at: https://ghdx.healthdata.org/gbd-2021/sources.
