# Supplementary material for: Global, regional, and national burden and projections to 2050 of occupational carcinogen-attributable nasopharyngeal and laryngeal cancer: a comprehensive analysis from the GBD 2021 study
Source: Front Public Health. 2025 Jul 4;13:1615378. doi: 10.3389/fpubh.2025.1615378 (PMC12270855; doi:10.3389/fpubh.2025.1615378)
Supplement: Supplementary file 1 [file Data_Sheet_1.doc]

***Supplementary Material***

**Supplementary Figures**

**
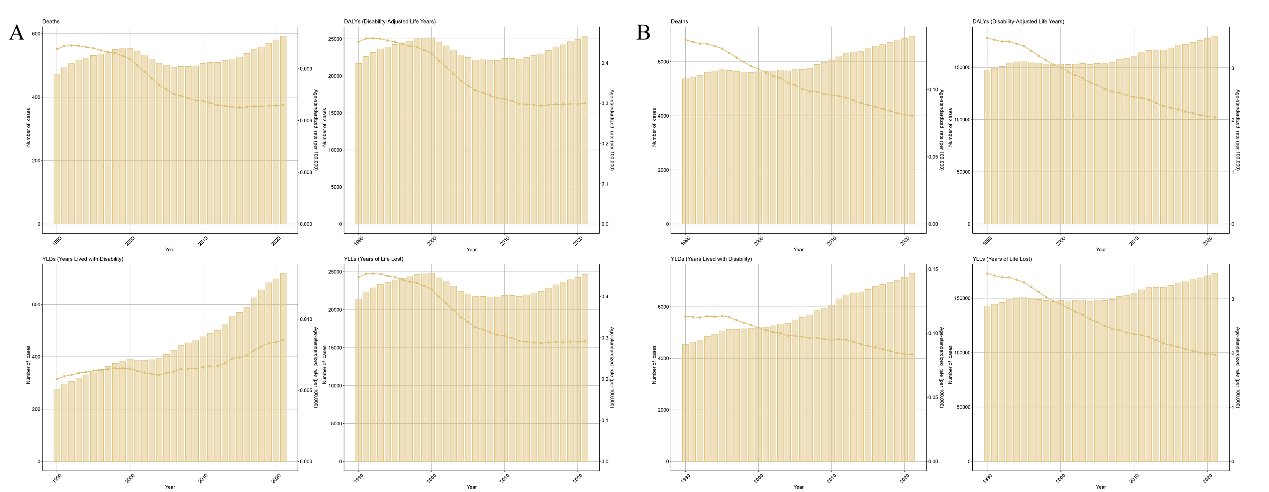
**

**Supplementary Figure 1.** Trends of global occupational carcinogens-attributable deaths, DALYs, YLLs, and YLDs for nasopharynx and larynx cancers, 1990–2021. Abbreviations: DALYs, disability-adjusted life years; YLLs, years of life lost; YLDs, years lived with disability.


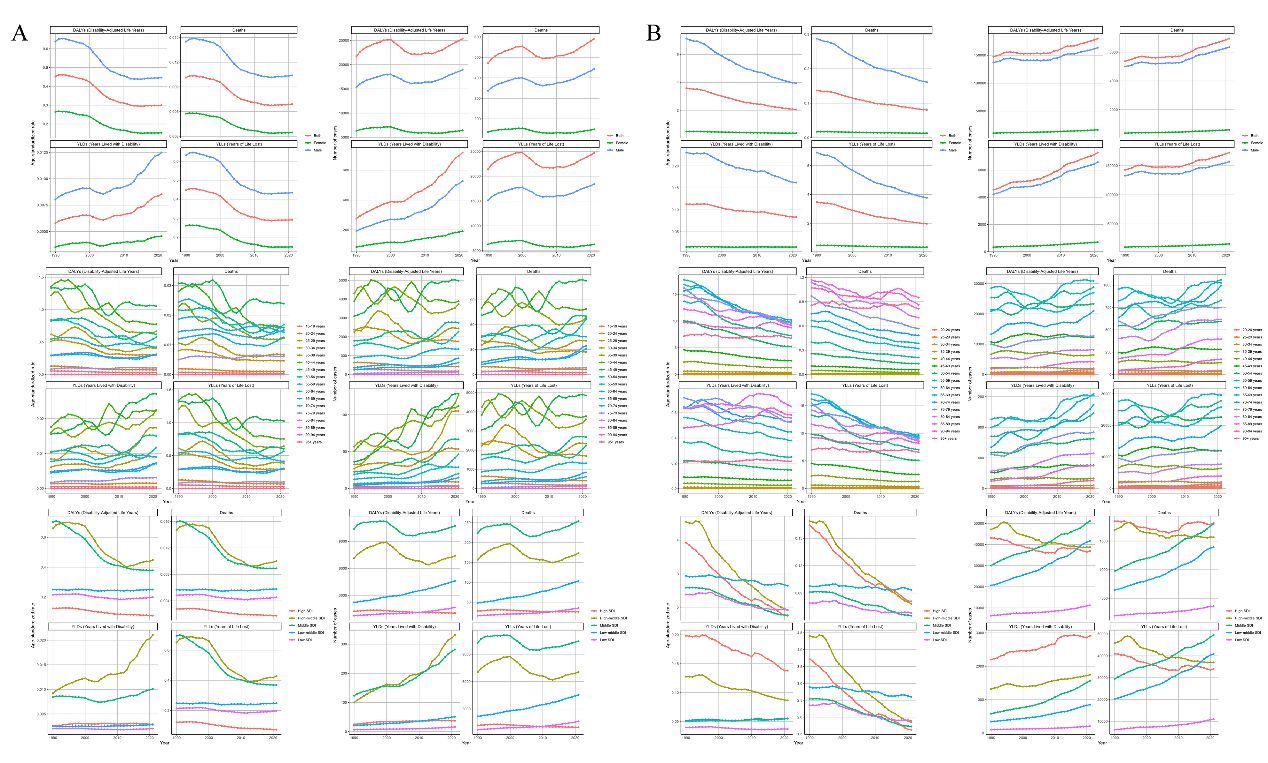


**Supplementary Figure 2.** Trends of occupational carcinogens-attributable deaths, DALYs, YLLs, and YLDs for **(A)** nasopharynx cancer and **(B)** larynx cancer by sex, age group and SDI region, 1990–2021. Abbreviations: DALYs, disability-adjusted life years; YLLs, years of life lost; YLDs, years lived with disability; SDI, Socio-Demographic Index.


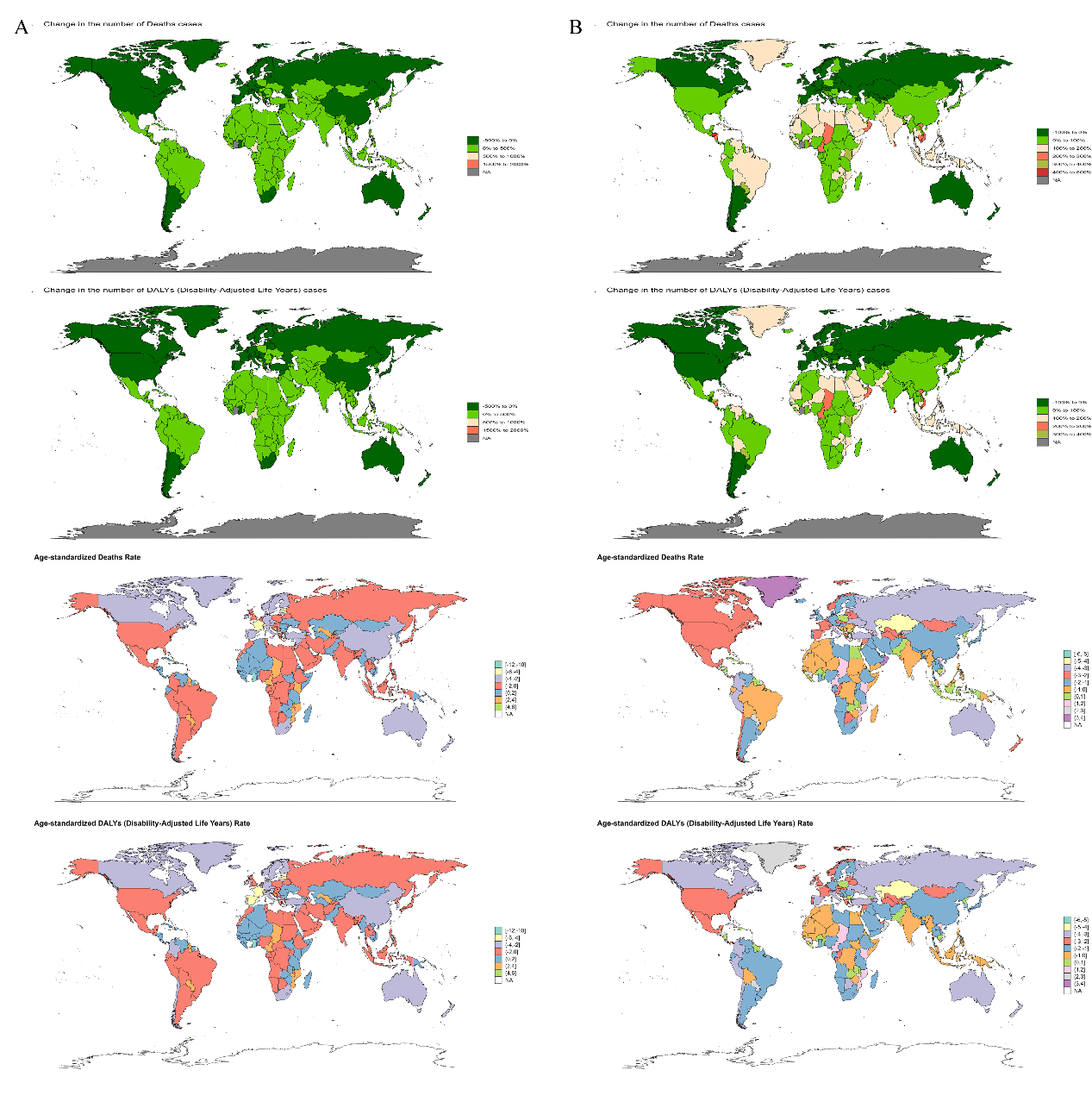


**Supplementary Figure 3.** Absolute changes and EAPC in occupational carcinogens-attributable deaths and DALYs for **(A)** nasopharynx cancer and **(B)** larynx cancer across countries and territories, 1990–2021. Abbreviations: DALYs, disability-adjusted life years; EAPC, estimated annual percentage change.


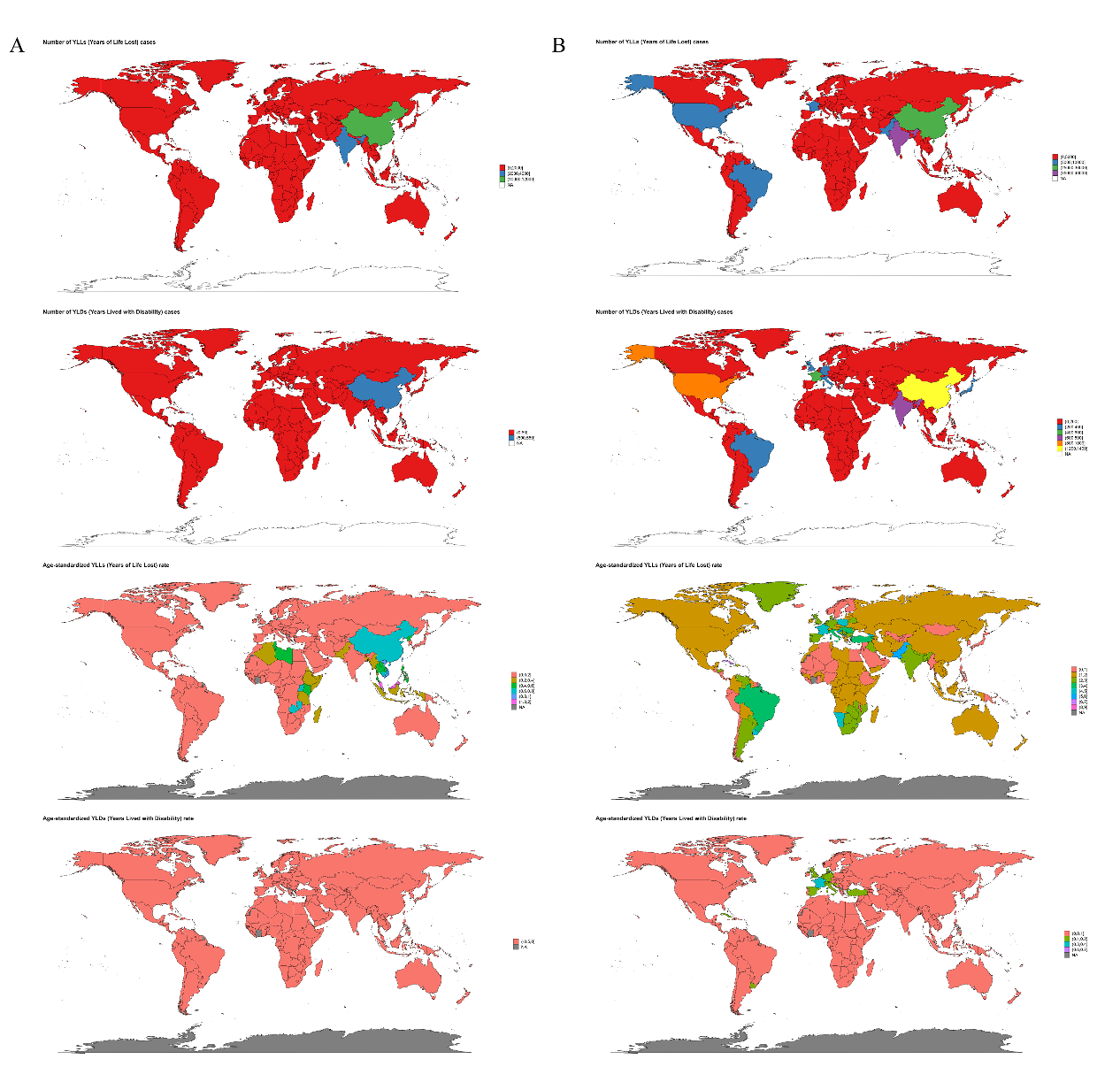
 **Supplementary Figure 4.** Numbers and age-standardized rates of occupational carcinogens-attributable YLLs and YLDs for **(A)** nasopharynx cancer and **(B)** larynx cancer across countries and territories in 2021. Abbreviations: YLLs, years of life lost; YLDs, years lived with disability.


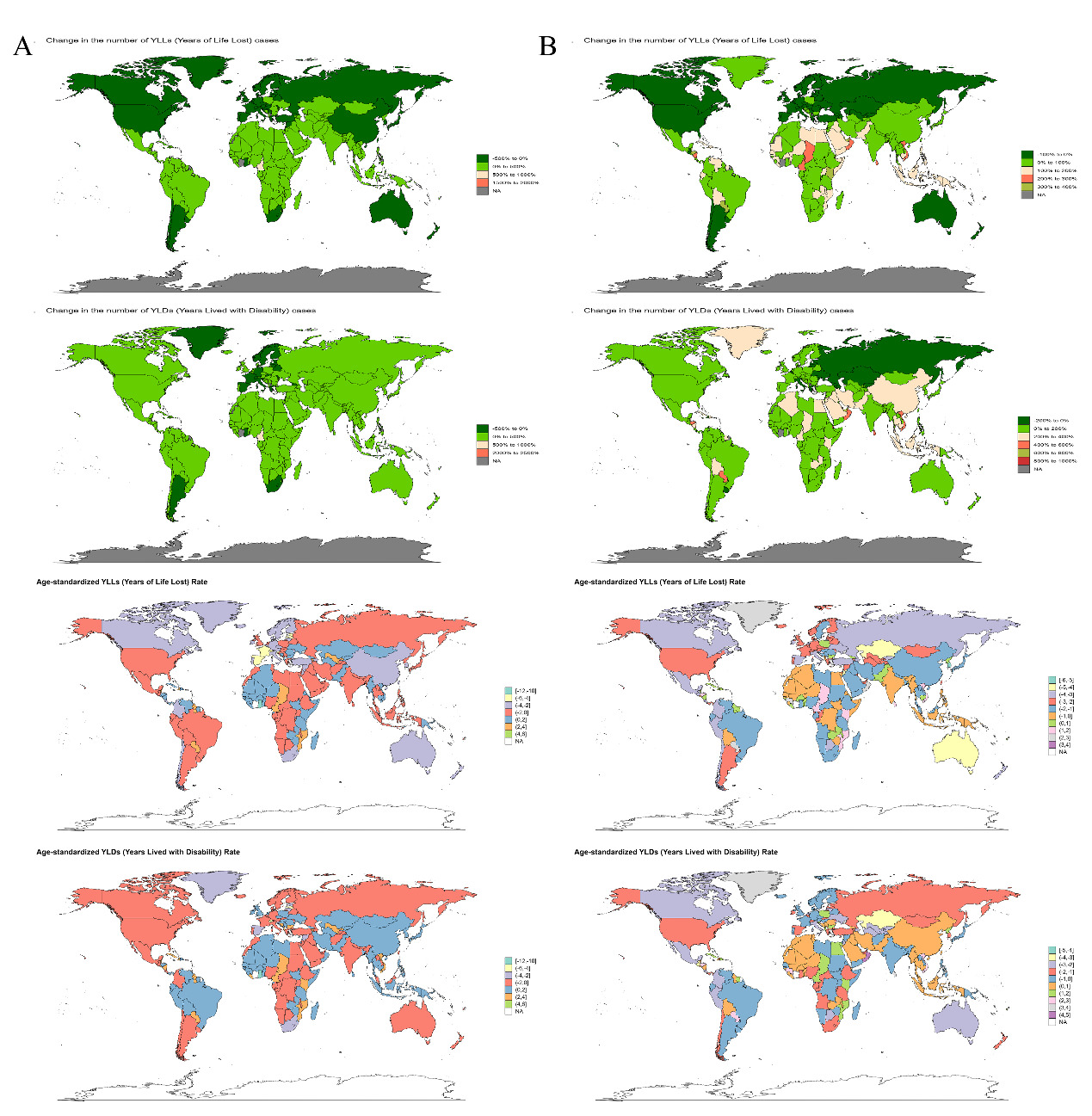


**Supplementary Figure 5.** Trends, absolute changes, and EAPC in occupational carcinogens-attributable YLLs and YLDs for **(A)** nasopharynx cancer and **(B)** larynx cancer across countries and territories, 1990–2021. Abbreviations: YLLs, years of life lost; YLDs, years lived with disability; EAPC, estimated annual percentage change.

**Supplementary Tables**

**Supplementary Table 1. Global Numbers, ASR, and Trends in Occupational Carcinogen-Attributable NPC Deaths: 1990–2021.**

| Characteristics | 1990 | | 2021 | | 1990–2021 |
| --- | --- | --- | --- | --- | --- |
|  | Number of deaths  cases (95% UI) | The age-standardized deaths  rate/100000(95% UI) | Number of deaths  cases (95% UI) | The age-standardized deaths  rate/100000(95% UI) | EAPC (95% CI) |
| Global | 472 (316-652) | 0.01 (0.01-0.01) | 592 (401-856) | 0.01 (0-0.01) | -1.38 (-1.54--1.23) |
| **Sex** | | | | | |
| Female | 135 (77-207) | 0.01 (0-0.01) | 149 (89-229) | 0 (0-0.01) | -2.19 (-2.4--1.98) |
| Male | 337 (193-503) | 0.01 (0.01-0.02) | 443 (264-681) | 0.01 (0.01-0.02) | -1.55 (-1.74--1.36) |
| **Age** | | | | | |
| 15-19 years | 4 (1-9) | 0 (0-0) | 2 (1-5) | 0 (0-0) | -2.45 (-2.64--2.25) |
| 20-24 years | 9 (2-20) | 0 (0-0) | 6 (2-13) | 0 (0-0) | -2.34 (-2.59--2.1) |
| 25-29 years | 36 (9-70) | 0.01 (0-0.02) | 27 (6-53) | 0 (0-0.01) | -2.38 (-2.66--2.09) |
| 30-34 years | 41 (10-86) | 0.01 (0-0.02) | 41 (10-88) | 0.01 (0-0.01) | -2.37 (-2.88--1.86) |
| 35-39 years | 80 (19-178) | 0.02 (0.01-0.05) | 68 (16-141) | 0.01 (0-0.03) | -2.44 (-2.66--2.21) |
| 40-44 years | 80 (20-159) | 0.03 (0.01-0.06) | 79 (17-166) | 0.02 (0-0.03) | -2.38 (-2.6--2.15) |
| 45-49 years | 71 (14-160) | 0.03 (0.01-0.07) | 113 (22-264) | 0.02 (0-0.06) | -1.08 (-1.41--0.74) |
| 50-54 years | 46 (10-103) | 0.02 (0-0.05) | 70 (13-153) | 0.02 (0-0.03) | -1.55 (-1.88--1.22) |
| 55-59 years | 45 (10-96) | 0.02 (0.01-0.05) | 67 (14-143) | 0.02 (0-0.04) | -1.91 (-2.18--1.64) |
| 60-64 years | 30 (6-68) | 0.02 (0-0.04) | 46 (9-104) | 0.01 (0-0.03) | -0.94 (-1.15--0.73) |
| 65-69 years | 15 (3-33) | 0.01 (0-0.03) | 34 (8-84) | 0.01 (0-0.03) | -0.86 (-1.21--0.5) |
| 70-74 years | 12 (3-27) | 0.01 (0-0.03) | 31 (7-67) | 0.01 (0-0.03) | -0.44 (-0.66--0.22) |
| 75-79 years | 3 (1-6) | 0.01 (0-0.01) | 8 (2-17) | 0.01 (0-0.01) | 0.37 (0.17-0.58) |
| 80-84 years | 0 | 0 | 0 | 0 | * |
| 85-89 years | 0 | 0 | 0 | 0 | * |
| 90-94 years | 0 | 0 | 0 | 0 | * |

**Continued** **Supplementary Table 1.** Global Numbers, ASR, and Trends in Occupational Carcinogen-Attributable NPC Deaths: 1990–2021.

| Characteristics | 1990 | | 2021 | | 1990–2021 |
| --- | --- | --- | --- | --- | --- |
|  | Number of deaths  cases (95% UI) | The age-standardized deaths  rate/100000(95% UI) | Number of deaths  cases (95% UI) | The age-standardized deaths  rate/100000 (95% UI) | EAPC (95% CI) |
| 95+ years | 0 | 0 | 0 | 0 | * |
| **SDI regions** | | | | | |
| High-middle SDI | 157 (103-222) | 0.01 (0.01-0.02) | 174 (113-267) | 0.01 (0.01-0.02) | -2.06 (-2.34--1.77) |
| High SDI | 28 (18-38) | 0 (0-0) | 26 (17-37) | 0 (0-0) | -1.81 (-1.94--1.68) |
| Low-middle SDI | 48 (30-69) | 0.01 (0-0.01) | 103 (68-146) | 0.01 (0-0.01) | 0.01 (-0.05-0.06) |
| Low SDI | 16 (10-23) | 0 (0-0.01) | 35 (22-54) | 0 (0-0.01) | -0.51 (-0.64--0.38) |
| Middle SDI | 224 (150-310) | 0.02 (0.01-0.02) | 254 (169-371) | 0.01 (0.01-0.01) | -2.3 (-2.52--2.08) |
| **GBD regions** | | | | | |
| Advanced Health System | 24 (16-34) | 0 (0-0) | 22 (15-32) | 0 (0-0) | -1.34 (-1.63--1.04) |
| Africa | 18 (12-25) | 0 (0-0.01) | 45 (28-66) | 0 (0-0.01) | 0.07 (-0.45-0.6) |
| African Region | 14 (10-20) | 0 (0-0.01) | 38 (24-57) | 0 (0-0.01) | 0.17 (-0.4-0.74) |
| America | 8 (5-10) | 0 (0-0) | 12 (9-17) | 0 (0-0) | -0.11 (-0.27-0.05) |
| Andean Latin America | 0 (0-0) | 0 (0-0) | 1 (0-1) | 0 (0-0) | 0.42 (0.09-0.74) |
| Asia | 437 (293-605) | 0.02 (0.01-0.02) | 527 (356-769) | 0.01 (0.01-0.01) | -1.63 (-1.82--1.44) |
| Australasia | 0 (0-0) | 0 (0-0) | 0 (0-0) | 0 (0-0) | -1.95 (-2.22--1.67) |
| Basic Health System | 370 (248-510) | 0.02 (0.01-0.03) | 415 (274-628) | 0.01 (0.01-0.02) | -1.8 (-2.02--1.58) |
| Caribbean | 1 (0-1) | 0 (0-0) | 2 (1-2) | 0 (0-0) | 2.11 (1.95-2.26) |
| Central Africa | 1 (1-1) | 0 (0-0) | 2 (1-3) | 0 (0-0) | -0.1 (-0.74-0.53) |
| Central Asia | 1 (1-1) | 0 (0-0) | 2 (2-3) | 0 (0-0) | 1.49 (1.26-1.72) |
| Central Europe | 1 (1-1) | 0 (0-0) | 1 (1-2) | 0 (0-0) | 0.1 (-0.31-0.51) |
| Central Latin America | 2 (1-2) | 0 (0-0) | 3 (2-5) | 0 (0-0) | -0.09 (-0.38-0.2) |

**Continued** **Supplementary Table 1.** Global Numbers, ASR, and Trends in Occupational Carcinogen-Attributable NPC Deaths: 1990–2021.

| Characteristics | 1990 | | 2021 | | 1990–2021 |
| --- | --- | --- | --- | --- | --- |
|  | Number of deaths  cases (95% UI) | The age-standardized deaths  rate/100000(95% UI) | Number of deaths  cases (95% UI) | The age-standardized deaths  rate/100000 (95% UI) | Number of deaths  cases (95% UI) |
| Central Sub-Saharan Africa | 1 (0-1) | 0 (0-0) | 1 (1-2) | 0 (0-0) | -0.45 (-1.09-0.2) |
| Commonwealth High Income | 1 (1-2) | 0 (0-0) | 1 (1-1) | 0 (0-0) | -1.62 (-1.93--1.31) |
| Commonwealth Low Income | 9 (6-14) | 0.01 (0-0.01) | 24 (14-40) | 0.01 (0-0.01) | 0.55 (0.01-1.09) |
| Commonwealth Middle Income | 66 (42-95) | 0.01 (0-0.01) | 119 (79-169) | 0.01 (0-0.01) | -0.61 (-0.9--0.33) |
| East Asia | 331 (222-461) | 0.03 (0.02-0.04) | 314 (200-488) | 0.02 (0.01-0.02) | -2.1 (-2.42--1.77) |
| East Asia & Pacific - WB | 375 (251-515) | 0.02 (0.02-0.03) | 413 (274-625) | 0.01 (0.01-0.02) | -1.61 (-1.88--1.35) |
| Eastern Africa | 8 (5-11) | 0.01 (0-0.01) | 22 (14-33) | 0.01 (0.01-0.01) | 0.3 (-0.36-0.96) |
| Eastern Europe | 1 (1-2) | 0 (0-0) | 1 (1-2) | 0 (0-0) | -0.58 (-0.96--0.2) |
| Eastern Mediterranean Region | 10 (6-14) | 0 (0-0.01) | 29 (20-42) | 0 (0-0.01) | 0.64 (0.25-1.03) |
| Eastern Sub-Saharan Africa | 8 (5-12) | 0.01 (0-0.01) | 24 (15-38) | 0.01 (0.01-0.01) | 0.42 (-0.26-1.1) |
| Europe | 9 (6-13) | 0 (0-0) | 8 (5-11) | 0 (0-0) | -1.32 (-1.62--1.02) |
| Europe & Central Asia - WB | 10 (7-15) | 0 (0-0) | 10 (7-14) | 0 (0-0) | -0.81 (-1.07--0.54) |
| European Region | 10 (7-15) | 0 (0-0) | 10 (7-14) | 0 (0-0) | -0.81 (-1.08--0.55) |
| High-income Asia Pacific | 2 (1-2) | 0 (0-0) | 2 (1-2) | 0 (0-0) | -1.04 (-1.54--0.54) |
| High-income North America | 2 (1-3) | 0 (0-0) | 2 (1-2) | 0 (0-0) | -1.78 (-2.07--1.49) |

**Continued** **Supplementary Table 1.** Global Numbers, ASR, and Trends in Occupational Carcinogen-Attributable NPC Deaths: 1990–2021.

| Characteristics | 1990 | | 2021 | | 1990–2021 |
| --- | --- | --- | --- | --- | --- |
|  | Number of deaths  cases (95% UI) | The age-standardized deaths  rate/100000(95% UI) | Number of deaths  cases (95% UI) | The age-standardized deaths  rate/100000 (95% UI) | Number of deaths  cases (95% UI) |
| Latin America & Caribbean - WB | 6 (4-8) | 0 (0-0) | 11 (7-14) | 0 (0-0) | 0.06 (-0.19-0.3) |
| Limited Health System | 77 (48-109) | 0.01 (0-0.01) | 150 (100-214) | 0.01 (0-0.01) | -0.31 (-0.65-0.03) |
| Middle East & North Africa - WB | 6 (4-9) | 0 (0-0.01) | 14 (9-21) | 0 (0-0) | -0.11 (-0.43-0.2) |
| Minimal Health System | 2 (1-2) | 0 (0-0) | 4 (3-7) | 0 (0-0) | 0.04 (-0.63-0.72) |
| North Africa and Middle East | 10 (6-14) | 0 (0-0.01) | 18 (11-26) | 0 (0-0) | -0.65 (-0.96--0.35) |
| North America | 2 (1-3) | 0 (0-0) | 2 (1-2) | 0 (0-0) | -1.77 (-2.07--1.48) |
| Northern Africa | 4 (3-6) | 0.01 (0-0.01) | 9 (5-14) | 0 (0-0.01) | 0.17 (-0.11-0.46) |
| Oceania | 0 (0-0) | 0 (0-0) | 0 (0-1) | 0 (0-0) | 0.51 (0.06-0.96) |
| Region of the Americas | 8 (5-10) | 0 (0-0) | 12 (9-17) | 0 (0-0) | -0.11 (-0.27-0.05) |
| South-East Asia Region | 79 (51-113) | 0.01 (0-0.01) | 137 (89-200) | 0.01 (0-0.01) | -0.4 (-0.61--0.19) |
| South Asia | 59 (37-86) | 0.01 (0-0.01) | 106 (69-154) | 0.01 (0-0.01) | -0.46 (-0.72--0.19) |
| South Asia - WB | 60 (37-87) | 0.01 (0-0.01) | 107 (70-156) | 0.01 (0-0.01) | -0.45 (-0.72--0.19) |
| Southeast Asia | 43 (29-59) | 0.01 (0.01-0.02) | 99 (65-138) | 0.01 (0.01-0.02) | 0.67 (0.49-0.85) |
| Southern Africa | 2 (1-3) | 0 (0-0) | 4 (2-7) | 0 (0-0) | 0.06 (-0.41-0.53) |
| Southern Latin America | 1 (1-2) | 0 (0-0) | 1 (1-1) | 0 (0-0) | -1.74 (-1.94--1.55) |
| Southern Sub-Saharan Africa | 1 (1-1) | 0 (0-0) | 1 (1-2) | 0 (0-0) | -1.49 (-1.84--1.14) |
| Sub-Saharan Africa - WB | 14 (9-19) | 0 (0-0.01) | 36 (22-53) | 0 (0-0.01) | 0.06 (-0.53-0.65) |
| Tropical Latin America | 2 (1-3) | 0 (0-0) | 4 (3-6) | 0 (0-0) | 0.27 (-0.13-0.68) |

**Continued** **Supplementary Table 1.** Global Numbers, ASR, and Trends in Occupational Carcinogen-Attributable NPC Deaths: 1990–2021.

| Characteristics | 1990 | | 2021 | | 1990–2021 |
| --- | --- | --- | --- | --- | --- |
|  | Number of deaths  cases (95% UI) | The age-standardized deaths  rate/100000(95% UI) | Number of deaths  cases (95% UI) | The age-standardized deaths  rate/100000 (95% UI) | Number of deaths  cases (95% UI) |
| Western Africa | 3 (2-5) | 0 (0-0) | 8 (4-12) | 0 (0-0) | -0.36 (-0.95-0.24) |
| Western Europe | 4 (3-6) | 0 (0-0) | 2 (2-3) | 0 (0-0) | -2.68 (-3.06--2.3) |
| Western Pacific Region | 341 (229-474) | 0.02 (0.02-0.03) | 357 (232-549) | 0.01 (0.01-0.02) | -1.76 (-2.06--1.46) |
| Western Sub-Saharan Africa | 3 (2-5) | 0 (0-0) | 8 (5-13) | 0 (0-0) | -0.29 (-0.88-0.31) |

**Supplementary Table 2.** Numbers, ASR, and Trends in Occupational Carcinogen-Attributable NPC Deaths across countries and territories: 1990–2021.

| Characteristics | 1990 | | 2021 | | 1990–2021 |
| --- | --- | --- | --- | --- | --- |
|  | Number of deaths  cases (95% UI) | The age-standardized deaths  rate/100000 (95% UI) | Number of deaths  cases (95% UI) | The age-standardized deaths  rate/100000 (95% UI) | EAPC (95% CI) |
| Afghanistan | 0 (0-0) | 0 (0-0) | 0 (0-1) | 0 (0-0) | -1.06 (-1.78--0.33) |
| Albania | 0 (0-0) | 0 (0-0) | 0 (0-0) | 0 (0-0) | 2.28 (1.95-2.6) |
| Algeria | 1 (1-2) | 0.01 (0.01-0.01) | 4 (2-6) | 0.01 (0-0.01) | 1.12 (0.77-1.48) |
| American Samoa | 0 (0-0) | 0.01 (0.01-0.01) | 0 (0-0) | 0.01 (0.01-0.01) | 0.98 (0.65-1.3) |
| Andorra | 0 (0-0) | 0 (0-0) | 0 (0-0) | 0 (0-0) | -1.37 (-1.82--0.93) |
| Angola | 0 (0-0) | 0 (0-0) | 0 (0-0) | 0 (0-0) | -0.94 (-1.62--0.26) |
| Antigua and Barbuda | 0 (0-0) | 0 (0-0) | 0 (0-0) | 0 (0-0) | 1.04 (0.84-1.23) |
| Argentina | 1 (1-1) | 0 (0-0) | 1 (0-1) | 0 (0-0) | -1.72 (-1.9--1.54) |
| Armenia | 0 (0-0) | 0 (0-0) | 0 (0-0) | 0 (0-0) | 3.09 (2.67-3.51) |
| Australia | 0 (0-0) | 0 (0-0) | 0 (0-0) | 0 (0-0) | -1.95 (-2.23--1.67) |
| Austria | 0 (0-0) | 0 (0-0) | 0 (0-0) | 0 (0-0) | -1.79 (-2.2--1.38) |
| Azerbaijan | 0 (0-0) | 0 (0-0) | 0 (0-0) | 0 (0-0) | -1.48 (-1.69--1.28) |
| Bahamas | 0 (0-0) | 0 (0-0) | 0 (0-0) | 0 (0-0) | 1.62 (1.38-1.87) |
| Bahrain | 0 (0-0) | 0 (0-0.01) | 0 (0-0) | 0 (0-0) | -1.68 (-1.93--1.42) |
| Bangladesh | 5 (3-8) | 0.01 (0-0.01) | 11 (5-21) | 0.01 (0-0.01) | 0.35 (-0.08-0.78) |
| Barbados | 0 (0-0) | 0 (0-0) | 0 (0-0) | 0 (0-0) | 1.41 (1.07-1.75) |
| Belarus | 0 (0-0) | 0 (0-0) | 0 (0-0) | 0 (0-0) | -0.98 (-1.4--0.55) |
| Belgium | 0 (0-0) | 0 (0-0) | 0 (0-0) | 0 (0-0) | -1.64 (-2.05--1.24) |
| Belize | 0 (0-0) | 0 (0-0) | 0 (0-0) | 0 (0-0) | 3.01 (2.39-3.63) |
| Benin | 0 (0-0) | 0 (0-0) | 0 (0-0) | 0 (0-0) | 1.27 (0.6-1.95) |

**Continued** **Supplementary Table 2.** Numbers, ASR, and Trends in Occupational Carcinogen-Attributable NPC Deaths across countries and territories: 1990–2021.

| Characteristics | 1990 | | 2021 | | 1990–2021 |
| --- | --- | --- | --- | --- | --- |
|  | Number of deaths  cases (95% UI) | The age-standardized deaths  rate/100000 (95% UI) | Number of deaths  cases (95% UI) | The age-standardized deaths  rate/100000 (95% UI) | Number of deaths  cases (95% UI) |
| Bermuda | 0 (0-0) | 0 (0-0) | 0 (0-0) | 0 (0-0) | -0.05 (-0.5-0.4) |
| Bhutan | 0 (0-0) | 0.01 (0-0.01) | 0 (0-0) | 0.01 (0-0.01) | 0.09 (-0.28-0.47) |
| Bolivia (Plurinational State of) | 0 (0-0) | 0 (0-0) | 0 (0-0) | 0 (0-0) | 0.51 (0.08-0.94) |
| Bosnia and Herzegovina | 0 (0-0) | 0 (0-0) | 0 (0-0) | 0 (0-0) | 2.69 (2.27-3.11) |
| Botswana | 0 (0-0) | 0 (0-0) | 0 (0-0) | 0 (0-0) | -0.54 (-1.05--0.03) |
| Brazil | 2 (1-3) | 0 (0-0) | 4 (3-6) | 0 (0-0) | 0.24 (-0.17-0.64) |
| Brunei Darussalam | 0 (0-0) | 0 (0-0.01) | 0 (0-0) | 0 (0-0) | 0.19 (-0.08-0.45) |
| Bulgaria | 0 (0-0) | 0 (0-0) | 0 (0-0) | 0 (0-0) | 1.44 (0.99-1.89) |
| Burkina Faso | 0 (0-0) | 0 (0-0) | 0 (0-0) | 0 (0-0) | 1.47 (0.79-2.15) |
| Burundi | 0 (0-0) | 0.01 (0-0.01) | 1 (0-1) | 0.01 (0-0.01) | -0.73 (-1.39--0.07) |
| Cabo Verde | 0 (0-0) | 0 (0-0) | 0 (0-0) | 0 (0-0) | 5.82 (4.52-7.15) |
| Cambodia | 1 (0-1) | 0.01 (0.01-0.02) | 2 (2-4) | 0.02 (0.01-0.02) | 2.02 (1.6-2.45) |
| Cameroon | 0 (0-0) | 0 (0-0) | 0 (0-1) | 0 (0-0) | 2.62 (2.03-3.22) |
| Canada | 0 (0-0) | 0 (0-0) | 0 (0-0) | 0 (0-0) | -2.62 (-2.98--2.25) |
| Central African Republic | 0 (0-0) | 0 (0-0) | 0 (0-0) | 0 (0-0) | -0.86 (-1.46--0.25) |
| Chad | 0 (0-0) | 0 (0-0) | 0 (0-0) | 0 (0-0) | 2.35 (1.57-3.14) |
| Chile | 0 (0-0) | 0 (0-0) | 0 (0-0) | 0 (0-0) | -1.76 (-2.03--1.48) |
| China | 318 (214-446) | 0.03 (0.02-0.04) | 301 (190-469) | 0.02 (0.01-0.02) | -2.12 (-2.45--1.79) |

**Continued** **Supplementary Table 2.** Numbers, ASR, and Trends in Occupational Carcinogen-Attributable NPC Deaths across countries and territories: 1990–2021.

| Characteristics | 1990 | | 2021 | | 1990–2021 |
| --- | --- | --- | --- | --- | --- |
|  | Number of deaths  cases (95% UI) | The age-standardized deaths  rate/100000 (95% UI) | Number of deaths  cases (95% UI) | The age-standardized deaths  rate/100000 (95% UI) | Number of deaths  cases (95% UI) |
| Colombia | 0 (0-1) | 0 (0-0) | 1 (0-1) | 0 (0-0) | -1.32 (-1.52--1.11) |
| Comoros | 0 (0-0) | 0 (0-0.01) | 0 (0-0) | 0.01 (0-0.01) | 0.89 (0.4-1.38) |
| Congo | 0 (0-0) | 0 (0-0) | 0 (0-0) | 0 (0-0) | -0.81 (-1.33--0.3) |
| Cook Islands | 0 (0-0) | 0 (0-0) | 0 (0-0) | 0 (0-0) | 1.32 (1.04-1.61) |
| Costa Rica | 0 (0-0) | 0 (0-0) | 0 (0-0) | 0 (0-0) | -1.37 (-1.7--1.04) |
| Croatia | 0 (0-0) | 0 (0-0) | 0 (0-0) | 0 (0-0) | 0.1 (-0.5-0.7) |
| Cuba | 0 (0-0) | 0 (0-0) | 1 (0-1) | 0 (0-0.01) | 2.37 (2.01-2.73) |
| Cyprus | 0 (0-0) | 0 (0-0) | 0 (0-0) | 0 (0-0) | -1.65 (-1.94--1.36) |
| Czechia | 0 (0-0) | 0 (0-0) | 0 (0-0) | 0 (0-0) | -2.27 (-2.68--1.87) |
| Democratic People's Republic of Korea | 3 (2-4) | 0.01 (0.01-0.02) | 5 (3-7) | 0.01 (0.01-0.02) | 0.56 (0.36-0.75) |
| Democratic Republic of the Congo | 0 (0-1) | 0 (0-0) | 1 (0-1) | 0 (0-0) | -0.21 (-0.87-0.45) |
| Denmark | 0 (0-0) | 0 (0-0) | 0 (0-0) | 0 (0-0) | -2.46 (-2.89--2.03) |
| Djibouti | 0 (0-0) | 0 (0-0.01) | 0 (0-0) | 0.01 (0-0.01) | 1.22 (0.81-1.64) |
| Dominica | 0 (0-0) | 0 (0-0) | 0 (0-0) | 0 (0-0) | 2.73 (2.49-2.97) |
| Dominican Republic | 0 (0-0) | 0 (0-0) | 0 (0-0) | 0 (0-0) | 1.21 (0.91-1.5) |
| Ecuador | 0 (0-0) | 0 (0-0) | 0 (0-0) | 0 (0-0) | 0.58 (0.08-1.07) |
| Egypt | 0 (0-0) | 0 (0-0) | 0 (0-0) | 0 (0-0) | -0.62 (-0.98--0.25) |
| El Salvador | 0 (0-0) | 0 (0-0) | 0 (0-0) | 0 (0-0) | 1.69 (1.34-2.04) |

**Continued** **Supplementary Table 2.** Numbers, ASR, and Trends in Occupational Carcinogen-Attributable NPC Deaths across countries and territories: 1990–2021.

| Characteristics | 1990 | | 2021 | | 1990–2021 |
| --- | --- | --- | --- | --- | --- |
|  | Number of deaths  cases (95% UI) | The age-standardized deaths  rate/100000 (95% UI) | Number of deaths  cases (95% UI) | The age-standardized deaths  rate/100000 (95% UI) | Number of deaths  cases (95% UI) |
| Equatorial Guinea | 0 (0-0) | 0 (0-0) | 0 (0-0) | 0 (0-0) | 0.36 (-0.37-1.09) |
| Eritrea | 0 (0-0) | 0.01 (0.01-0.01) | 0 (0-1) | 0.01 (0.01-0.02) | 0.99 (0.42-1.56) |
| Estonia | 0 (0-0) | 0 (0-0) | 0 (0-0) | 0 (0-0) | -4.57 (-4.91--4.24) |
| Eswatini | 0 (0-0) | 0 (0-0) | 0 (0-0) | 0 (0-0) | 0.56 (-0.19-1.3) |
| Ethiopia | 2 (1-3) | 0.01 (0-0.01) | 5 (3-8) | 0.01 (0-0.01) | -0.68 (-1.38-0.03) |
| Fiji | 0 (0-0) | 0 (0-0) | 0 (0-0) | 0 (0-0) | 0.3 (0.05-0.55) |
| Finland | 0 (0-0) | 0 (0-0) | 0 (0-0) | 0 (0-0) | -2.5 (-2.93--2.07) |
| France | 1 (1-2) | 0 (0-0) | 0 (0-1) | 0 (0-0) | -3.83 (-4.16--3.49) |
| Gabon | 0 (0-0) | 0 (0-0) | 0 (0-0) | 0 (0-0) | -0.76 (-1.2--0.31) |
| Gambia | 0 (0-0) | 0 (0-0) | 0 (0-0) | 0 (0-0) | 1.12 (0.47-1.79) |
| Georgia | 0 (0-0) | 0 (0-0) | 0 (0-0) | 0 (0-0) | 1.17 (0.75-1.6) |
| Germany | 1 (1-1) | 0 (0-0) | 0 (0-1) | 0 (0-0) | -2.27 (-2.8--1.74) |
| Ghana | 0 (0-0) | 0 (0-0) | 0 (0-0) | 0 (0-0) | -10.64 (-11.83--9.43) |
| Greece | 0 (0-0) | 0 (0-0) | 0 (0-0) | 0 (0-0) | -1.4 (-1.82--0.97) |
| Greenland | 0 (0-0) | 0.02 (0.01-0.02) | 0 (0-0) | 0.01 (0-0.01) | -3.05 (-3.32--2.79) |
| Grenada | 0 (0-0) | 0 (0-0) | 0 (0-0) | 0 (0-0.01) | 2.19 (1.88-2.51) |
| Guam | 0 (0-0) | 0.01 (0.01-0.01) | 0 (0-0) | 0.01 (0-0.01) | -0.19 (-0.45-0.07) |
| Guatemala | 0 (0-0) | 0 (0-0) | 0 (0-0) | 0 (0-0) | -0.82 (-1.38--0.25) |
| Guinea | 0 (0-0) | 0 (0-0) | 0 (0-0) | 0 (0-0) | 1.55 (0.94-2.16) |

**Continued** **Supplementary Table 2.** Numbers, ASR, and Trends in Occupational Carcinogen-Attributable NPC Deaths across countries and territories: 1990–2021.

| Characteristics | 1990 | | 2021 | | 1990–2021 |
| --- | --- | --- | --- | --- | --- |
|  | Number of deaths  cases (95% UI) | The age-standardized deaths  rate/100000 (95% UI) | Number of deaths  cases (95% UI) | The age-standardized deaths  rate/100000 (95% UI) | Number of deaths  cases (95% UI) |
| Guinea-Bissau | 0 (0-0) | 0 (0-0) | 0 (0-0) | 0 (0-0) | 1.54 (0.91-2.16) |
| Guyana | 0 (0-0) | 0 (0-0) | 0 (0-0) | 0 (0-0) | 2.84 (2.44-3.24) |
| Haiti | 0 (0-0) | 0 (0-0) | 0 (0-1) | 0 (0-0.01) | 2.52 (2.04-3) |
| Honduras | 0 (0-0) | 0 (0-0) | 0 (0-0) | 0 (0-0) | 1.05 (0.48-1.61) |
| Hungary | 0 (0-0) | 0 (0-0) | 0 (0-0) | 0 (0-0) | -0.21 (-0.65-0.24) |
| Iceland | 0 (0-0) | 0 (0-0) | 0 (0-0) | 0 (0-0) | -1.87 (-2.16--1.58) |
| India | 49 (30-71) | 0.01 (0-0.01) | 76 (48-112) | 0.01 (0-0.01) | -0.89 (-1.13--0.66) |
| Indonesia | 14 (9-21) | 0.01 (0.01-0.01) | 29 (17-48) | 0.01 (0.01-0.02) | 0.37 (0.19-0.54) |
| Iran (Islamic Republic of) | 0 (0-0) | 0 (0-0) | 1 (0-1) | 0 (0-0) | 0.05 (-0.36-0.46) |
| Iraq | 0 (0-0) | 0 (0-0) | 1 (0-1) | 0 (0-0) | -0.83 (-1.33--0.32) |
| Ireland | 0 (0-0) | 0 (0-0) | 0 (0-0) | 0 (0-0) | -1.88 (-2.14--1.63) |
| Israel | 0 (0-0) | 0 (0-0) | 0 (0-0) | 0 (0-0) | -1.31 (-1.51--1.11) |
| Italy | 1 (0-1) | 0 (0-0) | 0 (0-1) | 0 (0-0) | -2.76 (-3.19--2.32) |
| Jamaica | 0 (0-0) | 0 (0-0) | 0 (0-0) | 0 (0-0) | 2.06 (1.61-2.5) |
| Japan | 1 (1-1) | 0 (0-0) | 1 (1-2) | 0 (0-0) | -0.51 (-1.14-0.14) |
| Jordan | 0 (0-0) | 0 (0-0.01) | 0 (0-0) | 0 (0-0) | -2.01 (-2.51--1.51) |
| Kazakhstan | 0 (0-0) | 0 (0-0) | 0 (0-1) | 0 (0-0) | 0.33 (0.15-0.5) |
| Kenya | 1 (1-2) | 0.01 (0-0.01) | 5 (3-8) | 0.01 (0.01-0.02) | 2.49 (1.83-3.17) |
| Kiribati | 0 (0-0) | 0 (0-0) | 0 (0-0) | 0 (0-0) | 3.13 (2.62-3.63) |

**Continued** **Supplementary Table 2.** Numbers, ASR, and Trends in Occupational Carcinogen-Attributable NPC Deaths across countries and territories: 1990–2021.

| Characteristics | 1990 | | 2021 | | 1990–2021 |
| --- | --- | --- | --- | --- | --- |
|  | Number of deaths  cases (95% UI) | The age-standardized deaths  rate/100000 (95% UI) | Number of deaths  cases (95% UI) | The age-standardized deaths  rate/100000 (95% UI) | Number of deaths  cases (95% UI) |
| Kuwait | 0 (0-0) | 0 (0-0) | 0 (0-0) | 0 (0-0) | -2.05 (-2.38--1.71) |
| Kyrgyzstan | 0 (0-0) | 0 (0-0) | 0 (0-0) | 0 (0-0) | 0.91 (0.59-1.24) |
| Lao People's Democratic Republic | 0 (0-1) | 0.01 (0.01-0.02) | 1 (0-1) | 0.01 (0.01-0.02) | -0.28 (-0.74-0.19) |
| Latvia | 0 (0-0) | 0 (0-0) | 0 (0-0) | 0 (0-0) | -4.47 (-4.89--4.05) |
| Lebanon | 0 (0-0) | 0 (0-0) | 0 (0-0) | 0 (0-0) | -0.93 (-1.08--0.79) |
| Lesotho | 0 (0-0) | 0 (0-0) | 0 (0-0) | 0 (0-0) | 2.56 (1.93-3.18) |
| Liberia | 0 (0-0) | 0 (0-0) | 0 (0-0) | 0 (0-0) | 2.07 (1.53-2.6) |
| Libya | 0 (0-1) | 0.01 (0.01-0.02) | 1 (1-2) | 0.01 (0.01-0.02) | 0.42 (0.01-0.82) |
| Lithuania | 0 (0-0) | 0 (0-0) | 0 (0-0) | 0 (0-0) | -3.01 (-3.4--2.62) |
| Luxembourg | 0 (0-0) | 0 (0-0) | 0 (0-0) | 0 (0-0) | -3.89 (-4.26--3.51) |
| Madagascar | 1 (0-1) | 0.01 (0-0.01) | 1 (1-2) | 0.01 (0-0.01) | 0.25 (-0.33-0.83) |
| Malawi | 0 (0-0) | 0 (0-0) | 0 (0-1) | 0 (0-0) | -0.58 (-1.28-0.11) |
| Malaysia | 8 (5-12) | 0.06 (0.04-0.08) | 15 (9-22) | 0.04 (0.03-0.07) | -1.18 (-1.56--0.79) |
| Maldives | 0 (0-0) | 0 (0-0) | 0 (0-0) | 0 (0-0) | -2.56 (-3.03--2.09) |
| Mali | 0 (0-0) | 0 (0-0) | 0 (0-0) | 0 (0-0) | 0.41 (-0.27-1.1) |
| Malta | 0 (0-0) | 0 (0-0) | 0 (0-0) | 0 (0-0) | -1.35 (-1.73--0.96) |
| Marshall Islands | 0 (0-0) | 0 (0-0.01) | 0 (0-0) | 0 (0-0.01) | 0.96 (0.42-1.5) |
| Mauritania | 0 (0-0) | 0 (0-0) | 0 (0-0) | 0 (0-0) | 0.77 (0.25-1.29) |
| Mauritius | 0 (0-0) | 0.01 (0-0.01) | 0 (0-0) | 0.01 (0-0.01) | 0.19 (-0.08-0.47) |

**Continued** **Supplementary Table 2.** Numbers, ASR, and Trends in Occupational Carcinogen-Attributable NPC Deaths across countries and territories: 1990–2021.

| Characteristics | 1990 | | 2021 | | 1990–2021 |
| --- | --- | --- | --- | --- | --- |
|  | Number of deaths  cases (95% UI) | The age-standardized deaths  rate/100000 (95% UI) | Number of deaths  cases (95% UI) | The age-standardized deaths  rate/100000 (95% UI) | Number of deaths  cases (95% UI) |
| Mexico | 1 (0-1) | 0 (0-0) | 1 (1-2) | 0 (0-0) | -0.07 (-0.38-0.25) |
| Micronesia (Federated States of) | 0 (0-0) | 0.01 (0-0.01) | 0 (0-0) | 0 (0-0.01) | 0.2 (-0.24-0.63) |
| Monaco | 0 (0-0) | 0 (0-0) | 0 (0-0) | 0 (0-0) | -0.85 (-1.31--0.38) |
| Mongolia | 0 (0-0) | 0 (0-0) | 0 (0-0) | 0 (0-0) | 1.74 (1.41-2.06) |
| Montenegro | 0 (0-0) | 0 (0-0) | 0 (0-0) | 0 (0-0) | 0.52 (0.23-0.81) |
| Morocco | 1 (1-2) | 0.01 (0-0.01) | 2 (1-4) | 0.01 (0-0.01) | -0.49 (-0.74--0.25) |
| Mozambique | 0 (0-0) | 0 (0-0) | 0 (0-0) | 0 (0-0) | 2.37 (1.59-3.16) |
| Myanmar | 2 (1-3) | 0.01 (0-0.01) | 4 (3-7) | 0.01 (0-0.01) | 1.1 (0.82-1.39) |
| Namibia | 0 (0-0) | 0 (0-0) | 0 (0-0) | 0 (0-0) | -0.76 (-1.25--0.27) |
| Nauru | 0 (0-0) | 0.01 (0-0.01) | 0 (0-0) | 0.01 (0-0.01) | -0.12 (-0.59-0.34) |
| Nepal | 1 (1-1) | 0.01 (0-0.01) | 1 (1-2) | 0 (0-0.01) | -0.58 (-0.96--0.19) |
| Netherlands | 0 (0-0) | 0 (0-0) | 0 (0-0) | 0 (0-0) | -1.02 (-1.48--0.56) |
| New Zealand | 0 (0-0) | 0 (0-0) | 0 (0-0) | 0 (0-0) | -1.92 (-2.2--1.63) |
| Nicaragua | 0 (0-0) | 0 (0-0) | 0 (0-0) | 0 (0-0) | 2.49 (1.92-3.07) |
| Niger | 0 (0-0) | 0 (0-0) | 0 (0-0) | 0 (0-0) | 0.55 (-0.19-1.31) |
| Nigeria | 3 (2-4) | 0 (0-0.01) | 6 (4-10) | 0 (0-0.01) | -0.45 (-1.04-0.13) |
| Niue | 0 (0-0) | 0 (0-0.01) | 0 (0-0) | 0 (0-0.01) | -0.13 (-0.31-0.06) |
| North Macedonia | 0 (0-0) | 0 (0-0) | 0 (0-0) | 0 (0-0) | 1.48 (1.14-1.82) |

**Continued** **Supplementary Table 2.** Numbers, ASR, and Trends in Occupational Carcinogen-Attributable NPC Deaths across countries and territories: 1990–2021.

| Characteristics | 1990 | | 2021 | | 1990–2021 |
| --- | --- | --- | --- | --- | --- |
|  | Number of deaths  cases (95% UI) | The age-standardized deaths  rate/100000 (95% UI) | Number of deaths  cases (95% UI) | The age-standardized deaths  rate/100000 (95% UI) | Number of deaths  cases (95% UI) |
| Northern Mariana Islands | 0 (0-0) | 0.01 (0-0.01) | 0 (0-0) | 0.01 (0-0.01) | 1.24 (0.81-1.68) |
| Norway | 0 (0-0) | 0 (0-0) | 0 (0-0) | 0 (0-0) | -3.14 (-3.48--2.8) |
| Oman | 0 (0-0) | 0 (0-0) | 0 (0-0) | 0 (0-0) | 0.21 (-0.24-0.65) |
| Pakistan | 4 (2-7) | 0.01 (0-0.01) | 18 (11-26) | 0.01 (0.01-0.01) | 1.68 (1.19-2.17) |
| Palau | 0 (0-0) | 0 (0-0) | 0 (0-0) | 0 (0-0) | 0.66 (0.37-0.95) |
| Palestine | 0 (0-0) | 0 (0-0) | 0 (0-0) | 0 (0-0) | -0.94 (-1.57--0.3) |
| Panama | 0 (0-0) | 0 (0-0) | 0 (0-0) | 0 (0-0) | 1.13 (0.87-1.38) |
| Papua New Guinea | 0 (0-0) | 0 (0-0) | 0 (0-0) | 0 (0-0) | 0.44 (-0.08-0.95) |
| Paraguay | 0 (0-0) | 0 (0-0) | 0 (0-0) | 0 (0-0) | 2.87 (2.48-3.26) |
| Peru | 0 (0-0) | 0 (0-0) | 0 (0-0) | 0 (0-0) | 0.24 (-0.04-0.52) |
| Philippines | 5 (3-7) | 0.01 (0.01-0.01) | 11 (7-16) | 0.01 (0.01-0.02) | 0.39 (0.11-0.68) |
| Poland | 0 (0-0) | 0 (0-0) | 0 (0-0) | 0 (0-0) | 0.13 (-0.28-0.54) |
| Portugal | 0 (0-0) | 0 (0-0) | 0 (0-0) | 0 (0-0) | -2.35 (-2.75--1.94) |
| Puerto Rico | 0 (0-0) | 0 (0-0) | 0 (0-0) | 0 (0-0) | -1.06 (-1.39--0.74) |
| Qatar | 0 (0-0) | 0 (0-0) | 0 (0-0) | 0 (0-0) | -1.69 (-2.14--1.24) |
| Republic of C么te d'Ivoire | 0 (0-0) | 0 (0-0) | 0 (0-1) | 0 (0-0) | 0.82 (0.23-1.41) |
| Republic of Korea | 0 (0-1) | 0 (0-0) | 0 (0-1) | 0 (0-0) | -1.84 (-2.21--1.46) |
| Republic of Moldova | 0 (0-0) | 0 (0-0) | 0 (0-0) | 0 (0-0) | -1.41 (-1.76--1.07) |

**Continued** **Supplementary Table 2.** Numbers, ASR, and Trends in Occupational Carcinogen-Attributable NPC Deaths across countries and territories: 1990–2021.

| Characteristics | 1990 | | 2021 | | 1990–2021 |
| --- | --- | --- | --- | --- | --- |
|  | Number of deaths  cases (95% UI) | The age-standardized deaths  rate/100000 (95% UI) | Number of deaths  cases (95% UI) | The age-standardized deaths  rate/100000 (95% UI) | Number of deaths  cases (95% UI) |
| Romania | 0 (0-0) | 0 (0-0) | 0 (0-0) | 0 (0-0) | 1.11 (0.65-1.58) |
| Russian Federation | 1 (1-1) | 0 (0-0) | 1 (0-1) | 0 (0-0) | -0.84 (-1.22--0.45) |
| Rwanda | 0 (0-1) | 0.01 (0.01-0.01) | 1 (0-1) | 0.01 (0-0.01) | -1.8 (-2.4--1.2) |
| Saint Kitts and Nevis | 0 (0-0) | 0 (0-0.01) | 0 (0-0) | 0 (0-0.01) | 0.01 (-0.37-0.39) |
| Saint Lucia | 0 (0-0) | 0 (0-0) | 0 (0-0) | 0 (0-0.01) | 0.79 (0.52-1.06) |
| Saint Vincent and the Grenadines | 0 (0-0) | 0 (0-0) | 0 (0-0) | 0 (0-0) | 2.11 (1.85-2.38) |
| Samoa | 0 (0-0) | 0.01 (0-0.01) | 0 (0-0) | 0.01 (0-0.01) | -0.45 (-0.85--0.04) |
| San Marino | 0 (0-0) | 0 (0-0) | 0 (0-0) | 0 (0-0) | -1.53 (-1.96--1.1) |
| Sao Tome and Principe | 0 (0-0) | 0 (0-0) | 0 (0-0) | 0 (0-0) | 0.87 (0.29-1.44) |
| Saudi Arabia | 1 (0-1) | 0.01 (0-0.01) | 2 (1-4) | 0.01 (0-0.01) | -0.83 (-1.19--0.46) |
| Senegal | 0 (0-0) | 0 (0-0) | 0 (0-0) | 0 (0-0) | 0.45 (-0.1-1.02) |
| Serbia | 0 (0-0) | 0 (0-0) | 0 (0-0) | 0 (0-0) | -0.1 (-0.47-0.27) |
| Seychelles | 0 (0-0) | 0.01 (0.01-0.02) | 0 (0-0) | 0.01 (0.01-0.01) | 0.6 (0.31-0.9) |
| Sierra Leone | 0 (0-0) | 0 (0-0) | 0 (0-0) | 0 (0-0) | 1.53 (1.01-2.06) |
| Singapore | 0 (0-1) | 0.01 (0.01-0.02) | 0 (0-0) | 0 (0-0) | -3.7 (-4--3.4) |
| Slovakia | 0 (0-0) | 0 (0-0) | 0 (0-0) | 0 (0-0) | -0.75 (-1.16--0.33) |
| Slovenia | 0 (0-0) | 0 (0-0) | 0 (0-0) | 0 (0-0) | -3.88 (-4.54--3.22) |
| Solomon Islands | 0 (0-0) | 0 (0-0.01) | 0 (0-0) | 0.01 (0-0.01) | 4.59 (3.91-5.27) |
| Somalia | 0 (0-0) | 0.01 (0-0.01) | 1 (0-1) | 0.01 (0-0.01) | -0.15 (-0.88-0.59) |

**Continued** **Supplementary Table 2.** Numbers, ASR, and Trends in Occupational Carcinogen-Attributable NPC Deaths across countries and territories: 1990–2021.

| Characteristics | 1990 | | 2021 | | 1990–2021 |
| --- | --- | --- | --- | --- | --- |
|  | Number of deaths  cases (95% UI) | The age-standardized deaths  rate/100000 (95% UI) | Number of deaths  cases (95% UI) | The age-standardized deaths  rate/100000 (95% UI) | Number of deaths  cases (95% UI) |
| South Africa | 1 (1-1) | 0 (0-0) | 1 (0-1) | 0 (0-0) | -2.76 (-3.03--2.49) |
| South Sudan | 0 (0-0) | 0.01 (0-0.01) | 0 (0-1) | 0.01 (0-0.01) | 0.48 (-0.25-1.22) |
| Spain | 0 (0-1) | 0 (0-0) | 0 (0-0) | 0 (0-0) | -3.35 (-3.78--2.93) |
| Sri Lanka | 1 (0-1) | 0 (0-0.01) | 1 (1-2) | 0 (0-0.01) | 0.58 (0.32-0.84) |
| Sudan | 0 (0-0) | 0 (0-0) | 0 (0-1) | 0 (0-0) | -0.94 (-1.52--0.36) |
| Suriname | 0 (0-0) | 0 (0-0) | 0 (0-0) | 0 (0-0.01) | 2.2 (1.95-2.45) |
| Sweden | 0 (0-0) | 0 (0-0) | 0 (0-0) | 0 (0-0) | -1.99 (-2.37--1.6) |
| Switzerland | 0 (0-0) | 0 (0-0) | 0 (0-0) | 0 (0-0) | -5.19 (-5.78--4.59) |
| Syrian Arab Republic | 0 (0-0) | 0 (0-0) | 0 (0-0) | 0 (0-0) | -2.26 (-2.88--1.64) |
| Taiwan (Province of China) | 10 (6-14) | 0.05 (0.03-0.07) | 8 (5-12) | 0.02 (0.01-0.03) | -2.26 (-2.61--1.91) |
| Tajikistan | 0 (0-0) | 0 (0-0) | 0 (0-0) | 0 (0-0) | -1.03 (-1.49--0.57) |
| Thailand | 5 (3-8) | 0.01 (0.01-0.02) | 10 (6-15) | 0.01 (0.01-0.02) | 0.29 (-0.07-0.64) |
| Timor-Leste | 0 (0-0) | 0.01 (0-0.01) | 0 (0-0) | 0.01 (0-0.01) | 0.41 (-0.14-0.96) |
| Togo | 0 (0-0) | 0 (0-0) | 0 (0-0) | 0 (0-0) | 1.7 (1.19-2.22) |
| Tokelau | 0 (0-0) | 0 (0-0.01) | 0 (0-0) | 0 (0-0.01) | -0.15 (-0.33-0.03) |
| Tonga | 0 (0-0) | 0 (0-0) | 0 (0-0) | 0 (0-0) | -0.05 (-0.49-0.4) |
| Trinidad and Tobago | 0 (0-0) | 0 (0-0) | 0 (0-0) | 0 (0-0) | 2.04 (1.69-2.39) |
| Tunisia | 1 (1-1) | 0.01 (0.01-0.02) | 2 (1-2) | 0.01 (0.01-0.02) | -0.05 (-0.27-0.16) |
| Turkey | 3 (2-4) | 0.01 (0-0.01) | 3 (2-4) | 0 (0-0) | -1.95 (-2.16--1.73) |

**Continued** **Supplementary Table 2.** Numbers, ASR, and Trends in Occupational Carcinogen-Attributable NPC Deaths across countries and territories: 1990–2021.

| Characteristics | 1990 | | 2021 | | 1990-2021 |
| --- | --- | --- | --- | --- | --- |
|  | Number of deaths  cases (95% UI) | The age-standardized deaths  rate/100000 (95% UI) | Number of deaths  cases (95% UI) | The age-standardized deaths  rate/100000 (95% UI) | Number of deaths  cases (95% UI) |
| Turkmenistan | 0 (0-0) | 0 (0-0) | 0 (0-0) | 0 (0-0) | 2.1 (1.63-2.58) |
| Tuvalu | 0 (0-0) | 0 (0-0.01) | 0 (0-0) | 0 (0-0.01) | -0.2 (-0.34--0.06) |
| Uganda | 1 (1-2) | 0.01 (0.01-0.02) | 4 (2-7) | 0.02 (0.01-0.03) | -0.49 (-1.36-0.4) |
| Ukraine | 0 (0-0) | 0 (0-0) | 0 (0-0) | 0 (0-0) | 1.17 (0.65-1.69) |
| United Arab Emirates | 0 (0-0) | 0.01 (0-0.01) | 0 (0-1) | 0 (0-0) | -1.28 (-1.47--1.1) |
| United Kingdom | 0 (0-0) | 0 (0-0) | 0 (0-0) | 0 (0-0) | -0.97 (-1.31--0.63) |
| United Republic of Tanzania | 1 (1-2) | 0.01 (0-0.01) | 3 (2-5) | 0.01 (0-0.01) | 0.52 (-0.11-1.15) |
| United States of America | 2 (1-2) | 0 (0-0) | 2 (1-2) | 0 (0-0) | -1.67 (-1.96--1.39) |
| United States Virgin Islands | 0 (0-0) | 0 (0-0) | 0 (0-0) | 0 (0-0) | 0.1 (-0.17-0.36) |
| Uruguay | 0 (0-0) | 0 (0-0.01) | 0 (0-0) | 0 (0-0) | -0.98 (-1.22--0.73) |
| Uzbekistan | 0 (0-0) | 0 (0-0) | 1 (1-2) | 0 (0-0.01) | 3.4 (3.09-3.72) |
| Vanuatu | 0 (0-0) | 0 (0-0.01) | 0 (0-0) | 0 (0-0.01) | 0.25 (-0.27-0.78) |
| Venezuela (Bolivarian Republic of) | 0 (0-0) | 0 (0-0) | 1 (0-1) | 0 (0-0) | 1.38 (1.03-1.73) |
| Viet Nam | 7 (4-10) | 0.01 (0.01-0.02) | 25 (15-38) | 0.02 (0.01-0.03) | 2.57 (2.28-2.87) |
| Yemen | 0 (0-0) | 0 (0-0) | 0 (0-0) | 0 (0-0) | -0.45 (-1.22-0.33) |
| Zambia | 0 (0-1) | 0.01 (0.01-0.01) | 2 (1-4) | 0.02 (0.01-0.03) | 1.9 (1.05-2.77) |
| Zimbabwe | 0 (0-0) | 0 (0-0) | 0 (0-1) | 0 (0-0.01) | 1.64 (0.9-2.39) |

**Supplementary Table 3.** Global Numbers, ASR, and Trends in Occupational Carcinogen-Attributable NPC DALYs: 1990–2021.

| Characteristics | 1990 | | 2021 | | 1990–2021 |
| --- | --- | --- | --- | --- | --- |
|  | Number of DALYs cases (95% UI) | The age-standardized DALYs rate/100000 (95% UI) | Number of DALYs cases (95% UI) | The age-standardized DALYs rate/100000 (95% UI) | EAPC (95% CI) |
| Global | 21692 (14222-29916) | 0.45 (0.3-0.62) | 25383 (16268-36800) | 0.3 (0.19-0.44) | -1.55 (-1.7--1.4) |
| **Sex** | | | | | |
| Female | 6354 (3584-9981) | 0.26 (0.15-0.41) | 6453 (3811-9876) | 0.15 (0.09-0.23) | -2.3 (-2.52--2.09) |
| Male | 15338 (8476-23322) | 0.64 (0.35-0.96) | 18930 (10841-29768) | 0.45 (0.26-0.7) | -1.63 (-1.83--1.44) |
| **Age** | | | | | |
| 15-19 years | 276 (63-627) | 0.05 (0.01-0.12) | 177 (40-401) | 0.03 (0.01-0.06) | -2.43 (-2.63--2.23) |
| 20-24 years | 634 (156-1352) | 0.13 (0.03-0.27) | 419 (105-893) | 0.07 (0.02-0.15) | -2.31 (-2.55--2.06) |
| 25-29 years | 2260 (594-4464) | 0.51 (0.13-1.01) | 1751 (422-3442) | 0.3 (0.07-0.59) | -2.31 (-2.6--2.03) |
| 30-34 years | 2385 (604-5024) | 0.62 (0.16-1.3) | 2444 (597-5309) | 0.4 (0.1-0.88) | -2.28 (-2.79--1.76) |
| 35-39 years | 4298 (993-9587) | 1.22 (0.28-2.72) | 3733 (869-7706) | 0.67 (0.15-1.37) | -2.37 (-2.59--2.14) |
| 40-44 years | 3887 (953-7711) | 1.36 (0.33-2.69) | 3886 (827-8146) | 0.78 (0.17-1.63) | -2.33 (-2.55--2.1) |
| 45-49 years | 3098 (616-6947) | 1.33 (0.27-2.99) | 4981 (990-11643) | 1.05 (0.21-2.46) | -1.03 (-1.37--0.69) |
| 50-54 years | 1788 (369-3988) | 0.84 (0.17-1.88) | 2748 (523-5985) | 0.62 (0.12-1.35) | -1.51 (-1.84--1.18) |
| 55-59 years | 1530 (333-3276) | 0.83 (0.18-1.77) | 2279 (487-4852) | 0.58 (0.12-1.23) | -1.88 (-2.15--1.62) |
| 60-64 years | 874 (168-1992) | 0.54 (0.1-1.24) | 1344 (251-3077) | 0.42 (0.08-0.96) | -0.91 (-1.13--0.7) |
| 65-69 years | 365 (79-814) | 0.3 (0.06-0.66) | 857 (188-2093) | 0.31 (0.07-0.76) | -0.82 (-1.18--0.47) |
| 70-74 years | 247 (60-549) | 0.29 (0.07-0.65) | 630 (151-1373) | 0.31 (0.07-0.67) | -0.43 (-0.65--0.2) |
| 75-79 years | 50 (12-106) | 0.08 (0.02-0.17) | 133 (34-280) | 0.1 (0.03-0.21) | 0.39 (0.18-0.59) |
| 80-84 years | 0 | 0 | 0 | 0 | * |

**Continued** **Supplementary Table 3.** Global Numbers, ASR, and Trends in Occupational Carcinogen-Attributable NPC DALYs: 1990–2021.

| Characteristics | 1990 | | 2021 | | 1990-2021 |
| --- | --- | --- | --- | --- | --- |
|  | Number of DALYs cases (95% UI) | The age-standardized DALYs rate/100000 (95% UI) | Number of DALYs cases (95% UI) | The age-standardized DALYs rate/100000 (95% UI) | Number of DALYs cases (95% UI) |
| 85-89 years | 0 | 0 | 0 | 0 | * |
| 90-94 years | 0 | 0 | 0 | 0 | * |
| 95+ years | 0 | 0 | 0 | 0 | * |
| **SDI regions** | | | | | |
| High-middle SDI | 7128 (4558-9992) | 0.66 (0.42-0.94) | 7392 (4618-11482) | 0.45 (0.29-0.69) | -2.11 (-2.41--1.8) |
| High SDI | 1226 (774-1666) | 0.12 (0.08-0.17) | 1031 (674-1505) | 0.08 (0.05-0.11) | -1.93 (-2.07--1.8) |
| Low-middle SDI | 2218 (1365-3223) | 0.25 (0.16-0.36) | 4603 (2939-6526) | 0.25 (0.16-0.36) | -0.05 (-0.1-0) |
| Low SDI | 740 (468-1078) | 0.22 (0.14-0.31) | 1654 (996-2515) | 0.2 (0.12-0.31) | -0.55 (-0.68--0.42) |
| Middle SDI | 10374 (6841-14430) | 0.7 (0.46-0.97) | 10693 (6905-15403) | 0.38 (0.25-0.54) | -2.41 (-2.63--2.19) |
| **GBD regions** | | | | | |
| Advanced Health System | 1079 (703-1507) | 0.07 (0.05-0.1) | 901 (596-1297) | 0.05 (0.03-0.07) | -1.53 (-1.77--1.29) |
| Africa | 844 (557-1178) | 0.19 (0.13-0.27) | 2102 (1308-3147) | 0.2 (0.12-0.29) | 0.05 (-0.39-0.49) |
| African Region | 685 (457-962) | 0.2 (0.13-0.28) | 1813 (1130-2738) | 0.21 (0.13-0.31) | 0.15 (-0.32-0.63) |
| America | 330 (223-444) | 0.05 (0.03-0.07) | 512 (352-679) | 0.04 (0.03-0.06) | -0.2 (-0.36--0.04) |
| Andean Latin America | 11 (8-15) | 0.04 (0.03-0.05) | 23 (15-31) | 0.03 (0.02-0.05) | 0.17 (-0.1-0.44) |
| Asia | 20103 (13139-27845) | 0.72 (0.48-1) | 22445 (14399-32524) | 0.43 (0.27-0.62) | -1.8 (-1.98--1.62) |
| Australasia | 8 (5-10) | 0.03 (0.02-0.05) | 7 (4-10) | 0.02 (0.01-0.03) | -1.99 (-2.22--1.76) |
| Basic Health System | 16970 (11239-23608) | 0.84 (0.55-1.15) | 17474 (11280-25919) | 0.47 (0.3-0.69) | -1.98 (-2.2--1.76) |

**Continued** **Supplementary Table 3.** Global Numbers, ASR, and Trends in Occupational Carcinogen-Attributable NPC DALYs: 1990–2021.

| Characteristics | 1990 | | 2021 | | 1990-2021 |
| --- | --- | --- | --- | --- | --- |
|  | Number of DALYs cases (95% UI) | The age-standardized DALYs rate/100000 (95% UI) | Number of DALYs cases (95% UI) | The age-standardized DALYs rate/100000 (95% UI) | Number of DALYs cases (95% UI) |
| Caribbean | 25 (17-33) | 0.08 (0.05-0.11) | 64 (43-91) | 0.12 (0.08-0.17) | 1.95 (1.83-2.08) |
| Central Africa | 36 (23-51) | 0.08 (0.05-0.11) | 95 (55-150) | 0.08 (0.05-0.12) | -0.07 (-0.61-0.47) |
| Central Asia | 48 (32-65) | 0.08 (0.05-0.11) | 107 (70-154) | 0.11 (0.07-0.15) | 1.46 (1.25-1.66) |
| Central Europe | 39 (25-55) | 0.03 (0.02-0.04) | 40 (26-58) | 0.03 (0.02-0.04) | -0.18 (-0.54-0.18) |
| Central Latin America | 75 (51-99) | 0.06 (0.04-0.08) | 139 (94-188) | 0.05 (0.03-0.07) | -0.24 (-0.45--0.02) |
| Central Sub-Saharan Africa | 26 (16-39) | 0.07 (0.04-0.11) | 61 (35-97) | 0.06 (0.04-0.1) | -0.42 (-0.96-0.13) |
| Commonwealth High Income | 50 (34-68) | 0.04 (0.03-0.06) | 41 (28-58) | 0.02 (0.02-0.03) | -1.8 (-2.05--1.54) |
| Commonwealth Low Income | 438 (260-671) | 0.32 (0.19-0.49) | 1130 (648-1880) | 0.35 (0.2-0.57) | 0.49 (0.04-0.93) |
| Commonwealth Middle Income | 3062 (1857-4346) | 0.33 (0.2-0.47) | 5362 (3392-7696) | 0.26 (0.17-0.38) | -0.69 (-0.92--0.46) |
| East Asia | 15144 (10059-21323) | 1.29 (0.86-1.8) | 13031 (8173-20337) | 0.69 (0.43-1.05) | -2.26 (-2.59--1.94) |
| East Asia & Pacific - WB | 17196 (11373-23901) | 0.97 (0.64-1.34) | 17354 (11227-25705) | 0.59 (0.38-0.88) | -1.78 (-2.04--1.52) |
| Eastern Africa | 372 (232-527) | 0.33 (0.21-0.47) | 1048 (644-1617) | 0.36 (0.23-0.55) | 0.27 (-0.28-0.82) |
| Eastern Europe | 53 (34-76) | 0.02 (0.01-0.03) | 48 (31-69) | 0.02 (0.01-0.03) | -0.62 (-0.97--0.27) |
| Eastern Mediterranean Region | 457 (278-654) | 0.17 (0.11-0.25) | 1366 (902-1947) | 0.19 (0.13-0.27) | 0.67 (0.36-0.99) |

**Continued** **Supplementary Table 3.** Global Numbers, ASR, and Trends in Occupational Carcinogen-Attributable NPC DALYs: 1990–2021.

| Characteristics | 1990 | | 2021 | | 1990-2021 |
| --- | --- | --- | --- | --- | --- |
|  | Number of DALYs cases (95% UI) | The age-standardized DALYs rate/100000 (95% UI) | Number of DALYs cases (95% UI) | The age-standardized DALYs rate/100000 (95% UI) | Number of DALYs cases (95% UI) |
| Eastern Sub-Saharan Africa | 403 (255-574) | 0.33 (0.21-0.47) | 1171 (708-1848) | 0.38 (0.23-0.59) | 0.38 (-0.19-0.95) |
| Europe | 408 (265-572) | 0.05 (0.03-0.06) | 312 (208-441) | 0.03 (0.02-0.04) | -1.47 (-1.71--1.23) |
| Europe & Central Asia - WB | 445 (289-624) | 0.05 (0.03-0.07) | 405 (276-569) | 0.04 (0.03-0.05) | -0.88 (-1.09--0.67) |
| European Region | 446 (290-625) | 0.05 (0.03-0.07) | 407 (278-571) | 0.04 (0.02-0.05) | -0.89 (-1.09--0.68) |
| High-income Asia Pacific | 68 (45-95) | 0.03 (0.02-0.05) | 55 (37-78) | 0.02 (0.01-0.03) | -1.5 (-1.9--1.1) |
| High-income North America | 82 (56-113) | 0.03 (0.02-0.04) | 68 (47-96) | 0.02 (0.01-0.02) | -1.89 (-2.13--1.64) |
| Latin America & Caribbean - WB | 249 (167-335) | 0.07 (0.05-0.09) | 445 (306-585) | 0.06 (0.04-0.08) | -0.06 (-0.28-0.16) |
| Limited Health System | 3557 (2212-5065) | 0.3 (0.19-0.43) | 6788 (4241-9637) | 0.26 (0.16-0.36) | -0.38 (-0.66--0.1) |
| Middle East & North Africa - WB | 291 (179-419) | 0.16 (0.1-0.23) | 617 (373-935) | 0.12 (0.08-0.18) | -0.2 (-0.44-0.05) |
| Minimal Health System | 79 (51-113) | 0.09 (0.06-0.13) | 208 (124-318) | 0.09 (0.05-0.14) | 0.09 (-0.49-0.68) |
| North Africa and Middle East | 444 (277-647) | 0.18 (0.11-0.26) | 781 (488-1156) | 0.12 (0.08-0.18) | -0.75 (-0.99--0.51) |
| North America | 82 (56-112) | 0.03 (0.02-0.04) | 68 (47-96) | 0.02 (0.01-0.02) | -1.88 (-2.12--1.63) |
| Northern Africa | 201 (122-292) | 0.22 (0.14-0.32) | 394 (236-604) | 0.19 (0.12-0.29) | 0.03 (-0.19-0.26) |
| Oceania | 5 (3-9) | 0.12 (0.07-0.19) | 14 (8-24) | 0.12 (0.07-0.2) | 0.48 (0.12-0.85) |

**Continued** **Supplementary Table 3.** Global Numbers, ASR, and Trends in Occupational Carcinogen-Attributable NPC DALYs: 1990–2021.

| Characteristics | 1990 | | 2021 | | 1990-2021 |
| --- | --- | --- | --- | --- | --- |
|  | Number of DALYs cases (95% UI) | The age-standardized DALYs rate/100000 (95% UI) | Number of DALYs cases (95% UI) | The age-standardized DALYs rate/100000 (95% UI) | Number of DALYs cases (95% UI) |
| Region of the Americas | 330 (223-444) | 0.05 (0.03-0.07) | 512 (352-679) | 0.04 (0.03-0.06) | -0.2 (-0.36--0.04) |
| South-East Asia Region | 3685 (2274-5232) | 0.35 (0.22-0.5) | 5993 (3807-8779) | 0.28 (0.18-0.4) | -0.55 (-0.71--0.39) |
| South Asia | 2744 (1659-4007) | 0.32 (0.19-0.46) | 4710 (2946-6878) | 0.25 (0.16-0.37) | -0.53 (-0.74--0.32) |
| South Asia - WB | 2779 (1682-4059) | 0.31 (0.19-0.45) | 4775 (3000-6959) | 0.25 (0.16-0.37) | -0.53 (-0.74--0.32) |
| Southeast Asia | 2004 (1322-2763) | 0.53 (0.35-0.73) | 4301 (2797-6115) | 0.55 (0.36-0.78) | 0.48 (0.34-0.63) |
| Southern Africa | 86 (57-120) | 0.13 (0.09-0.18) | 191 (93-324) | 0.12 (0.06-0.21) | 0.15 (-0.24-0.53) |
| Southern Latin America | 48 (32-70) | 0.1 (0.07-0.15) | 35 (23-51) | 0.05 (0.03-0.07) | -1.77 (-1.96--1.57) |
| Southern Sub-Saharan Africa | 47 (30-66) | 0.12 (0.08-0.16) | 56 (36-79) | 0.07 (0.05-0.1) | -1.36 (-1.66--1.06) |
| Sub-Saharan Africa - WB | 643 (424-907) | 0.19 (0.12-0.26) | 1706 (1046-2571) | 0.19 (0.12-0.29) | 0.07 (-0.43-0.56) |
| Tropical Latin America | 92 (57-128) | 0.07 (0.05-0.1) | 187 (126-255) | 0.07 (0.05-0.1) | 0.15 (-0.26-0.55) |
| Western Africa | 149 (92-217) | 0.13 (0.08-0.18) | 374 (205-594) | 0.12 (0.07-0.19) | -0.3 (-0.81-0.21) |
| Western Europe | 174 (114-251) | 0.04 (0.03-0.06) | 90 (62-126) | 0.02 (0.01-0.02) | -2.89 (-3.19--2.58) |
| Western Pacific Region | 15613 (10377-21917) | 1.05 (0.69-1.45) | 14953 (9461-22994) | 0.63 (0.4-0.94) | -1.93 (-2.22--1.63) |
| Western Sub-Saharan Africa | 153 (95-223) | 0.12 (0.07-0.17) | 395 (221-620) | 0.11 (0.06-0.18) | -0.23 (-0.74-0.29) |

**Supplementary Table 4.** Numbers, ASR, and Trends in Occupational Carcinogen-Attributable NPC DALYs across countries and territories: 1990–2021.

| Characteristics | 1990 | | 2021 | | 1990–2021 |
| --- | --- | --- | --- | --- | --- |
|  | Number of DALYs cases (95% UI) | The age-standardized DALYs rate/100000 (95% UI) | Number of DALYs cases (95% UI) | The age-standardized DALYs rate/100000 (95% UI) | EAPC (95% CI) |
| Afghanistan | 8 (3-12) | 0.11 (0.04-0.17) | 19 (9-32) | 0.09 (0.04-0.14) | -0.74 (-1.37--0.12) |
| Albania | 0 (0-1) | 0.01 (0.01-0.02) | 0 (0-1) | 0.02 (0.01-0.02) | 1.93 (1.62-2.24) |
| Algeria | 69 (41-109) | 0.39 (0.22-0.62) | 168 (96-264) | 0.36 (0.21-0.55) | 0.96 (0.67-1.26) |
| American Samoa | 0 (0-0) | 0.38 (0.22-0.58) | 0 (0-0) | 0.36 (0.21-0.55) | 0.91 (0.63-1.19) |
| Andorra | 0 (0-0) | 0.02 (0.01-0.03) | 0 (0-0) | 0.01 (0-0.02) | -1.49 (-1.92--1.06) |
| Angola | 6 (3-10) | 0.09 (0.05-0.14) | 14 (8-23) | 0.07 (0.04-0.1) | -0.87 (-1.44--0.29) |
| Antigua and Barbuda | 0 (0-0) | 0.06 (0.04-0.09) | 0 (0-0) | 0.06 (0.04-0.09) | 0.91 (0.72-1.1) |
| Argentina | 37 (24-55) | 0.12 (0.07-0.17) | 26 (17-38) | 0.05 (0.03-0.08) | -1.75 (-1.93--1.57) |
| Armenia | 1 (1-1) | 0.03 (0.02-0.04) | 2 (1-3) | 0.05 (0.03-0.08) | 2.77 (2.34-3.21) |
| Australia | 6 (4-9) | 0.04 (0.02-0.05) | 6 (4-9) | 0.02 (0.01-0.03) | -1.99 (-2.22--1.76) |
| Austria | 2 (1-3) | 0.03 (0.02-0.04) | 1 (1-2) | 0.01 (0.01-0.02) | -2.05 (-2.4--1.71) |
| Azerbaijan | 4 (2-6) | 0.06 (0.03-0.1) | 4 (2-6) | 0.03 (0.02-0.05) | -1.63 (-1.85--1.41) |
| Bahamas | 0 (0-0) | 0.09 (0.06-0.12) | 1 (0-1) | 0.13 (0.08-0.19) | 1.46 (1.22-1.69) |
| Bahrain | 1 (0-1) | 0.18 (0.11-0.28) | 2 (1-3) | 0.1 (0.06-0.17) | -1.68 (-1.94--1.42) |
| Bangladesh | 238 (128-386) | 0.33 (0.17-0.53) | 473 (227-903) | 0.29 (0.14-0.55) | 0.23 (-0.12-0.59) |
| Barbados | 0 (0-0) | 0.1 (0.07-0.14) | 0 (0-1) | 0.12 (0.08-0.18) | 1.13 (0.82-1.44) |
| Belarus | 3 (2-4) | 0.03 (0.01-0.04) | 2 (1-3) | 0.02 (0.01-0.03) | -1.19 (-1.58--0.79) |
| Belgium | 3 (2-5) | 0.03 (0.02-0.04) | 3 (2-4) | 0.02 (0.01-0.03) | -1.89 (-2.23--1.56) |
| Belize | 0 (0-0) | 0.04 (0.03-0.05) | 0 (0-0) | 0.08 (0.06-0.12) | 3.02 (2.48-3.56) |
| Benin | 1 (1-2) | 0.04 (0.02-0.06) | 6 (3-9) | 0.06 (0.03-0.1) | 1.32 (0.73-1.92) |

**Continued** **Supplementary Table 4.** Numbers, ASR, and Trends in Occupational Carcinogen-Attributable NPC DALYs across countries and territories: 1990–2021.

| Characteristics | 1990 | | 2021 | | 1990–2021 |
| --- | --- | --- | --- | --- | --- |
|  | Number of DALYs cases (95% UI) | The age-standardized DALYs rate/100000 (95% UI) | Number of DALYs cases (95% UI) | The age-standardized DALYs rate/100000 (95% UI) | Number of DALYs cases (95% UI) |
| Bermuda | 0 (0-0) | 0.11 (0.07-0.17) | 0 (0-0) | 0.09 (0.05-0.13) | -0.26 (-0.66-0.13) |
| Bhutan | 1 (1-2) | 0.27 (0.15-0.43) | 2 (1-3) | 0.23 (0.12-0.44) | -0.01 (-0.31-0.3) |
| Bolivia (Plurinational State of) | 2 (1-3) | 0.05 (0.03-0.07) | 6 (4-10) | 0.06 (0.03-0.09) | 0.26 (-0.1-0.62) |
| Bosnia and Herzegovina | 0 (0-0) | 0 (0-0.01) | 0 (0-0) | 0.01 (0-0.01) | 2.33 (1.96-2.69) |
| Botswana | 1 (0-1) | 0.1 (0.05-0.16) | 2 (1-3) | 0.07 (0.03-0.12) | -0.33 (-0.76-0.09) |
| Brazil | 91 (57-127) | 0.07 (0.05-0.1) | 183 (123-250) | 0.07 (0.05-0.1) | 0.11 (-0.3-0.52) |
| Brunei Darussalam | 0 (0-1) | 0.19 (0.12-0.28) | 1 (0-1) | 0.13 (0.09-0.19) | -0.02 (-0.21-0.17) |
| Bulgaria | 2 (1-3) | 0.02 (0.01-0.03) | 2 (2-4) | 0.03 (0.02-0.04) | 1.18 (0.81-1.56) |
| Burkina Faso | 2 (1-3) | 0.03 (0.02-0.05) | 8 (4-13) | 0.05 (0.03-0.08) | 1.58 (0.96-2.2) |
| Burundi | 13 (8-19) | 0.35 (0.21-0.52) | 28 (15-46) | 0.3 (0.16-0.5) | -0.75 (-1.3--0.19) |
| Cabo Verde | 0 (0-0) | 0.01 (0.01-0.02) | 0 (0-1) | 0.06 (0.03-0.1) | 5.66 (4.36-6.98) |
| Cambodia | 33 (19-50) | 0.47 (0.29-0.71) | 109 (66-167) | 0.66 (0.4-1.02) | 1.86 (1.52-2.2) |
| Cameroon | 2 (1-4) | 0.03 (0.02-0.05) | 16 (9-26) | 0.07 (0.04-0.11) | 2.69 (2.16-3.21) |
| Canada | 9 (6-13) | 0.03 (0.02-0.04) | 7 (4-10) | 0.01 (0.01-0.02) | -2.71 (-3.02--2.4) |
| Central African Republic | 2 (1-3) | 0.09 (0.05-0.15) | 3 (2-5) | 0.07 (0.04-0.12) | -0.83 (-1.33--0.33) |
| Chad | 1 (0-1) | 0.02 (0.01-0.03) | 4 (2-7) | 0.04 (0.02-0.06) | 2.43 (1.71-3.14) |
| Chile | 6 (4-9) | 0.05 (0.03-0.07) | 5 (3-7) | 0.02 (0.01-0.03) | -1.89 (-2.16--1.63) |

**Continued** **Supplementary Table 4.** Numbers, ASR, and Trends in Occupational Carcinogen-Attributable NPC DALYs across countries and territories: 1990–2021.

| Characteristics | 1990 | | 2021 | | 1990–2021 |
| --- | --- | --- | --- | --- | --- |
|  | Number of DALYs cases (95% UI) | The age-standardized DALYs rate/100000 (95% UI) | Number of DALYs cases (95% UI) | The age-standardized DALYs rate/100000 (95% UI) | Number of DALYs cases (95% UI) |
| China | 14556 (9652-20573) | 1.29 (0.86-1.8) | 12512 (7766-19708) | 0.68 (0.43-1.05) | -2.28 (-2.61--1.96) |
| Colombia | 23 (15-31) | 0.08 (0.06-0.11) | 27 (17-40) | 0.05 (0.03-0.07) | -1.57 (-1.72--1.43) |
| Comoros | 1 (0-1) | 0.22 (0.1-0.37) | 2 (1-3) | 0.26 (0.13-0.49) | 0.78 (0.34-1.22) |
| Congo | 1 (1-2) | 0.09 (0.06-0.14) | 4 (2-6) | 0.07 (0.04-0.12) | -0.76 (-1.18--0.33) |
| Cook Islands | 0 (0-0) | 0.04 (0.02-0.06) | 0 (0-0) | 0.04 (0.02-0.06) | 1.31 (1.04-1.58) |
| Costa Rica | 3 (2-4) | 0.13 (0.08-0.18) | 5 (3-7) | 0.09 (0.06-0.13) | -1.59 (-1.89--1.28) |
| Croatia | 1 (1-2) | 0.02 (0.01-0.03) | 1 (1-1) | 0.02 (0.01-0.02) | -0.15 (-0.7-0.4) |
| Cuba | 9 (6-13) | 0.08 (0.05-0.12) | 20 (13-32) | 0.13 (0.08-0.19) | 1.94 (1.62-2.27) |
| Cyprus | 0 (0-0) | 0.01 (0.01-0.02) | 0 (0-0) | 0.01 (0-0.01) | -1.78 (-2.06--1.5) |
| Czechia | 5 (3-7) | 0.04 (0.03-0.06) | 3 (1-4) | 0.02 (0.01-0.03) | -2.39 (-2.73--2.05) |
| Democratic People's Republic of Korea | 120 (70-192) | 0.57 (0.34-0.92) | 194 (116-314) | 0.59 (0.36-0.94) | 0.38 (0.19-0.57) |
| Democratic Republic of the Congo | 16 (9-25) | 0.06 (0.04-0.1) | 39 (21-64) | 0.06 (0.03-0.1) | -0.2 (-0.76-0.36) |
| Denmark | 1 (1-1) | 0.02 (0.01-0.02) | 1 (0-1) | 0.01 (0-0.01) | -2.71 (-3.06--2.35) |
| Djibouti | 1 (0-1) | 0.18 (0.09-0.34) | 3 (1-5) | 0.23 (0.1-0.44) | 1.13 (0.82-1.44) |
| Dominica | 0 (0-0) | 0.05 (0.04-0.08) | 0 (0-0) | 0.09 (0.06-0.14) | 2.73 (2.5-2.96) |
| Dominican Republic | 6 (4-8) | 0.11 (0.07-0.15) | 13 (8-21) | 0.12 (0.07-0.19) | 1.1 (0.87-1.33) |
| Ecuador | 3 (2-4) | 0.03 (0.02-0.05) | 6 (3-8) | 0.03 (0.02-0.05) | 0.31 (-0.16-0.78) |
| Egypt | 9 (5-13) | 0.02 (0.01-0.03) | 14 (8-22) | 0.02 (0.01-0.02) | -0.73 (-1.03--0.44) |

**Continued** **Supplementary Table 4.** Numbers, ASR, and Trends in Occupational Carcinogen-Attributable NPC DALYs across countries and territories: 1990–2021.

| Characteristics | 1990 | | 2021 | | 1990–2021 |
| --- | --- | --- | --- | --- | --- |
|  | Number of DALYs cases (95% UI) | The age-standardized DALYs rate/100000 (95% UI) | Number of DALYs cases (95% UI) | The age-standardized DALYs rate/100000 (95% UI) | Number of DALYs cases (95% UI) |
| El Salvador | 2 (1-3) | 0.05 (0.03-0.07) | 4 (3-6) | 0.07 (0.04-0.1) | 1.59 (1.3-1.88) |
| Equatorial Guinea | 0 (0-0) | 0.05 (0.03-0.09) | 1 (0-1) | 0.06 (0.03-0.11) | 0.55 (-0.06-1.16) |
| Eritrea | 8 (5-12) | 0.35 (0.22-0.53) | 22 (12-37) | 0.41 (0.22-0.67) | 1 (0.53-1.46) |
| Estonia | 1 (1-2) | 0.06 (0.04-0.09) | 0 (0-0) | 0.01 (0.01-0.02) | -4.8 (-5.07--4.52) |
| Eswatini | 0 (0-1) | 0.1 (0.06-0.15) | 1 (1-2) | 0.1 (0.05-0.16) | 0.85 (0.2-1.51) |
| Ethiopia | 111 (62-167) | 0.33 (0.19-0.49) | 235 (128-404) | 0.29 (0.17-0.49) | -0.7 (-1.31--0.09) |
| Fiji | 0 (0-0) | 0.05 (0.03-0.08) | 0 (0-1) | 0.05 (0.03-0.07) | 0.16 (-0.05-0.38) |
| Finland | 1 (1-1) | 0.01 (0.01-0.02) | 0 (0-1) | 0.01 (0-0.01) | -2.73 (-3.08--2.37) |
| France | 48 (29-74) | 0.07 (0.05-0.11) | 18 (12-28) | 0.02 (0.01-0.03) | -3.94 (-4.21--3.67) |
| Gabon | 0 (0-1) | 0.07 (0.04-0.11) | 1 (0-1) | 0.05 (0.03-0.09) | -0.78 (-1.17--0.39) |
| Gambia | 0 (0-0) | 0.03 (0.02-0.05) | 1 (0-1) | 0.04 (0.03-0.07) | 1.17 (0.61-1.72) |
| Georgia | 5 (3-7) | 0.08 (0.05-0.12) | 4 (2-6) | 0.1 (0.06-0.15) | 0.98 (0.59-1.37) |
| Germany | 35 (21-53) | 0.04 (0.02-0.05) | 15 (10-23) | 0.01 (0.01-0.02) | -2.65 (-3.08--2.21) |
| Ghana | 5 (3-9) | 0.05 (0.02-0.08) | 1 (1-2) | 0 (0-0.01) | -10.62 (-11.79--9.42) |
| Greece | 3 (2-4) | 0.03 (0.02-0.04) | 2 (2-4) | 0.02 (0.01-0.03) | -1.59 (-1.94--1.24) |
| Greenland | 0 (0-1) | 0.74 (0.48-1.06) | 0 (0-0) | 0.23 (0.14-0.35) | -3.43 (-3.7--3.15) |
| Grenada | 0 (0-0) | 0.11 (0.07-0.15) | 0 (0-0) | 0.14 (0.09-0.21) | 1.94 (1.65-2.23) |
| Guam | 0 (0-1) | 0.4 (0.24-0.61) | 1 (0-1) | 0.28 (0.15-0.46) | -0.24 (-0.49-0.02) |
| Guatemala | 5 (3-6) | 0.09 (0.06-0.11) | 9 (6-12) | 0.06 (0.04-0.09) | -0.94 (-1.42--0.46) |
| Guinea | 2 (1-2) | 0.04 (0.02-0.06) | 6 (3-10) | 0.06 (0.04-0.11) | 1.63 (1.09-2.17) |

**Continued** **Supplementary Table 4.** Numbers, ASR, and Trends in Occupational Carcinogen-Attributable NPC DALYs across countries and territories: 1990–2021.

| Characteristics | 1990 | | 2021 | | 1990–2021 |
| --- | --- | --- | --- | --- | --- |
|  | Number of DALYs cases (95% UI) | The age-standardized DALYs rate/100000 (95% UI) | Number of DALYs cases (95% UI) | The age-standardized DALYs rate/100000 (95% UI) | Number of DALYs cases (95% UI) |
| Guinea-Bissau | 0 (0-0) | 0.05 (0.03-0.07) | 1 (1-2) | 0.07 (0.04-0.12) | 1.61 (1.07-2.16) |
| Guyana | 0 (0-0) | 0.04 (0.03-0.06) | 1 (0-1) | 0.08 (0.05-0.13) | 2.75 (2.41-3.09) |
| Haiti | 5 (2-7) | 0.1 (0.05-0.15) | 18 (9-28) | 0.16 (0.09-0.25) | 2.54 (2.16-2.92) |
| Honduras | 2 (1-2) | 0.05 (0.03-0.07) | 5 (3-8) | 0.06 (0.04-0.09) | 0.56 (0.09-1.03) |
| Hungary | 5 (3-7) | 0.04 (0.03-0.06) | 5 (3-7) | 0.04 (0.02-0.05) | -0.64 (-1.03--0.25) |
| Iceland | 0 (0-0) | 0.03 (0.02-0.04) | 0 (0-0) | 0.02 (0.01-0.02) | -1.89 (-2.16--1.62) |
| India | 2260 (1333-3264) | 0.32 (0.2-0.47) | 3336 (2031-4967) | 0.23 (0.14-0.34) | -1 (-1.19--0.82) |
| Indonesia | 656 (406-989) | 0.43 (0.27-0.63) | 1263 (711-2050) | 0.4 (0.23-0.64) | 0.16 (0.02-0.3) |
| Iran (Islamic Republic of) | 13 (8-19) | 0.03 (0.02-0.05) | 26 (16-36) | 0.03 (0.02-0.03) | 0.07 (-0.32-0.45) |
| Iraq | 12 (6-19) | 0.1 (0.05-0.15) | 26 (13-43) | 0.07 (0.04-0.11) | -0.93 (-1.35--0.5) |
| Ireland | 1 (0-1) | 0.02 (0.01-0.03) | 1 (0-1) | 0.01 (0.01-0.02) | -1.82 (-2.06--1.58) |
| Israel | 1 (1-1) | 0.02 (0.01-0.03) | 1 (1-2) | 0.01 (0.01-0.02) | -1.42 (-1.64--1.2) |
| Italy | 27 (18-39) | 0.04 (0.03-0.06) | 13 (8-19) | 0.02 (0.01-0.02) | -2.96 (-3.3--2.61) |
| Jamaica | 1 (1-2) | 0.06 (0.04-0.08) | 3 (2-5) | 0.1 (0.06-0.17) | 1.95 (1.49-2.42) |
| Japan | 33 (22-47) | 0.02 (0.01-0.03) | 32 (21-45) | 0.02 (0.01-0.02) | -0.97 (-1.46--0.47) |
| Jordan | 4 (2-6) | 0.17 (0.1-0.27) | 11 (6-19) | 0.09 (0.05-0.15) | -2 (-2.4--1.6) |
| Kazakhstan | 16 (11-22) | 0.1 (0.07-0.14) | 22 (14-31) | 0.11 (0.07-0.15) | 0.24 (0.05-0.42) |
| Kenya | 51 (28-81) | 0.36 (0.2-0.59) | 243 (137-381) | 0.61 (0.35-0.96) | 2.38 (1.83-2.94) |
| Kiribati | 0 (0-0) | 0.07 (0.04-0.11) | 0 (0-0) | 0.12 (0.06-0.21) | 3.12 (2.67-3.57) |

**Continued** **Supplementary Table 4.** Numbers, ASR, and Trends in Occupational Carcinogen-Attributable NPC DALYs across countries and territories: 1990–2021.

| Characteristics | 1990 | | 2021 | | 1990–2021 |
| --- | --- | --- | --- | --- | --- |
|  | Number of DALYs cases (95% UI) | The age-standardized DALYs rate/100000 (95% UI) | Number of DALYs cases (95% UI) | The age-standardized DALYs rate/100000 (95% UI) | Number of DALYs cases (95% UI) |
| Kuwait | 2 (1-3) | 0.14 (0.09-0.21) | 3 (2-5) | 0.05 (0.03-0.08) | -2.14 (-2.5--1.78) |
| Kyrgyzstan | 4 (2-6) | 0.12 (0.07-0.19) | 8 (5-12) | 0.12 (0.07-0.18) | 0.92 (0.65-1.19) |
| Lao People's Democratic Republic | 17 (9-25) | 0.56 (0.3-0.86) | 33 (19-52) | 0.47 (0.28-0.73) | -0.34 (-0.73-0.06) |
| Latvia | 1 (1-2) | 0.05 (0.03-0.07) | 0 (0-1) | 0.01 (0.01-0.02) | -4.73 (-5.1--4.36) |
| Lebanon | 3 (2-5) | 0.12 (0.06-0.21) | 5 (3-7) | 0.08 (0.04-0.12) | -0.9 (-1.04--0.76) |
| Lesotho | 1 (0-1) | 0.07 (0.04-0.12) | 2 (1-3) | 0.12 (0.07-0.19) | 2.93 (2.35-3.51) |
| Liberia | 1 (0-1) | 0.03 (0.02-0.06) | 3 (1-5) | 0.06 (0.03-0.1) | 2.24 (1.77-2.71) |
| Libya | 17 (9-27) | 0.59 (0.33-0.97) | 42 (24-71) | 0.5 (0.29-0.81) | 0.43 (0.09-0.77) |
| Lithuania | 2 (1-3) | 0.04 (0.02-0.06) | 1 (0-1) | 0.01 (0.01-0.02) | -3.23 (-3.58--2.87) |
| Luxembourg | 0 (0-0) | 0.04 (0.03-0.06) | 0 (0-0) | 0.01 (0.01-0.02) | -4.17 (-4.51--3.83) |
| Madagascar | 26 (16-38) | 0.32 (0.2-0.47) | 72 (40-118) | 0.33 (0.18-0.52) | 0.22 (-0.24-0.7) |
| Malawi | 9 (6-15) | 0.14 (0.09-0.22) | 18 (10-29) | 0.13 (0.07-0.22) | -0.62 (-1.21--0.03) |
| Malaysia | 369 (227-543) | 2.55 (1.59-3.74) | 662 (402-969) | 1.93 (1.17-2.86) | -1.29 (-1.66--0.92) |
| Maldives | 0 (0-0) | 0.12 (0.06-0.2) | 0 (0-0) | 0.05 (0.03-0.07) | -2.5 (-2.91--2.09) |
| Mali | 2 (1-3) | 0.03 (0.02-0.04) | 5 (3-8) | 0.03 (0.02-0.05) | 0.45 (-0.16-1.06) |
| Malta | 0 (0-0) | 0.08 (0.05-0.11) | 0 (0-0) | 0.05 (0.03-0.07) | -1.44 (-1.75--1.13) |
| Marshall Islands | 0 (0-0) | 0.15 (0.09-0.24) | 0 (0-0) | 0.15 (0.08-0.25) | 0.88 (0.42-1.34) |
| Mauritania | 0 (0-1) | 0.02 (0.01-0.04) | 1 (0-2) | 0.03 (0.01-0.05) | 0.8 (0.34-1.27) |
| Mauritius | 2 (1-3) | 0.23 (0.14-0.32) | 4 (2-5) | 0.22 (0.14-0.32) | 0.08 (-0.19-0.35) |

**Continued** **Supplementary Table 4.** Numbers, ASR, and Trends in Occupational Carcinogen-Attributable NPC DALYs across countries and territories: 1990–2021.

| Characteristics | 1990 | | 2021 | | 1990–2021 |
| --- | --- | --- | --- | --- | --- |
|  | Number of DALYs cases (95% UI) | The age-standardized DALYs rate/100000 (95% UI) | Number of DALYs cases (95% UI) | The age-standardized DALYs rate/100000 (95% UI) | Number of DALYs cases (95% UI) |
| Mexico | 31 (20-41) | 0.05 (0.03-0.07) | 58 (39-80) | 0.04 (0.03-0.06) | -0.16 (-0.39-0.08) |
| Micronesia (Federated States of) | 0 (0-0) | 0.23 (0.12-0.37) | 0 (0-0) | 0.19 (0.1-0.31) | 0.1 (-0.27-0.48) |
| Monaco | 0 (0-0) | 0.02 (0.01-0.03) | 0 (0-0) | 0.01 (0.01-0.02) | -0.87 (-1.18--0.55) |
| Mongolia | 1 (1-2) | 0.07 (0.04-0.12) | 3 (2-6) | 0.1 (0.05-0.16) | 1.67 (1.42-1.93) |
| Montenegro | 0 (0-0) | 0 (0-0.01) | 0 (0-0) | 0 (0-0.01) | 0.28 (0.03-0.53) |
| Morocco | 68 (39-105) | 0.36 (0.21-0.55) | 100 (54-173) | 0.25 (0.14-0.43) | -0.76 (-0.97--0.56) |
| Mozambique | 1 (1-2) | 0.01 (0.01-0.02) | 4 (2-6) | 0.02 (0.01-0.03) | 2.43 (1.74-3.12) |
| Myanmar | 88 (54-134) | 0.27 (0.16-0.41) | 187 (113-293) | 0.32 (0.2-0.51) | 0.89 (0.63-1.15) |
| Namibia | 1 (1-1) | 0.1 (0.06-0.16) | 2 (1-3) | 0.08 (0.04-0.12) | -0.58 (-1--0.17) |
| Nauru | 0 (0-0) | 0.24 (0.12-0.42) | 0 (0-0) | 0.22 (0.11-0.41) | -0.1 (-0.49-0.3) |
| Nepal | 44 (25-67) | 0.3 (0.18-0.46) | 60 (35-96) | 0.21 (0.12-0.33) | -0.71 (-1.04--0.39) |
| Netherlands | 4 (3-6) | 0.02 (0.02-0.03) | 4 (2-5) | 0.02 (0.01-0.02) | -1.28 (-1.67--0.88) |
| New Zealand | 1 (1-2) | 0.03 (0.02-0.04) | 1 (1-1) | 0.01 (0.01-0.02) | -2 (-2.25--1.76) |
| Nicaragua | 1 (1-1) | 0.03 (0.02-0.05) | 3 (2-5) | 0.05 (0.03-0.07) | 2.26 (1.79-2.73) |
| Niger | 1 (1-2) | 0.03 (0.02-0.05) | 6 (3-11) | 0.04 (0.02-0.07) | 0.59 (-0.08-1.27) |
| Nigeria | 126 (75-183) | 0.2 (0.12-0.29) | 306 (165-499) | 0.19 (0.1-0.3) | -0.4 (-0.9-0.11) |
| Niue | 0 (0-0) | 0.16 (0.09-0.25) | 0 (0-0) | 0.14 (0.08-0.22) | -0.19 (-0.35--0.03) |
| North Macedonia | 0 (0-0) | 0.01 (0.01-0.02) | 0 (0-1) | 0.01 (0.01-0.02) | 1.2 (0.9-1.52) |

**Continued** **Supplementary Table 4.** Numbers, ASR, and Trends in Occupational Carcinogen-Attributable NPC DALYs across countries and territories: 1990–2021.

| Characteristics | 1990 | | 2021 | | 1990–2021 |
| --- | --- | --- | --- | --- | --- |
|  | Number of DALYs cases (95% UI) | The age-standardized DALYs rate/100000 (95% UI) | Number of DALYs cases (95% UI) | The age-standardized DALYs rate/100000 (95% UI) | Number of DALYs cases (95% UI) |
| Northern Mariana Islands | 0 (0-0) | 0.31 (0.18-0.53) | 0 (0-0) | 0.33 (0.2-0.52) | 0.88 (0.41-1.34) |
| Norway | 1 (0-1) | 0.01 (0.01-0.02) | 0 (0-0) | 0 (0-0.01) | -3.26 (-3.54--2.99) |
| Oman | 1 (1-2) | 0.07 (0.04-0.12) | 3 (1-5) | 0.05 (0.03-0.09) | 0.29 (-0.04-0.61) |
| Pakistan | 201 (113-315) | 0.26 (0.15-0.42) | 839 (535-1214) | 0.41 (0.26-0.6) | 1.73 (1.31-2.15) |
| Palau | 0 (0-0) | 0.01 (0-0.02) | 0 (0-0) | 0.01 (0-0.01) | 0.52 (0.22-0.82) |
| Palestine | 1 (0-1) | 0.05 (0.03-0.08) | 1 (1-2) | 0.03 (0.02-0.05) | -0.99 (-1.51--0.47) |
| Panama | 1 (1-1) | 0.05 (0.04-0.07) | 3 (2-4) | 0.07 (0.04-0.1) | 1.02 (0.8-1.25) |
| Papua New Guinea | 3 (1-5) | 0.1 (0.05-0.18) | 9 (4-17) | 0.11 (0.05-0.19) | 0.43 (0.01-0.85) |
| Paraguay | 1 (1-1) | 0.03 (0.02-0.05) | 4 (3-7) | 0.06 (0.04-0.1) | 2.7 (2.36-3.05) |
| Peru | 6 (4-8) | 0.03 (0.02-0.05) | 11 (6-16) | 0.03 (0.02-0.04) | 0 (-0.22-0.22) |
| Philippines | 241 (157-345) | 0.49 (0.31-0.68) | 505 (335-720) | 0.46 (0.31-0.66) | 0.16 (-0.06-0.38) |
| Poland | 10 (6-14) | 0.02 (0.02-0.03) | 11 (7-16) | 0.02 (0.01-0.03) | -0.14 (-0.51-0.23) |
| Portugal | 6 (4-9) | 0.05 (0.03-0.08) | 4 (2-6) | 0.02 (0.02-0.04) | -2.65 (-2.99--2.3) |
| Puerto Rico | 1 (0-1) | 0.02 (0.01-0.03) | 1 (0-1) | 0.01 (0.01-0.02) | -1.28 (-1.54--1.01) |
| Qatar | 0 (0-1) | 0.09 (0.05-0.15) | 2 (1-3) | 0.05 (0.03-0.09) | -1.61 (-2.1--1.12) |
| Republic of d'Ivoire | 5 (3-8) | 0.07 (0.04-0.1) | 19 (10-31) | 0.09 (0.04-0.15) | 0.86 (0.38-1.35) |
| Republic of Korea | 18 (11-27) | 0.04 (0.03-0.06) | 13 (8-19) | 0.02 (0.01-0.03) | -2.18 (-2.53--1.83) |
| Republic of Moldova | 1 (1-2) | 0.03 (0.02-0.05) | 1 (1-1) | 0.02 (0.01-0.03) | -1.61 (-1.92--1.3) |
| Romania | 9 (5-13) | 0.04 (0.02-0.05) | 12 (7-19) | 0.05 (0.03-0.08) | 0.79 (0.38-1.21) |

**Continued** **Supplementary Table 4.** Numbers, ASR, and Trends in Occupational Carcinogen-Attributable NPC DALYs across countries and territories: 1990–2021.

| Characteristics | 1990 | | 2021 | | 1990–2021 |
| --- | --- | --- | --- | --- | --- |
|  | Number of DALYs cases (95% UI) | The age-standardized DALYs rate/100000 (95% UI) | Number of DALYs cases (95% UI) | The age-standardized DALYs rate/100000 (95% UI) | Number of DALYs cases (95% UI) |
| Russian Federation | 37 (24-53) | 0.02 (0.01-0.03) | 32 (20-46) | 0.02 (0.01-0.02) | -0.86 (-1.21--0.5) |
| Rwanda | 20 (12-30) | 0.42 (0.26-0.63) | 28 (15-47) | 0.26 (0.14-0.44) | -1.91 (-2.41--1.4) |
| Saint Kitts and Nevis | 0 (0-0) | 0.2 (0.14-0.28) | 0 (0-0) | 0.13 (0.08-0.19) | -0.48 (-0.88--0.09) |
| Saint Lucia | 0 (0-0) | 0.16 (0.11-0.21) | 0 (0-0) | 0.15 (0.1-0.22) | 0.66 (0.42-0.89) |
| Saint Vincent and the Grenadines | 0 (0-0) | 0.09 (0.06-0.13) | 0 (0-0) | 0.14 (0.09-0.2) | 1.89 (1.65-2.12) |
| Samoa | 0 (0-1) | 0.31 (0.19-0.47) | 0 (0-1) | 0.27 (0.16-0.45) | -0.47 (-0.83--0.1) |
| San Marino | 0 (0-0) | 0.06 (0.04-0.09) | 0 (0-0) | 0.03 (0.01-0.05) | -1.64 (-2--1.27) |
| Sao Tome and Principe | 0 (0-0) | 0 (0-0.01) | 0 (0-0) | 0.01 (0-0.01) | 0.86 (0.32-1.4) |
| Saudi Arabia | 40 (20-67) | 0.35 (0.19-0.57) | 111 (60-193) | 0.22 (0.13-0.37) | -0.75 (-1.07--0.43) |
| Senegal | 2 (1-3) | 0.04 (0.02-0.06) | 5 (2-9) | 0.04 (0.02-0.07) | 0.52 (0.03-1.02) |
| Serbia | 2 (1-4) | 0.02 (0.01-0.04) | 2 (1-3) | 0.02 (0.01-0.03) | -0.39 (-0.69--0.09) |
| Seychelles | 0 (0-0) | 0.45 (0.27-0.67) | 1 (0-1) | 0.42 (0.26-0.65) | 0.51 (0.21-0.81) |
| Sierra Leone | 1 (0-1) | 0.02 (0.01-0.04) | 2 (1-4) | 0.04 (0.02-0.06) | 1.66 (1.21-2.12) |
| Singapore | 17 (10-24) | 0.5 (0.32-0.71) | 10 (6-15) | 0.12 (0.08-0.18) | -4.14 (-4.47--3.81) |
| Slovakia | 3 (2-5) | 0.06 (0.03-0.09) | 2 (1-4) | 0.03 (0.02-0.06) | -0.96 (-1.35--0.58) |
| Slovenia | 1 (0-1) | 0.03 (0.02-0.05) | 0 (0-0) | 0.01 (0.01-0.02) | -4.13 (-4.76--3.5) |
| Solomon Islands | 0 (0-0) | 0.12 (0.06-0.21) | 2 (1-3) | 0.3 (0.16-0.51) | 4.67 (4.05-5.29) |
| Somalia | 15 (8-24) | 0.28 (0.15-0.45) | 39 (20-68) | 0.29 (0.15-0.49) | -0.13 (-0.76-0.49) |

**Continued** **Supplementary Table 4.** Numbers, ASR, and Trends in Occupational Carcinogen-Attributable NPC DALYs across countries and territories: 1990–2021.

| Characteristics | 1990 | | 2021 | | 1990–2021 |
| --- | --- | --- | --- | --- | --- |
|  | Number of DALYs cases (95% UI) | The age-standardized DALYs rate/100000 (95% UI) | Number of DALYs cases (95% UI) | The age-standardized DALYs rate/100000 (95% UI) | Number of DALYs cases (95% UI) |
| South Africa | 37 (24-51) | 0.12 (0.08-0.17) | 30 (19-43) | 0.05 (0.03-0.07) | -2.79 (-3.03--2.55) |
| South Sudan | 8 (4-14) | 0.22 (0.12-0.38) | 17 (9-29) | 0.25 (0.13-0.44) | 0.48 (-0.13-1.1) |
| Spain | 20 (13-29) | 0.05 (0.03-0.07) | 10 (7-15) | 0.02 (0.01-0.02) | -3.62 (-4--3.24) |
| Sri Lanka | 28 (17-41) | 0.17 (0.11-0.26) | 46 (25-75) | 0.18 (0.1-0.3) | 0.24 (-0.02-0.5) |
| Sudan | 12 (6-21) | 0.09 (0.04-0.15) | 22 (12-37) | 0.06 (0.03-0.11) | -0.89 (-1.37--0.42) |
| Suriname | 0 (0-1) | 0.12 (0.08-0.16) | 1 (1-2) | 0.17 (0.11-0.26) | 2.04 (1.81-2.27) |
| Sweden | 1 (1-2) | 0.01 (0.01-0.02) | 1 (0-1) | 0.01 (0-0.01) | -2.11 (-2.38--1.83) |
| Switzerland | 5 (3-7) | 0.06 (0.04-0.08) | 1 (1-2) | 0.01 (0.01-0.02) | -5.55 (-6.09--5) |
| Syrian Arab Republic | 3 (2-5) | 0.04 (0.02-0.06) | 3 (2-5) | 0.02 (0.01-0.03) | -2.47 (-2.99--1.95) |
| Taiwan (Province of China) | 468 (293-664) | 2.31 (1.44-3.27) | 325 (204-489) | 0.98 (0.6-1.47) | -2.52 (-2.85--2.19) |
| Tajikistan | 3 (2-6) | 0.09 (0.05-0.14) | 6 (4-10) | 0.07 (0.04-0.1) | -1 (-1.39--0.61) |
| Thailand | 250 (155-364) | 0.47 (0.3-0.69) | 428 (246-650) | 0.5 (0.3-0.75) | 0.12 (-0.25-0.49) |
| Timor-Leste | 2 (1-3) | 0.26 (0.14-0.46) | 3 (2-5) | 0.31 (0.17-0.5) | 0.17 (-0.33-0.67) |
| Togo | 1 (1-2) | 0.06 (0.03-0.09) | 6 (3-9) | 0.08 (0.05-0.14) | 1.67 (1.24-2.11) |
| Tokelau | 0 (0-0) | 0.18 (0.09-0.3) | 0 (0-0) | 0.16 (0.09-0.26) | -0.09 (-0.29-0.11) |
| Tonga | 0 (0-0) | 0.11 (0.07-0.19) | 0 (0-0) | 0.11 (0.06-0.19) | -0.01 (-0.43-0.41) |
| Trinidad and Tobago | 1 (0-1) | 0.07 (0.05-0.09) | 2 (1-3) | 0.11 (0.07-0.16) | 1.98 (1.63-2.32) |
| Tunisia | 38 (23-56) | 0.58 (0.36-0.86) | 68 (38-107) | 0.49 (0.27-0.76) | -0.09 (-0.28-0.11) |

**Continued** **Supplementary Table 4.** Numbers, ASR, and Trends in Occupational Carcinogen-Attributable NPC DALYs across countries and territories: 1990–2021.

| Characteristics | 1990 | | 2021 | | 1990–2021 |
| --- | --- | --- | --- | --- | --- |
|  | Number of DALYs cases (95% UI) | The age-standardized DALYs rate/100000 (95% UI) | Number of DALYs cases (95% UI) | The age-standardized DALYs rate/100000 (95% UI) | Number of DALYs cases (95% UI) |
| Turkey | 134 (81-211) | 0.28 (0.16-0.43) | 126 (76-194) | 0.13 (0.08-0.2) | -2.14 (-2.34--1.95) |
| Turkmenistan | 2 (1-3) | 0.07 (0.05-0.1) | 6 (3-9) | 0.11 (0.07-0.16) | 2.08 (1.65-2.52) |
| Tuvalu | 0 (0-0) | 0.2 (0.12-0.33) | 0 (0-0) | 0.18 (0.11-0.3) | -0.24 (-0.37--0.11) |
| Uganda | 62 (37-94) | 0.61 (0.37-0.94) | 200 (113-323) | 0.71 (0.42-1.16) | -0.46 (-1.22-0.31) |
| Ukraine | 8 (5-13) | 0.01 (0.01-0.02) | 12 (6-19) | 0.02 (0.01-0.04) | 1.17 (0.69-1.66) |
| United Arab Emirates | 4 (2-8) | 0.22 (0.11-0.41) | 16 (8-26) | 0.11 (0.07-0.17) | -1.37 (-1.66--1.08) |
| United Kingdom | 14 (10-19) | 0.02 (0.02-0.03) | 13 (9-18) | 0.02 (0.01-0.02) | -0.97 (-1.22--0.72) |
| United Republic of Tanzania | 56 (33-87) | 0.34 (0.2-0.54) | 161 (87-258) | 0.37 (0.21-0.6) | 0.5 (-0.04-1.03) |
| United States of America | 72 (49-99) | 0.03 (0.02-0.04) | 61 (42-86) | 0.02 (0.01-0.02) | -1.78 (-2.01--1.55) |
| United States Virgin Islands | 0 (0-0) | 0.15 (0.1-0.21) | 0 (0-0) | 0.13 (0.07-0.2) | 0.2 (0.02-0.38) |
| Uruguay | 5 (3-8) | 0.15 (0.1-0.23) | 4 (2-6) | 0.1 (0.06-0.15) | -0.94 (-1.16--0.72) |
| Uzbekistan | 12 (7-19) | 0.08 (0.04-0.11) | 53 (32-84) | 0.15 (0.09-0.23) | 3.36 (3.1-3.61) |
| Vanuatu | 0 (0-0) | 0.16 (0.09-0.26) | 0 (0-1) | 0.16 (0.09-0.26) | 0.22 (-0.24-0.67) |
| Venezuela (Bolivarian Republic of) | 8 (6-11) | 0.06 (0.04-0.08) | 24 (14-37) | 0.08 (0.05-0.12) | 1.38 (1.09-1.66) |
| Viet Nam | 317 (204-465) | 0.64 (0.41-0.93) | 1055 (632-1640) | 0.91 (0.54-1.4) | 2.41 (2.15-2.66) |

**Continued** **Supplementary Table 4.** Numbers, ASR, and Trends in Occupational Carcinogen-Attributable NPC DALYs across countries and territories: 1990–2021.

| Characteristics | 1990 | | 2021 | | 1990–2021 |
| --- | --- | --- | --- | --- | --- |
|  | Number of DALYs cases (95% UI) | The age-standardized DALYs rate/100000 (95% UI) | Number of DALYs cases (95% UI) | The age-standardized DALYs rate/100000 (95% UI) | Number of DALYs cases (95% UI) |
| Yemen | 4 (2-8) | 0.06 (0.03-0.1) | 12 (6-20) | 0.05 (0.03-0.08) | -0.38 (-1.05-0.3) |
| Zambia | 23 (14-33) | 0.46 (0.29-0.67) | 98 (32-203) | 0.7 (0.23-1.45) | 1.87 (1.11-2.63) |
| Zimbabwe | 7 (4-10) | 0.11 (0.07-0.17) | 20 (11-31) | 0.15 (0.09-0.24) | 2.05 (1.36-2.74) |

**Supplementary Table 5.** Global Numbers, ASR, and Trends in Occupational Carcinogen-Attributable LC Deaths: 1990–2021.

| Characteristics | 1990 | | 2021 | | 1990–2021 |
| --- | --- | --- | --- | --- | --- |
|  | Number of deaths  cases (95% UI) | The age-standardized deaths  rate/100000 (95% UI) | Number of deaths  cases (95% UI) | The age-standardized deaths  rate/100000 (95% UI) | EAPC (95% CI) |
| Global | 5369 (3296-7772) | 0.14 (0.08-0.19) | 6946 (4351-10142) | 0.08 (0.05-0.12) | -1.15 (-1.4--0.9) |
| **Sex** | | | | | |
| Female | 366 (198-585) | 0.02 (0.01-0.03) | 600 (314-976) | 0.01 (0.01-0.02) | -1.05 (-1.1--0.99) |
| Male | 5003 (3038-7143) | 0.29 (0.17-0.4) | 6345 (3922-9237) | 0.16 (0.1-0.23) | -1.93 (-1.99--1.87) |
| **Age** | | | | | |
| 20-24 years | 0 (0-0) | 0 (0-0) | 0 (0-0) | 0 (0-0) | -4.12 (-5.11--3.11) |
| 25-29 years | 6 (3-12) | 0 (0-0) | 6 (2-11) | 0 (0-0) | -1.31 (-1.38--1.23) |
| 30-34 years | 12 (5-23) | 0 (0-0.01) | 13 (5-24) | 0 (0-0) | -1.46 (-1.53--1.38) |
| 35-39 years | 40 (16-71) | 0.01 (0-0.02) | 38 (15-69) | 0.01 (0-0.01) | -1.86 (-1.93--1.8) |
| 40-44 years | 136 (58-240) | 0.05 (0.02-0.08) | 132 (57-239) | 0.03 (0.01-0.05) | -2.4 (-2.54--2.26) |
| 45-49 years | 245 (111-428) | 0.11 (0.05-0.18) | 279 (131-485) | 0.06 (0.03-0.1) | -2.08 (-2.18--1.98) |
| 50-54 years | 546 (270-923) | 0.26 (0.13-0.43) | 593 (291-1010) | 0.13 (0.07-0.23) | -2.28 (-2.35--2.21) |
| 55-59 years | 738 (413-1172) | 0.4 (0.22-0.63) | 830 (449-1373) | 0.21 (0.11-0.35) | -2.25 (-2.41--2.08) |
| 60-64 years | 955 (554-1483) | 0.59 (0.34-0.92) | 1027 (574-1654) | 0.32 (0.18-0.52) | -2.06 (-2.16--1.97) |
| 65-69 years | 824 (501-1179) | 0.67 (0.4-0.95) | 1060 (648-1628) | 0.38 (0.24-0.59) | -2.02 (-2.11--1.92) |
| 70-74 years | 636 (389-882) | 0.75 (0.46-1.04) | 996 (631-1409) | 0.48 (0.31-0.68) | -1.71 (-1.84--1.59) |
| 75-79 years | 597 (370-851) | 0.97 (0.6-1.38) | 749 (454-1061) | 0.57 (0.34-0.8) | -1.57 (-1.63--1.51) |
| 80-84 years | 402 (241-581) | 1.14 (0.68-1.64) | 615 (361-904) | 0.7 (0.41-1.03) | -1.34 (-1.48--1.2) |
| 85-89 years | 177 (99-256) | 1.17 (0.66-1.69) | 394 (222-577) | 0.86 (0.49-1.26) | -0.72 (-0.85--0.58) |
| 90-94 years | 46 (26-67) | 1.07 (0.62-1.57) | 170 (93-249) | 0.95 (0.52-1.39) | -0.15 (-0.31-0.02) |
| 95+ years | 9 (5-13) | 0.85 (0.45-1.27) | 44 (23-67) | 0.81 (0.42-1.23) | -0.13 (-0.23--0.02) |

**Continued Supplementary Table 5.** Global Numbers, ASR, and Trends in Occupational Carcinogen-Attributable LC Deaths: 1990–2021.

| Characteristics | 1990 | | 2021 | | 1990–2021 |
| --- | --- | --- | --- | --- | --- |
|  | Number of deaths  cases (95% UI) | The age-standardized deaths  rate/100000 (95% UI) | Number of deaths  cases (95% UI) | The age-standardized deaths  rate/100000 (95% UI) | Number of deaths  cases (95% UI) |
| **SDI regions** | | | | | |
| High-middle SDI | 1696 (1006-2444) | 0.17 (0.1-0.24) | 1567 (972-2278) | 0.08 (0.05-0.11) | -2.64 (-2.72--2.56) |
| High SDI | 1850 (1107-2566) | 0.16 (0.1-0.23) | 1816 (1070-2515) | 0.08 (0.05-0.11) | -2.26 (-2.37--2.16) |
| Low-middle SDI | 646 (332-1110) | 0.1 (0.05-0.17) | 1393 (780-2306) | 0.09 (0.05-0.15) | -0.17 (-0.21--0.13) |
| Low SDI | 196 (94-349) | 0.08 (0.04-0.14) | 363 (191-633) | 0.07 (0.04-0.12) | -0.66 (-0.75--0.58) |
| Middle SDI | 974 (523-1647) | 0.09 (0.05-0.15) | 1799 (1004-2873) | 0.07 (0.04-0.1) | -1.22 (-1.29--1.15) |
| **GBD regions** | | | | | |
| Advanced Health System | 3132 (1831-4388) | 0.19 (0.11-0.27) | 2768 (1643-3842) | 0.09 (0.05-0.13) | -1.68 (-2.28--1.08) |
| Africa | 178 (96-300) | 0.06 (0.03-0.1) | 335 (184-568) | 0.05 (0.03-0.08) | -0.77 (-1.82-0.28) |
| African Region | 145 (77-245) | 0.06 (0.03-0.1) | 270 (150-450) | 0.05 (0.03-0.08) | -0.92 (-2.05-0.22) |
| America | 894 (558-1255) | 0.15 (0.09-0.21) | 1191 (734-1688) | 0.09 (0.05-0.12) | -1.12 (-1.31--0.93) |
| Andean Latin America | 14 (8-21) | 0.07 (0.04-0.11) | 22 (13-38) | 0.04 (0.02-0.06) | -1.36 (-1.96--0.74) |
| Asia | 1825 (979-3089) | 0.09 (0.05-0.14) | 3400 (1875-5479) | 0.07 (0.04-0.1) | -0.14 (-0.46-0.19) |
| Australasia | 56 (33-77) | 0.23 (0.14-0.32) | 52 (31-74) | 0.09 (0.05-0.13) | -2.55 (-3.09--2) |
| Basic Health System | 1265 (684-2152) | 0.08 (0.05-0.14) | 2190 (1208-3557) | 0.06 (0.03-0.09) | -0.25 (-0.58-0.07) |
| Caribbean | 34 (19-54) | 0.13 (0.08-0.21) | 73 (41-123) | 0.13 (0.07-0.23) | 1.01 (0.76-1.25) |
| Central Africa | 15 (7-28) | 0.05 (0.02-0.09) | 32 (15-56) | 0.04 (0.02-0.08) | -0.55 (-1.68-0.6) |
| Central Asia | 69 (38-115) | 0.14 (0.07-0.22) | 50 (28-78) | 0.06 (0.03-0.09) | -2.71 (-3.08--2.35) |
| Central Europe | 214 (102-380) | 0.14 (0.07-0.25) | 281 (158-440) | 0.13 (0.07-0.21) | 0.66 (0.07-1.26) |

**Continued Supplementary Table 5.** Global Numbers, ASR, and Trends in Occupational Carcinogen-Attributable LC Deaths: 1990–2021.

| Characteristics | 1990 | | 2021 | | 1990–2021 |
| --- | --- | --- | --- | --- | --- |
|  | Number of deaths  cases (95% UI) | The age-standardized deaths  rate/100000 (95% UI) | Number of deaths  cases (95% UI) | The age-standardized deaths  rate/100000 (95% UI) | Number of deaths  cases (95% UI) |
| Central Latin America | 83 (49-131) | 0.1 (0.06-0.16) | 129 (76-206) | 0.05 (0.03-0.08) | -1.38 (-1.97--0.79) |
| Central Sub-Saharan Africa | 13 (6-25) | 0.05 (0.02-0.1) | 25 (12-46) | 0.04 (0.02-0.08) | -1.03 (-2.18-0.13) |
| Commonwealth High Income | 340 (208-465) | 0.22 (0.13-0.3) | 314 (193-427) | 0.11 (0.07-0.14) | -1.84 (-2.5--1.18) |
| Commonwealth Low Income | 100 (50-168) | 0.11 (0.05-0.18) | 198 (98-350) | 0.08 (0.04-0.14) | -0.33 (-1.3-0.66) |
| Commonwealth Middle Income | 800 (415-1363) | 0.12 (0.06-0.2) | 1660 (915-2712) | 0.1 (0.06-0.16) | -0.18 (-0.78-0.42) |
| East Asia | 632 (307-1116) | 0.07 (0.04-0.12) | 1056 (518-1841) | 0.05 (0.02-0.08) | -0.17 (-0.47-0.14) |
| East Asia & Pacific - WB | 919 (506-1541) | 0.07 (0.04-0.11) | 1591 (858-2612) | 0.05 (0.03-0.08) | -0.13 (-0.41-0.15) |
| Eastern Africa | 47 (20-88) | 0.06 (0.03-0.11) | 88 (41-166) | 0.05 (0.02-0.09) | -0.87 (-2.03-0.31) |
| Eastern Europe | 404 (200-700) | 0.14 (0.07-0.24) | 235 (129-375) | 0.07 (0.04-0.11) | -2.66 (-3.19--2.13) |
| Eastern Mediterranean Region | 174 (89-303) | 0.09 (0.05-0.15) | 410 (208-708) | 0.08 (0.04-0.14) | -0.1 (-0.94-0.74) |
| Eastern Sub-Saharan Africa | 50 (21-91) | 0.06 (0.03-0.11) | 100 (45-186) | 0.05 (0.02-0.1) | -0.61 (-1.79-0.59) |
| Europe | 2462 (1454-3502) | 0.23 (0.14-0.34) | 2008 (1194-2791) | 0.12 (0.07-0.17) | -1.48 (-2.1--0.86) |
| Europe & Central Asia - WB | 2500 (1488-3553) | 0.23 (0.14-0.33) | 2030 (1216-2820) | 0.12 (0.07-0.17) | -1.54 (-2.11--0.98) |

**Continued Supplementary Table 5.** Global Numbers, ASR, and Trends in Occupational Carcinogen-Attributable LC Deaths: 1990–2021.

| Characteristics | 1990 | | 2021 | | 1990–2021 |
| --- | --- | --- | --- | --- | --- |
|  | Number of deaths  cases (95% UI) | The age-standardized deaths  rate/100000 (95% UI) | Number of deaths  cases (95% UI) | The age-standardized deaths  rate/100000 (95% UI) | Number of deaths  cases (95% UI) |
| European Region | 2507 (1492-3564) | 0.23 (0.14-0.33) | 2040 (1221-2835) | 0.12 (0.07-0.17) | -1.55 (-2.11--0.99) |
| High-income Asia Pacific | 103 (55-160) | 0.05 (0.03-0.08) | 160 (89-230) | 0.03 (0.02-0.04) | -0.37 (-1.31-0.58) |
| High-income North America | 514 (298-722) | 0.14 (0.08-0.2) | 537 (309-759) | 0.08 (0.04-0.11) | -1.76 (-2.32--1.21) |
| Latin America & Caribbean - WB | 384 (232-599) | 0.14 (0.09-0.22) | 656 (412-1012) | 0.09 (0.06-0.14) | -0.4 (-0.8-0) |
| Limited Health System | 932 (477-1603) | 0.11 (0.06-0.18) | 1914 (1052-3165) | 0.09 (0.05-0.15) | -0.2 (-0.88-0.49) |
| Middle East & North Africa - WB | 75 (39-127) | 0.06 (0.03-0.1) | 150 (81-250) | 0.04 (0.02-0.07) | -0.41 (-1.16-0.35) |
| Minimal Health System | 34 (16-61) | 0.05 (0.02-0.09) | 66 (31-119) | 0.05 (0.02-0.08) | -0.59 (-1.72-0.57) |
| North Africa and Middle East | 225 (124-369) | 0.14 (0.08-0.22) | 314 (186-504) | 0.07 (0.04-0.11) | -1.73 (-2.52--0.94) |
| North America | 514 (298-722) | 0.14 (0.08-0.2) | 537 (309-759) | 0.08 (0.04-0.11) | -1.76 (-2.32--1.21) |
| Northern Africa | 29 (13-52) | 0.04 (0.02-0.08) | 60 (28-105) | 0.04 (0.02-0.06) | 0.16 (-0.46-0.78) |
| Oceania | 0 (0-1) | 0.01 (0.01-0.02) | 1 (1-2) | 0.01 (0.01-0.02) | 0.22 (-0.76-1.2) |
| Region of the Americas | 894 (558-1255) | 0.15 (0.09-0.21) | 1191 (734-1688) | 0.09 (0.05-0.12) | -1.12 (-1.31--0.93) |
| South-East Asia Region | 812 (413-1405) | 0.1 (0.05-0.18) | 1642 (885-2686) | 0.09 (0.05-0.14) | -0.01 (-0.5-0.49) |
| South Asia | 809 (412-1388) | 0.13 (0.07-0.21) | 1675 (917-2756) | 0.11 (0.06-0.18) | -0.08 (-0.64-0.49) |

**Continued Supplementary Table 5.** Global Numbers, ASR, and Trends in Occupational Carcinogen-Attributable LC Deaths: 1990–2021.

| Characteristics | 1990 | | 2021 | | 1990–2021 |
| --- | --- | --- | --- | --- | --- |
|  | Number of deaths  cases (95% UI) | The age-standardized deaths  rate/100000 (95% UI) | Number of deaths  cases (95% UI) | The age-standardized deaths  rate/100000 (95% UI) | Number of deaths  cases (95% UI) |
| South Asia - WB | 820 (417-1404) | 0.13 (0.07-0.21) | 1694 (929-2788) | 0.11 (0.06-0.18) | -0.07 (-0.64-0.49) |
| Southeast Asia | 131 (67-218) | 0.05 (0.03-0.08) | 335 (174-578) | 0.05 (0.03-0.08) | 0.82 (0.33-1.32) |
| Southern Africa | 51 (29-80) | 0.12 (0.07-0.18) | 94 (56-141) | 0.1 (0.06-0.15) | -0.67 (-1.76-0.43) |
| Southern Latin America | 94 (57-147) | 0.2 (0.12-0.31) | 92 (56-131) | 0.1 (0.06-0.15) | -1.19 (-1.41--0.98) |
| Southern Sub-Saharan Africa | 41 (24-63) | 0.15 (0.09-0.24) | 69 (41-104) | 0.13 (0.07-0.19) | -0.54 (-1.42-0.35) |
| Sub-Saharan Africa - WB | 149 (79-253) | 0.06 (0.04-0.11) | 276 (152-464) | 0.05 (0.03-0.09) | -0.93 (-2.09-0.23) |
| Tropical Latin America | 161 (98-249) | 0.17 (0.11-0.26) | 343 (213-519) | 0.13 (0.08-0.2) | 0.29 (-0.09-0.68) |
| Western Africa | 36 (19-64) | 0.04 (0.02-0.07) | 61 (31-105) | 0.03 (0.02-0.05) | -1.13 (-2.22--0.03) |
| Western Europe | 1684 (1003-2322) | 0.29 (0.17-0.4) | 1327 (795-1857) | 0.13 (0.08-0.18) | -1.67 (-2.51--0.83) |
| Western Pacific Region | 817 (454-1362) | 0.07 (0.04-0.11) | 1368 (743-2262) | 0.05 (0.03-0.08) | -0.19 (-0.48-0.11) |
| Western Sub-Saharan Africa | 38 (21-68) | 0.04 (0.02-0.07) | 71 (36-121) | 0.03 (0.02-0.06) | -0.92 (-2.01-0.19) |

**Supplementary Table 6.** Numbers, ASR, and Trends in Occupational Carcinogen-Attributable LC Deaths across countries and territories 1990–2021.

| Characteristics | 1990 | | 2021 | | 1990–2021 |
| --- | --- | --- | --- | --- | --- |
|  | Number of deaths  cases (95% UI) | The age-standardized deaths  rate/100000 (95% UI) | Number of deaths  cases (95% UI) | The age-standardized deaths  rate/100000 (95% UI) | EAPC (95% CI) |
| Afghanistan | 7 (3-14) | 0.09 (0.04-0.18) | 7 (3-15) | 0.06 (0.03-0.13) | -2.51 (-3.62--1.38) |
| Albania | 2 (1-3) | 0.09 (0.05-0.16) | 3 (1-6) | 0.07 (0.03-0.13) | 1.12 (0.66-1.57) |
| Algeria | 5 (2-8) | 0.04 (0.02-0.07) | 10 (4-17) | 0.03 (0.01-0.05) | 0.18 (-0.59-0.96) |
| American Samoa | 0 (0-0) | 0.05 (0.03-0.08) | 0 (0-0) | 0.01 (0.01-0.02) | -3.94 (-4.72--3.14) |
| Andorra | 0 (0-0) | 0.26 (0.13-0.46) | 0 (0-0) | 0.12 (0.05-0.2) | -1.65 (-2.16--1.14) |
| Angola | 3 (1-6) | 0.06 (0.03-0.12) | 5 (2-11) | 0.04 (0.02-0.09) | -1.46 (-2.72--0.19) |
| Antigua and Barbuda | 0 (0-0) | 0.08 (0.04-0.13) | 0 (0-0) | 0.06 (0.03-0.11) | -0.09 (-0.47-0.3) |
| Argentina | 73 (43-115) | 0.22 (0.13-0.35) | 72 (44-103) | 0.13 (0.08-0.18) | -0.95 (-1.17--0.72) |
| Armenia | 8 (5-12) | 0.27 (0.17-0.4) | 6 (3-8) | 0.13 (0.08-0.19) | -1.65 (-1.99--1.31) |
| Australia | 50 (30-69) | 0.25 (0.15-0.34) | 46 (28-66) | 0.09 (0.06-0.13) | -2.59 (-3.15--2.04) |
| Austria | 18 (10-27) | 0.15 (0.08-0.23) | 15 (8-22) | 0.08 (0.04-0.11) | -1 (-1.78--0.22) |
| Azerbaijan | 9 (4-16) | 0.16 (0.08-0.28) | 7 (3-13) | 0.06 (0.03-0.11) | -2.93 (-3.24--2.61) |
| Bahamas | 0 (0-0) | 0.13 (0.08-0.19) | 0 (0-1) | 0.12 (0.06-0.2) | 0.58 (0.15-1.02) |
| Bahrain | 0 (0-0) | 0.15 (0.09-0.24) | 1 (0-1) | 0.12 (0.06-0.2) | -0.49 (-2.17-1.21) |
| Bangladesh | 74 (37-128) | 0.15 (0.08-0.26) | 140 (68-251) | 0.1 (0.05-0.18) | -0.25 (-1.07-0.57) |
| Barbados | 0 (0-0) | 0.07 (0.04-0.12) | 0 (0-1) | 0.08 (0.04-0.13) | 0.89 (0.43-1.37) |
| Belarus | 19 (10-35) | 0.14 (0.07-0.26) | 15 (7-27) | 0.1 (0.05-0.17) | -1.93 (-2.5--1.36) |
| Belgium | 53 (31-78) | 0.34 (0.2-0.48) | 36 (21-51) | 0.15 (0.09-0.21) | -2.03 (-2.84--1.2) |
| Belize | 0 (0-0) | 0.09 (0.06-0.13) | 0 (0-0) | 0.11 (0.07-0.17) | 0.85 (-0.14-1.86) |
| Benin | 1 (0-1) | 0.04 (0.02-0.06) | 2 (1-4) | 0.04 (0.02-0.07) | 0.14 (-1.1-1.4) |

**Continued Supplementary Table 6.** Numbers, ASR, and Trends in Occupational Carcinogen-Attributable LC Deaths across countries and territories 1990–2021.

| Characteristics | 1990 | | 2021 | | 1990–2021 |
| --- | --- | --- | --- | --- | --- |
|  | Number of deaths  cases (95% UI) | The age-standardized deaths  rate/100000 (95% UI) | Number of deaths  cases (95% UI) | The age-standardized deaths  rate/100000 (95% UI) | Number of deaths  cases (95% UI) |
| Bermuda | 0 (0-0) | 0.37 (0.23-0.52) | 0 (0-0) | 0.13 (0.07-0.2) | -1.84 (-2.48--1.2) |
| Bhutan | 0 (0-0) | 0.09 (0.04-0.17) | 1 (0-1) | 0.08 (0.04-0.15) | 0.76 (0.03-1.5) |
| Bolivia (Plurinational State of) | 3 (1-4) | 0.08 (0.05-0.14) | 7 (4-12) | 0.08 (0.04-0.14) | 0.59 (-0.17-1.36) |
| Bosnia and Herzegovina | 5 (3-9) | 0.13 (0.07-0.21) | 6 (3-11) | 0.1 (0.05-0.18) | -0.01 (-0.58-0.57) |
| Botswana | 1 (0-2) | 0.17 (0.09-0.28) | 1 (1-2) | 0.1 (0.05-0.17) | -2.48 (-3.66--1.29) |
| Brazil | 160 (97-247) | 0.18 (0.11-0.27) | 337 (209-508) | 0.13 (0.08-0.2) | 0.26 (-0.13-0.64) |
| Brunei Darussalam | 0 (0-0) | 0.12 (0.06-0.19) | 0 (0-0) | 0.05 (0.03-0.07) | -1.78 (-3.12--0.43) |
| Bulgaria | 9 (3-20) | 0.07 (0.02-0.15) | 11 (4-24) | 0.09 (0.03-0.19) | 1.33 (0.62-2.05) |
| Burkina Faso | 2 (1-3) | 0.04 (0.02-0.07) | 4 (2-7) | 0.04 (0.02-0.07) | -0.02 (-1.12-1.09) |
| Burundi | 2 (1-4) | 0.08 (0.03-0.16) | 3 (1-6) | 0.06 (0.03-0.12) | -1.66 (-2.83--0.48) |
| Cabo Verde | 0 (0-0) | 0.03 (0.01-0.05) | 0 (0-0) | 0.03 (0.01-0.05) | -0.23 (-1.01-0.54) |
| Cambodia | 3 (1-6) | 0.07 (0.03-0.12) | 10 (4-19) | 0.08 (0.03-0.14) | 1.28 (0.44-2.12) |
| Cameroon | 2 (1-3) | 0.04 (0.02-0.06) | 6 (3-12) | 0.05 (0.02-0.09) | 0.91 (-0.25-2.08) |
| Canada | 67 (40-96) | 0.2 (0.12-0.29) | 65 (41-92) | 0.08 (0.05-0.11) | -1.83 (-2.47--1.17) |
| Central African Republic | 1 (0-2) | 0.07 (0.03-0.14) | 1 (1-3) | 0.05 (0.02-0.12) | -1.14 (-2.26--0.01) |
| Chad | 1 (0-1) | 0.02 (0.01-0.04) | 3 (1-5) | 0.04 (0.02-0.08) | 1.4 (0.14-2.68) |
| Chile | 11 (6-16) | 0.11 (0.06-0.16) | 10 (6-16) | 0.04 (0.02-0.06) | -1.93 (-2.21--1.66) |

**Continued Supplementary Table 6.** Numbers, ASR, and Trends in Occupational Carcinogen-Attributable LC Deaths across countries and territories 1990–2021.

| Characteristics | 1990 | | 2021 | | 1990–2021 |
| --- | --- | --- | --- | --- | --- |
|  | Number of deaths  cases (95% UI) | The age-standardized deaths  rate/100000 (95% UI) | Number of deaths  cases (95% UI) | The age-standardized deaths  rate/100000 (95% UI) | Number of deaths  cases (95% UI) |
| China | 616 (299-1086) | 0.07 (0.04-0.12) | 1027 (501-1800) | 0.05 (0.02-0.08) | -0.17 (-0.48-0.14) |
| Colombia | 18 (10-30) | 0.1 (0.06-0.17) | 25 (14-42) | 0.05 (0.03-0.08) | -1.88 (-2.36--1.4) |
| Comoros | 0 (0-0) | 0.05 (0.02-0.09) | 0 (0-0) | 0.04 (0.02-0.08) | -0.23 (-1.07-0.61) |
| Congo | 1 (0-2) | 0.07 (0.03-0.13) | 1 (1-2) | 0.04 (0.02-0.08) | -1.89 (-2.95--0.82) |
| Cook Islands | 0 (0-0) | 0.01 (0.01-0.02) | 0 (0-0) | 0.01 (0-0.02) | -0.19 (-0.58-0.2) |
| Costa Rica | 1 (1-2) | 0.08 (0.04-0.13) | 2 (1-3) | 0.03 (0.02-0.05) | -2.02 (-2.5--1.53) |
| Croatia | 12 (7-19) | 0.19 (0.1-0.3) | 23 (13-35) | 0.25 (0.14-0.38) | 2.83 (1.85-3.81) |
| Cuba | 18 (10-31) | 0.18 (0.1-0.3) | 44 (23-78) | 0.22 (0.12-0.4) | 1.9 (1.49-2.32) |
| Cyprus | 2 (1-3) | 0.27 (0.15-0.42) | 2 (1-3) | 0.07 (0.04-0.12) | -3.69 (-4.02--3.36) |
| Czechia | 18 (9-29) | 0.13 (0.07-0.21) | 13 (7-23) | 0.06 (0.03-0.11) | -1.38 (-2.03--0.73) |
| Democratic People's Republic of Korea | 7 (3-13) | 0.04 (0.02-0.07) | 14 (7-25) | 0.04 (0.02-0.07) | 0.75 (0.58-0.93) |
| Democratic Republic of the Congo | 8 (4-16) | 0.05 (0.02-0.09) | 16 (7-30) | 0.04 (0.02-0.08) | -0.73 (-1.87-0.42) |
| Denmark | 18 (10-25) | 0.22 (0.12-0.3) | 16 (10-23) | 0.13 (0.08-0.18) | -1.28 (-2.05--0.5) |
| Djibouti | 0 (0-0) | 0.05 (0.02-0.09) | 0 (0-1) | 0.05 (0.02-0.1) | 0.86 (-0.2-1.93) |
| Dominica | 0 (0-0) | 0.15 (0.09-0.23) | 0 (0-0) | 0.17 (0.1-0.27) | 1.15 (0.96-1.34) |
| Dominican Republic | 2 (1-4) | 0.06 (0.03-0.11) | 7 (3-13) | 0.07 (0.03-0.12) | 1.58 (1.04-2.12) |
| Ecuador | 2 (1-4) | 0.04 (0.02-0.07) | 5 (3-8) | 0.03 (0.02-0.05) | 0.23 (-0.38-0.84) |
| Egypt | 10 (4-18) | 0.03 (0.01-0.06) | 21 (9-39) | 0.03 (0.01-0.05) | 0.39 (-0.29-1.08) |

**Continued Supplementary Table 6.** Numbers, ASR, and Trends in Occupational Carcinogen-Attributable LC Deaths across countries and territories 1990–2021.

| Characteristics | 1990 | | 2021 | | 1990–2021 |
| --- | --- | --- | --- | --- | --- |
|  | Number of deaths  cases (95% UI) | The age-standardized deaths  rate/100000 (95% UI) | Number of deaths  cases (95% UI) | The age-standardized deaths  rate/100000 (95% UI) | Number of deaths  cases (95% UI) |
| El Salvador | 1 (0-2) | 0.03 (0.01-0.05) | 2 (1-3) | 0.03 (0.01-0.05) | 0.45 (-0.05-0.96) |
| Equatorial Guinea | 0 (0-0) | 0.05 (0.02-0.12) | 0 (0-0) | 0.03 (0.01-0.07) | -2.36 (-3.68--1.01) |
| Eritrea | 1 (0-2) | 0.08 (0.03-0.15) | 2 (1-4) | 0.07 (0.03-0.12) | -0.38 (-1.45-0.72) |
| Estonia | 2 (1-4) | 0.1 (0.04-0.18) | 1 (1-2) | 0.04 (0.02-0.07) | -2.43 (-3.13--1.73) |
| Eswatini | 1 (0-1) | 0.24 (0.12-0.41) | 1 (0-2) | 0.18 (0.08-0.34) | 0.01 (-1.42-1.46) |
| Ethiopia | 12 (5-23) | 0.05 (0.02-0.11) | 16 (7-31) | 0.03 (0.02-0.07) | -1.82 (-3.05--0.57) |
| Fiji | 0 (0-0) | 0.02 (0.01-0.04) | 0 (0-0) | 0.03 (0.01-0.05) | 2.08 (1.35-2.82) |
| Finland | 7 (4-10) | 0.09 (0.05-0.14) | 7 (4-10) | 0.05 (0.03-0.07) | -0.85 (-1.71-0.01) |
| France | 405 (244-574) | 0.49 (0.29-0.69) | 294 (170-424) | 0.2 (0.12-0.29) | -1.78 (-2.63--0.92) |
| Gabon | 0 (0-1) | 0.08 (0.03-0.15) | 1 (0-1) | 0.05 (0.02-0.1) | -1.65 (-2.4--0.88) |
| Gambia | 0 (0-0) | 0.02 (0.01-0.03) | 0 (0-0) | 0.01 (0.01-0.03) | -0.57 (-1.74-0.61) |
| Georgia | 10 (4-18) | 0.15 (0.07-0.27) | 11 (6-16) | 0.18 (0.1-0.28) | 1.76 (1.07-2.46) |
| Germany | 226 (126-330) | 0.18 (0.1-0.26) | 224 (129-332) | 0.11 (0.06-0.16) | -0.48 (-1.42-0.47) |
| Ghana | 2 (1-4) | 0.04 (0.02-0.06) | 4 (2-9) | 0.02 (0.01-0.05) | -1.12 (-2.04--0.19) |
| Greece | 26 (14-38) | 0.17 (0.09-0.25) | 22 (12-33) | 0.09 (0.05-0.13) | -1.37 (-2.23--0.5) |
| Greenland | 0 (0-0) | 0.08 (0.03-0.15) | 0 (0-0) | 0.14 (0.08-0.22) | 4.43 (3.65-5.22) |
| Grenada | 0 (0-0) | 0.1 (0.06-0.16) | 0 (0-0) | 0.07 (0.04-0.12) | 0.05 (-0.54-0.65) |
| Guam | 0 (0-0) | 0.02 (0.01-0.03) | 0 (0-0) | 0 (0-0.01) | -3.17 (-3.72--2.62) |
| Guatemala | 2 (1-4) | 0.06 (0.03-0.11) | 2 (1-4) | 0.02 (0.01-0.03) | -3.08 (-3.99--2.16) |
| Guinea | 1 (0-1) | 0.02 (0.01-0.04) | 2 (1-4) | 0.04 (0.02-0.07) | 1.58 (0.65-2.52) |

**Continued Supplementary Table 6.** Numbers, ASR, and Trends in Occupational Carcinogen-Attributable LC Deaths across countries and territories 1990–2021.

| Characteristics | 1990 | | 2021 | | 1990–2021 |
| --- | --- | --- | --- | --- | --- |
|  | Number of deaths  cases (95% UI) | The age-standardized deaths  rate/100000 (95% UI) | Number of deaths  cases (95% UI) | The age-standardized deaths  rate/100000 (95% UI) | Number of deaths  cases (95% UI) |
| Guinea-Bissau | 0 (0-0) | 0.06 (0.03-0.1) | 0 (0-1) | 0.05 (0.03-0.1) | 0.09 (-1.19-1.38) |
| Guyana | 0 (0-0) | 0.06 (0.04-0.1) | 0 (0-1) | 0.06 (0.03-0.1) | 1.47 (0.77-2.17) |
| Haiti | 5 (2-8) | 0.15 (0.08-0.25) | 11 (5-20) | 0.16 (0.08-0.29) | 0.66 (-0.32-1.64) |
| Honduras | 1 (1-2) | 0.05 (0.03-0.08) | 5 (3-9) | 0.09 (0.05-0.14) | 2.8 (1.83-3.78) |
| Hungary | 24 (11-42) | 0.17 (0.08-0.29) | 21 (11-39) | 0.12 (0.06-0.22) | -0.82 (-1.46--0.17) |
| Iceland | 0 (0-0) | 0.09 (0.05-0.12) | 0 (0-0) | 0.05 (0.03-0.07) | -1.25 (-1.74--0.75) |
| India | 629 (314-1072) | 0.12 (0.06-0.2) | 1262 (685-2065) | 0.1 (0.06-0.16) | -0.12 (-0.62-0.39) |
| Indonesia | 43 (19-77) | 0.04 (0.02-0.07) | 110 (50-203) | 0.04 (0.02-0.08) | 0.96 (0.46-1.47) |
| Iran (Islamic Republic of) | 19 (8-34) | 0.06 (0.03-0.11) | 33 (15-60) | 0.04 (0.02-0.07) | -0.27 (-0.85-0.32) |
| Iraq | 8 (5-14) | 0.11 (0.06-0.18) | 20 (10-34) | 0.09 (0.05-0.15) | -0.71 (-1.79-0.39) |
| Ireland | 9 (5-12) | 0.2 (0.12-0.29) | 5 (3-7) | 0.06 (0.03-0.09) | -3.18 (-3.6--2.76) |
| Israel | 4 (2-6) | 0.08 (0.04-0.12) | 6 (3-9) | 0.05 (0.03-0.07) | -1.68 (-1.91--1.44) |
| Italy | 421 (251-582) | 0.45 (0.27-0.63) | 274 (166-382) | 0.17 (0.1-0.23) | -2.34 (-3.35--1.31) |
| Jamaica | 1 (1-2) | 0.07 (0.04-0.11) | 2 (1-4) | 0.07 (0.03-0.12) | 0.13 (-0.33-0.59) |
| Japan | 73 (41-106) | 0.04 (0.03-0.06) | 134 (74-191) | 0.03 (0.02-0.04) | 0.46 (-0.73-1.65) |
| Jordan | 1 (1-2) | 0.09 (0.05-0.13) | 2 (1-4) | 0.03 (0.02-0.05) | -3.44 (-4.72--2.14) |
| Kazakhstan | 27 (15-42) | 0.19 (0.11-0.3) | 12 (7-20) | 0.06 (0.04-0.1) | -4.48 (-4.85--4.11) |
| Kenya | 3 (1-6) | 0.04 (0.01-0.07) | 15 (7-27) | 0.06 (0.03-0.11) | 2.21 (1-3.43) |
| Kiribati | 0 (0-0) | 0 (0-0) | 0 (0-0) | 0 (0-0.01) | 3.66 (2.91-4.43) |

**Continued Supplementary Table 6.** Numbers, ASR, and Trends in Occupational Carcinogen-Attributable LC Deaths across countries and territories 1990–2021.

| Characteristics | 1990 | | 2021 | | 1990–2021 |
| --- | --- | --- | --- | --- | --- |
|  | Number of deaths  cases (95% UI) | The age-standardized deaths  rate/100000 (95% UI) | Number of deaths  cases (95% UI) | The age-standardized deaths  rate/100000 (95% UI) | Number of deaths  cases (95% UI) |
| Kuwait | 0 (0-1) | 0.05 (0.03-0.08) | 1 (0-1) | 0.02 (0.01-0.03) | -0.76 (-1.9-0.39) |
| Kyrgyzstan | 3 (1-5) | 0.08 (0.04-0.15) | 2 (1-3) | 0.03 (0.02-0.06) | -2.93 (-3.44--2.42) |
| Lao People's Democratic Republic | 2 (1-3) | 0.07 (0.03-0.13) | 2 (1-4) | 0.05 (0.02-0.09) | -1.04 (-1.88--0.2) |
| Latvia | 4 (2-7) | 0.12 (0.06-0.2) | 2 (1-4) | 0.06 (0.03-0.1) | -1.74 (-2.42--1.05) |
| Lebanon | 5 (2-7) | 0.22 (0.12-0.34) | 8 (5-13) | 0.14 (0.07-0.21) | -0.43 (-0.78--0.08) |
| Lesotho | 1 (1-2) | 0.16 (0.09-0.26) | 4 (2-7) | 0.33 (0.15-0.59) | 2.92 (2.08-3.77) |
| Liberia | 0 (0-1) | 0.03 (0.01-0.06) | 1 (0-2) | 0.03 (0.01-0.06) | -0.31 (-1.43-0.82) |
| Libya | 2 (1-3) | 0.09 (0.04-0.16) | 4 (2-7) | 0.06 (0.03-0.12) | -0.23 (-1.03-0.57) |
| Lithuania | 6 (3-10) | 0.14 (0.07-0.23) | 4 (2-6) | 0.07 (0.04-0.12) | -1.95 (-2.58--1.31) |
| Luxembourg | 2 (1-2) | 0.31 (0.18-0.44) | 1 (1-2) | 0.12 (0.07-0.17) | -2.71 (-3.32--2.11) |
| Madagascar | 3 (1-6) | 0.06 (0.02-0.11) | 6 (3-12) | 0.05 (0.02-0.09) | -0.92 (-2.02-0.19) |
| Malawi | 1 (0-2) | 0.02 (0.01-0.04) | 2 (1-4) | 0.03 (0.01-0.05) | 0.41 (-0.77-1.61) |
| Malaysia | 6 (3-10) | 0.06 (0.03-0.11) | 15 (7-25) | 0.05 (0.03-0.09) | 0.06 (-0.59-0.71) |
| Maldives | 0 (0-0) | 0.06 (0.03-0.09) | 0 (0-0) | 0.02 (0.01-0.04) | -2.74 (-3.85--1.63) |
| Mali | 1 (1-3) | 0.03 (0.02-0.06) | 3 (1-5) | 0.03 (0.02-0.05) | -0.36 (-1.46-0.74) |
| Malta | 1 (1-2) | 0.24 (0.14-0.36) | 1 (1-1) | 0.1 (0.06-0.14) | -1.67 (-2.38--0.95) |
| Marshall Islands | 0 (0-0) | 0.02 (0.01-0.04) | 0 (0-0) | 0.02 (0.01-0.04) | 0.75 (-0.4-1.92) |
| Mauritania | 0 (0-0) | 0.03 (0.01-0.05) | 1 (0-1) | 0.03 (0.01-0.05) | -0.01 (-1-0.99) |
| Mauritius | 1 (0-1) | 0.08 (0.04-0.14) | 1 (0-2) | 0.05 (0.02-0.08) | 0.05 (-0.56-0.66) |

**Continued Supplementary Table 6.** Numbers, ASR, and Trends in Occupational Carcinogen-Attributable LC Deaths across countries and territories 1990–2021.

| Characteristics | 1990 | | 2021 | | 1990–2021 |
| --- | --- | --- | --- | --- | --- |
|  | Number of deaths  cases (95% UI) | The age-standardized deaths  rate/100000 (95% UI) | Number of deaths  cases (95% UI) | The age-standardized deaths  rate/100000 (95% UI) | Number of deaths  cases (95% UI) |
| Mexico | 47 (29-71) | 0.12 (0.07-0.17) | 63 (37-97) | 0.05 (0.03-0.08) | -1.69 (-2.32--1.06) |
| Micronesia (Federated States of) | 0 (0-0) | 0.03 (0.01-0.04) | 0 (0-0) | 0.02 (0.01-0.04) | 0.24 (-0.57-1.06) |
| Monaco | 1 (0-1) | 0.75 (0.39-1.29) | 0 (0-1) | 0.42 (0.24-0.69) | -1.69 (-2.93--0.43) |
| Mongolia | 1 (0-1) | 0.06 (0.03-0.1) | 1 (0-2) | 0.03 (0.02-0.06) | -1.53 (-2.28--0.77) |
| Montenegro | 1 (0-2) | 0.15 (0.06-0.25) | 2 (1-3) | 0.15 (0.07-0.27) | 0.74 (0.27-1.21) |
| Morocco | 9 (4-16) | 0.06 (0.03-0.11) | 18 (8-33) | 0.05 (0.02-0.09) | 0.33 (-0.17-0.84) |
| Mozambique | 4 (2-7) | 0.06 (0.02-0.11) | 10 (5-19) | 0.08 (0.04-0.15) | 1.18 (0.03-2.35) |
| Myanmar | 9 (4-17) | 0.04 (0.02-0.07) | 17 (9-32) | 0.03 (0.02-0.06) | 0.17 (-0.32-0.67) |
| Namibia | 1 (1-2) | 0.23 (0.13-0.38) | 3 (1-4) | 0.2 (0.1-0.34) | -0.95 (-2.05-0.17) |
| Nauru | 0 (0-0) | 0.03 (0.01-0.05) | 0 (0-0) | 0.02 (0.01-0.04) | -0.86 (-1.82-0.12) |
| Nepal | 12 (5-23) | 0.12 (0.05-0.21) | 19 (9-34) | 0.08 (0.04-0.14) | -0.53 (-1.19-0.14) |
| Netherlands | 52 (32-72) | 0.26 (0.16-0.35) | 44 (27-62) | 0.11 (0.07-0.16) | -2.22 (-2.92--1.51) |
| New Zealand | 6 (4-8) | 0.15 (0.09-0.21) | 6 (3-8) | 0.06 (0.04-0.09) | -2.22 (-2.77--1.66) |
| Nicaragua | 0 (0-1) | 0.03 (0.01-0.06) | 2 (1-3) | 0.03 (0.01-0.06) | 1.17 (0.27-2.08) |
| Niger | 1 (0-2) | 0.03 (0.02-0.06) | 3 (1-5) | 0.03 (0.01-0.06) | -0.09 (-1.44-1.29) |
| Nigeria | 22 (11-40) | 0.05 (0.02-0.09) | 30 (15-54) | 0.03 (0.02-0.05) | -1.8 (-2.88--0.7) |
| Niue | 0 (0-0) | 0.02 (0.01-0.03) | 0 (0-0) | 0.01 (0.01-0.02) | -0.5 (-0.86--0.14) |
| North Macedonia | 2 (1-4) | 0.09 (0.04-0.17) | 3 (1-6) | 0.08 (0.03-0.16) | 0.4 (0.01-0.79) |

**Continued Supplementary Table 6.** Numbers, ASR, and Trends in Occupational Carcinogen-Attributable LC Deaths across countries and territories 1990–2021.

| Characteristics | 1990 | | 2021 | | 1990–2021 |
| --- | --- | --- | --- | --- | --- |
|  | Number of deaths  cases (95% UI) | The age-standardized deaths  rate/100000 (95% UI) | Number of deaths  cases (95% UI) | The age-standardized deaths  rate/100000 (95% UI) | Number of deaths  cases (95% UI) |
| Northern Mariana Islands | 0 (0-0) | 0.02 (0.01-0.04) | 0 (0-0) | 0.01 (0.01-0.03) | -0.33 (-1.08-0.44) |
| Norway | 7 (4-10) | 0.1 (0.06-0.13) | 5 (3-8) | 0.05 (0.03-0.07) | -1.81 (-2.6--1.02) |
| Oman | 0 (0-0) | 0.02 (0.01-0.04) | 1 (0-1) | 0.03 (0.02-0.05) | 3.04 (1.53-4.56) |
| Pakistan | 93 (50-157) | 0.16 (0.09-0.27) | 254 (127-450) | 0.19 (0.1-0.33) | 0.32 (-0.58-1.23) |
| Palau | 0 (0-0) | 0.01 (0.01-0.02) | 0 (0-0) | 0.01 (0.01-0.02) | 0.44 (0.09-0.8) |
| Palestine | 0 (0-1) | 0.03 (0.02-0.06) | 1 (0-1) | 0.02 (0.01-0.04) | -1.63 (-2.79--0.45) |
| Panama | 1 (0-1) | 0.06 (0.03-0.09) | 1 (1-2) | 0.03 (0.01-0.05) | -1.49 (-1.92--1.05) |
| Papua New Guinea | 0 (0-0) | 0.01 (0-0.02) | 1 (0-1) | 0.01 (0-0.02) | -0.01 (-1.08-1.07) |
| Paraguay | 1 (1-2) | 0.06 (0.03-0.1) | 6 (3-12) | 0.11 (0.06-0.2) | 3.11 (2.5-3.73) |
| Peru | 9 (5-14) | 0.08 (0.04-0.12) | 10 (5-18) | 0.03 (0.02-0.06) | -2.75 (-3.38--2.12) |
| Philippines | 11 (5-19) | 0.03 (0.02-0.06) | 27 (13-48) | 0.03 (0.02-0.05) | 0.4 (-0.28-1.09) |
| Poland | 67 (32-116) | 0.15 (0.07-0.26) | 122 (69-183) | 0.17 (0.09-0.25) | 1.72 (1.12-2.32) |
| Portugal | 22 (11-38) | 0.16 (0.08-0.27) | 23 (12-35) | 0.09 (0.05-0.15) | -0.82 (-1.57--0.06) |
| Puerto Rico | 3 (2-5) | 0.1 (0.05-0.15) | 2 (1-4) | 0.03 (0.02-0.05) | -2.38 (-2.98--1.77) |
| Qatar | 0 (0-0) | 0.11 (0.05-0.18) | 1 (0-1) | 0.06 (0.03-0.12) | -1.6 (-3.29-0.13) |
| Republic of C么te d'Ivoire | 2 (1-4) | 0.05 (0.03-0.08) | 5 (2-10) | 0.04 (0.02-0.08) | -0.89 (-2.14-0.38) |
| Republic of Korea | 27 (12-52) | 0.09 (0.04-0.17) | 23 (11-39) | 0.02 (0.01-0.04) | -3.28 (-3.73--2.84) |
| Republic of Moldova | 4 (2-8) | 0.08 (0.04-0.16) | 3 (1-5) | 0.04 (0.02-0.08) | -1.88 (-2.34--1.42) |

**Continued Supplementary Table 6.** Numbers, ASR, and Trends in Occupational Carcinogen-Attributable LC Deaths across countries and territories 1990–2021.

| Characteristics | 1990 | | 2021 | | 1990–2021 |
| --- | --- | --- | --- | --- | --- |
|  | Number of deaths  cases (95% UI) | The age-standardized deaths  rate/100000 (95% UI) | Number of deaths  cases (95% UI) | The age-standardized deaths  rate/100000 (95% UI) | Number of deaths  cases (95% UI) |
| Romania | 40 (16-78) | 0.14 (0.06-0.27) | 41 (19-74) | 0.12 (0.06-0.22) | -0.2 (-0.77-0.38) |
| Russian Federation | 269 (131-469) | 0.14 (0.07-0.25) | 160 (87-250) | 0.07 (0.04-0.1) | -2.67 (-3.18--2.15) |
| Rwanda | 3 (1-5) | 0.1 (0.04-0.17) | 3 (1-6) | 0.05 (0.02-0.09) | -2.91 (-4.02--1.77) |
| Saint Kitts and Nevis | 0 (0-0) | 0.21 (0.12-0.31) | 0 (0-0) | 0.19 (0.11-0.28) | -0.1 (-0.41-0.21) |
| Saint Lucia | 0 (0-0) | 0.12 (0.07-0.18) | 0 (0-0) | 0.09 (0.04-0.14) | 0.05 (-0.41-0.52) |
| Saint Vincent and the Grenadines | 0 (0-0) | 0.12 (0.07-0.19) | 0 (0-0) | 0.14 (0.08-0.22) | 1.67 (1.28-2.07) |
| Samoa | 0 (0-0) | 0.01 (0-0.01) | 0 (0-0) | 0.01 (0-0.01) | -0.95 (-1.63--0.28) |
| San Marino | 0 (0-0) | 0.23 (0.11-0.39) | 0 (0-0) | 0.1 (0.04-0.19) | -0.74 (-1.74-0.26) |
| Sao Tome and Principe | 0 (0-0) | 0.02 (0.01-0.03) | 0 (0-0) | 0.02 (0.01-0.03) | -0.71 (-1.7-0.29) |
| Saudi Arabia | 1 (1-3) | 0.02 (0.01-0.04) | 3 (1-7) | 0.01 (0.01-0.03) | -1.02 (-1.9--0.13) |
| Senegal | 1 (1-2) | 0.04 (0.02-0.07) | 3 (1-5) | 0.04 (0.02-0.06) | -0.2 (-1.21-0.83) |
| Serbia | 17 (7-33) | 0.13 (0.06-0.26) | 18 (8-33) | 0.11 (0.05-0.21) | -0.44 (-1.08-0.2) |
| Seychelles | 0 (0-0) | 0.19 (0.1-0.31) | 0 (0-0) | 0.14 (0.07-0.24) | 0.54 (0.18-0.89) |
| Sierra Leone | 1 (0-1) | 0.03 (0.02-0.06) | 1 (1-2) | 0.03 (0.01-0.05) | -0.37 (-1.41-0.68) |
| Singapore | 3 (2-5) | 0.16 (0.09-0.24) | 3 (2-4) | 0.04 (0.02-0.05) | -4.42 (-4.94--3.9) |
| Slovakia | 9 (4-18) | 0.16 (0.07-0.31) | 8 (4-15) | 0.08 (0.04-0.16) | -1.32 (-1.78--0.86) |
| Slovenia | 4 (2-6) | 0.16 (0.09-0.26) | 6 (3-9) | 0.13 (0.07-0.21) | 0.54 (-0.24-1.32) |
| Solomon Islands | 0 (0-0) | 0.02 (0.01-0.03) | 0 (0-0) | 0.02 (0.01-0.04) | 2.3 (1.25-3.35) |

**Continued Supplementary Table 6.** Numbers, ASR, and Trends in Occupational Carcinogen-Attributable LC Deaths across countries and territories 1990–2021.

| Characteristics | 1990 | | 2021 | | 1990–2021 |
| --- | --- | --- | --- | --- | --- |
|  | Number of deaths  cases (95% UI) | The age-standardized deaths  rate/100000 (95% UI) | Number of deaths  cases (95% UI) | The age-standardized deaths  rate/100000 (95% UI) | Number of deaths  cases (95% UI) |
| Somalia | 2 (1-4) | 0.07 (0.03-0.14) | 5 (2-9) | 0.06 (0.03-0.13) | -0.69 (-1.96-0.6) |
| South Africa | 33 (19-53) | 0.16 (0.09-0.26) | 55 (32-83) | 0.12 (0.07-0.19) | -0.71 (-1.51-0.1) |
| South Sudan | 2 (1-3) | 0.06 (0.03-0.12) | 3 (1-5) | 0.06 (0.02-0.12) | -0.59 (-1.86-0.69) |
| Spain | 169 (96-251) | 0.31 (0.17-0.46) | 135 (75-203) | 0.13 (0.07-0.2) | -2.03 (-2.8--1.25) |
| Sri Lanka | 4 (2-6) | 0.04 (0.02-0.06) | 12 (5-23) | 0.04 (0.02-0.08) | 3 (2.51-3.5) |
| Sudan | 6 (2-13) | 0.06 (0.03-0.13) | 10 (4-19) | 0.05 (0.02-0.09) | -0.92 (-1.95-0.12) |
| Suriname | 0 (0-0) | 0.03 (0.02-0.05) | 0 (0-0) | 0.03 (0.02-0.06) | 1.55 (1.17-1.94) |
| Sweden | 11 (6-15) | 0.06 (0.04-0.09) | 9 (6-14) | 0.04 (0.02-0.05) | -1.1 (-2.1--0.08) |
| Switzerland | 19 (11-28) | 0.19 (0.11-0.27) | 16 (10-24) | 0.08 (0.05-0.12) | -1.5 (-2.3--0.7) |
| Syrian Arab Republic | 3 (1-5) | 0.05 (0.02-0.09) | 4 (2-8) | 0.03 (0.01-0.05) | -0.95 (-1.94-0.04) |
| Taiwan (Province of China) | 9 (5-16) | 0.05 (0.03-0.09) | 14 (8-22) | 0.03 (0.02-0.05) | -0.77 (-1.2--0.33) |
| Tajikistan | 2 (1-3) | 0.06 (0.03-0.1) | 2 (1-3) | 0.03 (0.02-0.06) | -1.9 (-2.81--0.98) |
| Thailand | 33 (17-55) | 0.09 (0.05-0.15) | 67 (31-120) | 0.06 (0.03-0.11) | -0.2 (-0.62-0.23) |
| Timor-Leste | 0 (0-0) | 0.03 (0.01-0.06) | 0 (0-1) | 0.03 (0.02-0.06) | 0.82 (-0.12-1.76) |
| Togo | 1 (0-1) | 0.04 (0.02-0.07) | 2 (1-4) | 0.05 (0.02-0.09) | 1.28 (0.08-2.49) |
| Tokelau | 0 (0-0) | 0.02 (0.01-0.03) | 0 (0-0) | 0.01 (0.01-0.02) | -1.04 (-1.21--0.86) |
| Tonga | 0 (0-0) | 0.01 (0.01-0.02) | 0 (0-0) | 0.01 (0.01-0.02) | -0.45 (-1.04-0.15) |
| Trinidad and Tobago | 1 (0-1) | 0.07 (0.04-0.12) | 1 (1-2) | 0.06 (0.03-0.1) | 0.42 (0.08-0.75) |

**Continued Supplementary Table 6.** Numbers, ASR, and Trends in Occupational Carcinogen-Attributable LC Deaths across countries and territories 1990–2021.

| Characteristics | 1990 | | 2021 | | 1990–2021 |
| --- | --- | --- | --- | --- | --- |
|  | Number of deaths  cases (95% UI) | The age-standardized deaths  rate/100000 (95% UI) | Number of deaths  cases (95% UI) | The age-standardized deaths  rate/100000 (95% UI) | Number of deaths  cases (95% UI) |
| Tunisia | 4 (2-7) | 0.07 (0.03-0.13) | 7 (3-13) | 0.05 (0.02-0.09) | -0.16 (-0.53-0.21) |
| Turkey | 142 (75-236) | 0.43 (0.23-0.71) | 153 (83-242) | 0.16 (0.09-0.26) | -2.37 (-2.85--1.89) |
| Turkmenistan | 2 (1-3) | 0.08 (0.04-0.14) | 2 (1-3) | 0.04 (0.02-0.07) | -1.85 (-2.45--1.25) |
| Tuvalu | 0 (0-0) | 0.02 (0.01-0.03) | 0 (0-0) | 0.02 (0.01-0.03) | -0.56 (-0.82--0.3) |
| Uganda | 5 (2-10) | 0.07 (0.03-0.13) | 11 (5-23) | 0.07 (0.03-0.13) | -1.25 (-2.62-0.14) |
| Ukraine | 99 (49-169) | 0.14 (0.07-0.23) | 50 (24-88) | 0.07 (0.03-0.12) | -2.92 (-3.53--2.3) |
| United Arab Emirates | 0 (0-1) | 0.05 (0.02-0.1) | 1 (1-3) | 0.02 (0.01-0.04) | -1.5 (-2.94--0.04) |
| United Kingdom | 210 (129-283) | 0.22 (0.14-0.3) | 189 (119-254) | 0.13 (0.08-0.18) | -1.46 (-2.25--0.67) |
| United Republic of Tanzania | 8 (3-16) | 0.07 (0.03-0.13) | 15 (6-30) | 0.05 (0.02-0.1) | -1.19 (-2.25--0.12) |
| United States of America | 447 (262-618) | 0.13 (0.08-0.19) | 471 (269-666) | 0.08 (0.04-0.11) | -1.77 (-2.32--1.22) |
| United States Virgin Islands | 0 (0-0) | 0.12 (0.07-0.2) | 0 (0-0) | 0.1 (0.05-0.17) | 1.36 (0.85-1.87) |
| Uruguay | 11 (7-17) | 0.28 (0.17-0.44) | 9 (5-14) | 0.17 (0.1-0.26) | -1.16 (-1.6--0.72) |
| Uzbekistan | 9 (4-16) | 0.07 (0.03-0.13) | 8 (4-15) | 0.03 (0.01-0.05) | -1.85 (-2.53--1.17) |
| Vanuatu | 0 (0-0) | 0.02 (0.01-0.03) | 0 (0-0) | 0.02 (0.01-0.03) | -0.07 (-1.05-0.91) |
| Venezuela (Bolivarian Republic of) | 11 (6-18) | 0.11 (0.06-0.19) | 26 (13-47) | 0.08 (0.04-0.15) | -0.41 (-0.99-0.18) |

**Continued Supplementary Table 6.** Numbers, ASR, and Trends in Occupational Carcinogen-Attributable LC Deaths across countries and territories 1990–2021.

| Characteristics | 1990 | | 2021 | | 1990–2021 |
| --- | --- | --- | --- | --- | --- |
|  | Number of deaths  cases (95% UI) | The age-standardized deaths  rate/100000 (95% UI) | Number of deaths  cases (95% UI) | The age-standardized deaths  rate/100000 (95% UI) | Number of deaths  cases (95% UI) |
| Viet Nam | 20 (10-37) | 0.05 (0.02-0.09) | 73 (34-137) | 0.07 (0.03-0.12) | 2.34 (1.85-2.84) |
| Yemen | 4 (1-7) | 0.06 (0.03-0.13) | 8 (3-16) | 0.05 (0.02-0.1) | -0.58 (-1.78-0.63) |
| Zambia | 3 (1-5) | 0.08 (0.03-0.15) | 7 (2-21) | 0.09 (0.03-0.25) | 0.09 (-1.22-1.43) |
| Zimbabwe | 4 (2-6) | 0.09 (0.04-0.15) | 5 (3-9) | 0.07 (0.04-0.12) | -0.27 (-1.41-0.87) |

**Supplementary Table 7.** Global Numbers, ASR, and Trends in Occupational Carcinogen-Attributable LC DALYs: 1990–2021.

| Characteristics | 1990 | | 2021 | | 1990–2021 |
| --- | --- | --- | --- | --- | --- |
|  | Number of DALYs cases (95% UI) | The age-standardized DALYs rate/100000 (95% UI) | Number of DALYs cases (95% UI) | The age-standardized DALYs rate/100000 (95% UI) | EAPC (95% CI) |
| Global | 147464 (88716-223102) | 3.58 (2.18-5.35) | 179887 (106890-278828) | 2.05 (1.23-3.17) | -1.33 (-1.54--1.12) |
| **Sex** | | | | | |
| Female | 10372 (5356-17127) | 0.48 (0.25-0.79) | 16423 (8237-27539) | 0.36 (0.18-0.61) | -1.08 (-1.13--1.03) |
| Male | 137092 (82390-206564) | 7.14 (4.33-10.52) | 163463 (97998-251462) | 3.94 (2.39-5.98) | -2.04 (-2.1--1.98) |
| **Age** | | | | | |
| 20-24 years | 0 (0-0) | 0 (0-0) | 0 (0-0) | 0 (0-0) | -4.11 (-5.1--3.1) |
| 25-29 years | 398 (161-749) | 0.09 (0.04-0.17) | 375 (152-702) | 0.06 (0.03-0.12) | -1.29 (-1.36--1.22) |
| 30-34 years | 726 (294-1330) | 0.19 (0.08-0.35) | 769 (308-1436) | 0.13 (0.05-0.24) | -1.44 (-1.52--1.37) |
| 35-39 years | 2133 (880-3812) | 0.61 (0.25-1.08) | 2034 (822-3725) | 0.36 (0.15-0.66) | -1.85 (-1.91--1.78) |
| 40-44 years | 6641 (2841-11703) | 2.32 (0.99-4.09) | 6469 (2783-11636) | 1.29 (0.56-2.33) | -2.39 (-2.53--2.25) |
| 45-49 years | 10742 (4877-18721) | 4.63 (2.1-8.06) | 12314 (5823-21372) | 2.6 (1.23-4.51) | -2.07 (-2.17--1.96) |
| 50-54 years | 21314 (10515-36023) | 10.03 (4.95-16.95) | 23294 (11483-39562) | 5.24 (2.58-8.89) | -2.26 (-2.33--2.19) |
| 55-59 years | 25375 (14177-40288) | 13.7 (7.66-21.75) | 28799 (15574-47626) | 7.28 (3.94-12.04) | -2.22 (-2.38--2.06) |
| 60-64 years | 28478 (16533-44189) | 17.73 (10.29-27.51) | 30868 (17198-49773) | 9.64 (5.37-15.55) | -2.03 (-2.13--1.94) |
| 65-69 years | 20833 (12702-29764) | 16.85 (10.28-24.08) | 27003 (16519-41348) | 9.79 (5.99-14.99) | -1.98 (-2.07--1.89) |
| 70-74 years | 13251 (8129-18369) | 15.65 (9.6-21.7) | 21009 (13249-29670) | 10.21 (6.44-14.41) | -1.68 (-1.81--1.55) |

**Continued Supplementary Table 7.** Global Numbers, ASR, and Trends in Occupational Carcinogen-Attributable LC DALYs: 1990–2021.

| Characteristics | 1990 | | 2021 | | 1990–2021 |
| --- | --- | --- | --- | --- | --- |
|  | Number of DALYs cases (95% UI) | The age-standardized DALYs rate/100000 (95% UI) | Number of DALYs cases (95% UI) | The age-standardized DALYs rate/100000 (95% UI) | Number of DALYs cases (95% UI) |
| 75-79 years | 9966 (6192-14170) | 16.19 (10.06-23.02) | 12690 (7758-17962) | 9.62 (5.88-13.62) | -1.53 (-1.59--1.48) |
| 80-84 years | 5262 (3156-7579) | 14.87 (8.92-21.43) | 8130 (4740-11894) | 9.28 (5.41-13.58) | -1.31 (-1.45--1.17) |
| 85-89 years | 1855 (1048-2677) | 12.28 (6.93-17.72) | 4192 (2375-6138) | 9.17 (5.19-13.43) | -0.67 (-0.8--0.53) |
| 90-94 years | 416 (239-609) | 9.72 (5.57-14.2) | 1569 (859-2282) | 8.77 (4.8-12.76) | -0.09 (-0.25-0.07) |
| 95+ years | 73 (39-108) | 7.18 (3.83-10.64) | 370 (192-555) | 6.79 (3.53-10.19) | -0.17 (-0.27--0.06) |
| **SDI regions** | | | | | |
| High-middle SDI | 47106 (27213-71461) | 4.54 (2.64-6.85) | 38743 (23013-59496) | 1.94 (1.15-2.98) | -3 (-3.09--2.91) |
| High SDI | 43142 (25504-61957) | 3.93 (2.31-5.64) | 36925 (21830-51467) | 1.77 (1.04-2.48) | -2.58 (-2.68--2.48) |
| Low-middle SDI | 20671 (10141-36144) | 2.95 (1.49-5.1) | 41706 (21742-70944) | 2.65 (1.41-4.48) | -0.37 (-0.41--0.32) |
| Low SDI | 6304 (2901-11444) | 2.39 (1.12-4.28) | 11189 (5571-19969) | 1.93 (1-3.39) | -0.92 (-1--0.83) |
| Middle SDI | 30061 (15291-51885) | 2.6 (1.36-4.42) | 51101 (26754-84082) | 1.78 (0.95-2.9) | -1.39 (-1.45--1.32) |
| **GBD regions** | | | | | |
| Advanced Health System | 77725 (43999-112941) | 4.78 (2.68-7.02) | 58834 (34943-83541) | 2.12 (1.24-3.08) | -2.15 (-2.69--1.61) |
| Africa | 5530 (2868-9472) | 1.73 (0.93-2.93) | 10293 (5376-17745) | 1.37 (0.74-2.33) | -0.86 (-1.78-0.08) |
| African Region | 4473 (2362-7722) | 1.79 (0.94-3.05) | 8226 (4360-13977) | 1.41 (0.78-2.36) | -1 (-2.01-0.02) |
| America | 22341 (13873-32077) | 3.7 (2.29-5.31) | 28139 (17561-40809) | 2.11 (1.32-3.07) | -1.3 (-1.49--1.11) |

**Continued Supplementary Table 7.** Global Numbers, ASR, and Trends in Occupational Carcinogen-Attributable LC DALYs: 1990–2021.

| Characteristics | 1990 | | 2021 | | 1990–2021 |
| --- | --- | --- | --- | --- | --- |
|  | Number of DALYs cases (95% UI) | The age-standardized DALYs rate/100000 (95% UI) | Number of DALYs cases (95% UI) | The age-standardized DALYs rate/100000 (95% UI) | Number of DALYs cases (95% UI) |
| Andean Latin America | 341 (190-570) | 1.64 (0.91-2.7) | 543 (281-936) | 0.91 (0.48-1.55) | -1.39 (-1.93--0.85) |
| Asia | 56464 (29233-99420) | 2.49 (1.32-4.29) | 97039 (49487-163758) | 1.82 (0.94-3.06) | -0.35 (-0.61--0.09) |
| Australasia | 1282 (757-1769) | 5.36 (3.16-7.39) | 953 (565-1352) | 1.71 (1-2.44) | -3.19 (-3.69--2.7) |
| Basic Health System | 38265 (19599-66833) | 2.35 (1.24-4.04) | 60995 (31836-101229) | 1.53 (0.81-2.52) | -0.49 (-0.76--0.22) |
| Caribbean | 906 (495-1489) | 3.45 (1.89-5.65) | 2008 (1035-3493) | 3.69 (1.9-6.41) | 1.15 (0.91-1.38) |
| Central Africa | 476 (213-881) | 1.45 (0.66-2.65) | 1004 (461-1821) | 1.25 (0.58-2.22) | -0.55 (-1.58-0.5) |
| Central Asia | 2237 (1167-3794) | 4.28 (2.27-7.19) | 1507 (795-2450) | 1.62 (0.89-2.57) | -2.96 (-3.28--2.63) |
| Central Europe | 6578 (3010-11953) | 4.31 (1.94-7.87) | 7267 (3954-11780) | 3.52 (1.86-5.84) | 0.06 (-0.49-0.61) |
| Central Latin America | 2277 (1253-3755) | 2.61 (1.47-4.25) | 3321 (1854-5386) | 1.29 (0.73-2.09) | -1.6 (-2.13--1.07) |
| Central Sub-Saharan Africa | 428 (178-817) | 1.6 (0.67-3) | 794 (370-1461) | 1.18 (0.56-2.17) | -1.04 (-2.07-0) |
| Commonwealth High Income | 7570 (4590-10345) | 4.98 (3.01-6.83) | 5970 (3720-8143) | 2.13 (1.33-2.91) | -2.33 (-2.93--1.73) |
| Commonwealth Low Income | 3156 (1509-5422) | 3.23 (1.59-5.49) | 6081 (2832-10950) | 2.37 (1.14-4.23) | -0.42 (-1.31-0.48) |
| Commonwealth Middle Income | 25872 (12914-44953) | 3.53 (1.81-6.05) | 49608 (26311-84383) | 2.89 (1.56-4.84) | -0.41 (-0.92-0.11) |
| East Asia | 19337 (9032-34980) | 1.99 (0.95-3.55) | 29193 (13242-52261) | 1.27 (0.58-2.27) | -0.46 (-0.77--0.15) |

**Continued Supplementary Table 7.** Global Numbers, ASR, and Trends in Occupational Carcinogen-Attributable LC DALYs: 1990–2021.

| Characteristics | 1990 | | 2021 | | 1990–2021 |
| --- | --- | --- | --- | --- | --- |
|  | Number of DALYs cases (95% UI) | The age-standardized DALYs rate/100000 (95% UI) | Number of DALYs cases (95% UI) | The age-standardized DALYs rate/100000 (95% UI) | Number of DALYs cases (95% UI) |
| East Asia & Pacific - WB | 27088 (13995-46734) | 1.86 (0.99-3.16) | 42701 (21492-72328) | 1.26 (0.64-2.12) | -0.35 (-0.61--0.09) |
| Eastern Africa | 1536 (655-2923) | 1.88 (0.8-3.57) | 2887 (1304-5345) | 1.5 (0.69-2.79) | -0.94 (-2-0.13) |
| Eastern Europe | 12854 (5981-23248) | 4.44 (2.06-8.07) | 6669 (3547-11080) | 1.97 (1.02-3.33) | -2.99 (-3.5--2.48) |
| Eastern Mediterranean Region | 5430 (2707-9665) | 2.67 (1.35-4.7) | 12966 (6372-22778) | 2.41 (1.21-4.16) | -0.1 (-0.82-0.63) |
| Eastern Sub-Saharan Africa | 1630 (713-2982) | 1.85 (0.8-3.4) | 3291 (1432-6088) | 1.6 (0.72-2.99) | -0.66 (-1.75-0.43) |
| Europe | 62862 (35354-91967) | 6.1 (3.46-8.99) | 44136 (26338-62931) | 2.87 (1.69-4.2) | -1.96 (-2.52--1.4) |
| Europe & Central Asia - WB | 64129 (36430-93666) | 6.05 (3.47-8.87) | 44850 (26784-63717) | 2.82 (1.67-4.11) | -2.03 (-2.53--1.52) |
| European Region | 64292 (36526-93930) | 6.02 (3.45-8.84) | 45077 (26921-64059) | 2.8 (1.66-4.09) | -2.03 (-2.54--1.53) |
| High-income Asia Pacific | 2426 (1276-4052) | 1.19 (0.63-1.96) | 2746 (1526-4060) | 0.55 (0.31-0.83) | -1.13 (-1.98--0.28) |
| High-income North America | 11386 (6530-16133) | 3.24 (1.83-4.64) | 10738 (6182-15390) | 1.59 (0.9-2.3) | -2.15 (-2.66--1.64) |
| Latin America & Caribbean - WB | 11037 (6405-17993) | 3.92 (2.29-6.31) | 17455 (10366-27716) | 2.42 (1.45-3.83) | -0.66 (-1.01--0.3) |
| Limited Health System | 30205 (14715-53099) | 3.26 (1.64-5.65) | 57693 (30020-99239) | 2.64 (1.4-4.47) | -0.42 (-1.02-0.19) |
| Middle East & North Africa - WB | 2339 (1173-4011) | 1.68 (0.87-2.84) | 4531 (2252-7751) | 1.16 (0.61-1.95) | -0.5 (-1.13-0.14) |

**Continued Supplementary Table 7.** Global Numbers, ASR, and Trends in Occupational Carcinogen-Attributable LC DALYs: 1990–2021.

| Characteristics | 1990 | | 2021 | | 1990–2021 |
| --- | --- | --- | --- | --- | --- |
|  | Number of DALYs cases (95% UI) | The age-standardized DALYs rate/100000 (95% UI) | Number of DALYs cases (95% UI) | The age-standardized DALYs rate/100000 (95% UI) | Number of DALYs cases (95% UI) |
| Minimal Health System | 1090 (489-1961) | 1.58 (0.72-2.82) | 2141 (1017-3943) | 1.4 (0.66-2.56) | -0.57 (-1.61-0.49) |
| North Africa and Middle East | 6358 (3432-10424) | 3.51 (1.92-5.76) | 8680 (4940-14134) | 1.78 (1.04-2.89) | -1.81 (-2.48--1.14) |
| North America | 11390 (6533-16137) | 3.24 (1.83-4.64) | 10739 (6183-15393) | 1.59 (0.9-2.3) | -2.15 (-2.66--1.64) |
| Northern Africa | 939 (398-1701) | 1.32 (0.57-2.37) | 1869 (812-3298) | 1.05 (0.47-1.83) | 0.01 (-0.52-0.53) |
| Oceania | 13 (6-25) | 0.38 (0.18-0.69) | 30 (15-53) | 0.35 (0.18-0.6) | 0.14 (-0.69-0.96) |
| Region of the Americas | 22341 (13873-32077) | 3.7 (2.29-5.31) | 28139 (17561-40809) | 2.11 (1.32-3.07) | -1.3 (-1.49--1.11) |
| South-East Asia Region | 26515 (12998-46864) | 3.17 (1.6-5.53) | 48780 (25686-82921) | 2.47 (1.32-4.13) | -0.27 (-0.69-0.14) |
| South Asia | 26338 (12914-46091) | 3.88 (1.96-6.7) | 50285 (26069-86565) | 3.12 (1.64-5.3) | -0.31 (-0.79-0.18) |
| South Asia - WB | 26650 (13062-46671) | 3.81 (1.92-6.58) | 50878 (26309-87511) | 3.08 (1.62-5.23) | -0.3 (-0.78-0.19) |
| Southeast Asia | 4142 (2012-7075) | 1.43 (0.71-2.39) | 10126 (4976-17738) | 1.37 (0.69-2.39) | 0.76 (0.35-1.16) |
| Southern Africa | 1487 (850-2330) | 3.15 (1.81-4.91) | 2636 (1568-4170) | 2.58 (1.54-3.94) | -0.84 (-1.81-0.13) |
| Southern Latin America | 2662 (1508-4301) | 5.67 (3.22-9.16) | 2168 (1333-3188) | 2.52 (1.55-3.73) | -1.82 (-2.01--1.63) |
| Southern Sub-Saharan Africa | 1151 (673-1813) | 4.04 (2.39-6.3) | 1805 (1085-2661) | 3.02 (1.8-4.49) | -0.84 (-1.65--0.03) |
| Sub-Saharan Africa - WB | 4596 (2420-7969) | 1.84 (0.97-3.15) | 8428 (4463-14472) | 1.47 (0.8-2.48) | -1.01 (-2.05-0.03) |

**Continued Supplementary Table 7.** Global Numbers, ASR, and Trends in Occupational Carcinogen-Attributable LC DALYs: 1990–2021.

| Characteristics | 1990 | | 2021 | | 1990–2021 |
| --- | --- | --- | --- | --- | --- |
|  | Number of DALYs cases (95% UI) | The age-standardized DALYs rate/100000 (95% UI) | Number of DALYs cases (95% UI) | The age-standardized DALYs rate/100000 (95% UI) | Number of DALYs cases (95% UI) |
| Tropical Latin America | 4887 (2837-7737) | 4.9 (2.94-7.68) | 9487 (5564-15010) | 3.58 (2.11-5.63) | -0.01 (-0.34-0.32) |
| Western Africa | 1091 (568-1964) | 1.23 (0.65-2.21) | 1897 (932-3307) | 0.92 (0.47-1.58) | -1.16 (-2.17--0.15) |
| Western Europe | 39058 (23053-54450) | 6.93 (4.1-9.78) | 26087 (15144-36624) | 2.85 (1.65-4.02) | -2.16 (-2.9--1.42) |
| Western Pacific Region | 23861 (12316-40784) | 1.92 (1.03-3.24) | 36021 (17904-61266) | 1.25 (0.62-2.11) | -0.45 (-0.73--0.16) |
| Western Sub-Saharan Africa | 1172 (609-2094) | 1.2 (0.63-2.13) | 2188 (1065-3808) | 0.95 (0.48-1.64) | -0.94 (-1.95-0.09) |

**Supplementary Table 8.** Numbers, ASR, and Trends in Occupational Carcinogen-Attributable LC DALYs across countries and territories 1990–2021.

| Characteristics | 1990 | | 2021 | | 1990–2021 |
| --- | --- | --- | --- | --- | --- |
|  | Number of DALYs cases (95% UI) | The age-standardized DALYs rate/100000 (95% UI) | Number of DALYs cases (95% UI) | The age-standardized DALYs rate/100000 (95% UI) | EAPC (95% CI) |
| Afghanistan | 209 (79-457) | 2.66 (1.03-5.78) | 266 (96-543) | 1.93 (0.76-3.96) | -2.34 (-3.37--1.3) |
| Albania | 56 (25-99) | 2.53 (1.19-4.39) | 82 (34-157) | 1.9 (0.77-3.64) | 0.99 (0.57-1.41) |
| Algeria | 147 (61-274) | 1.11 (0.48-2.03) | 292 (128-544) | 0.75 (0.34-1.36) | 0 (-0.63-0.65) |
| American Samoa | 0 (0-1) | 1.33 (0.73-2.19) | 0 (0-0) | 0.35 (0.17-0.63) | -3.9 (-4.57--3.23) |
| Andorra | 3 (2-7) | 5.92 (2.87-11.09) | 4 (2-7) | 2.63 (1.14-4.67) | -1.87 (-2.34--1.39) |
| Angola | 91 (36-184) | 1.89 (0.77-3.85) | 171 (80-331) | 1.19 (0.55-2.37) | -1.61 (-2.72--0.49) |
| Antigua and Barbuda | 1 (1-2) | 2.21 (1.12-3.8) | 2 (1-3) | 1.67 (0.81-2.89) | -0.02 (-0.38-0.35) |
| Argentina | 2082 (1150-3378) | 6.37 (3.52-10.33) | 1702 (1030-2479) | 3.09 (1.87-4.56) | -1.66 (-1.85--1.47) |
| Armenia | 232 (140-354) | 7.5 (4.63-11.46) | 149 (88-223) | 3.43 (2.02-5.11) | -1.86 (-2.19--1.52) |
| Australia | 1148 (682-1596) | 5.77 (3.42-7.99) | 843 (501-1196) | 1.8 (1.05-2.56) | -3.27 (-3.77--2.76) |
| Austria | 425 (234-663) | 3.82 (2.07-6.15) | 310 (173-452) | 1.77 (0.98-2.63) | -1.55 (-2.23--0.87) |
| Azerbaijan | 303 (140-554) | 5.15 (2.39-9.37) | 229 (102-438) | 1.8 (0.83-3.39) | -3.07 (-3.37--2.77) |
| Bahamas | 6 (3-9) | 3.52 (2.05-5.41) | 14 (7-24) | 3.06 (1.57-5.29) | 0.52 (0.15-0.89) |
| Bahrain | 7 (4-12) | 3.61 (1.97-5.92) | 20 (11-34) | 2.36 (1.24-4) | -0.99 (-2.27-0.3) |
| Bangladesh | 2336 (1102-4041) | 4.53 (2.19-7.73) | 4178 (1925-7508) | 2.83 (1.33-5.06) | -0.36 (-1.09-0.39) |
| Barbados | 5 (3-8) | 1.9 (1.06-3.11) | 10 (5-17) | 1.99 (1.04-3.36) | 1.02 (0.59-1.45) |
| Belarus | 591 (274-1112) | 4.45 (2.03-8.41) | 450 (211-840) | 2.89 (1.35-5.46) | -2.05 (-2.59--1.51) |
| Belgium | 1182 (694-1711) | 7.76 (4.57-11.21) | 720 (428-1024) | 3.13 (1.85-4.45) | -2.43 (-3.15--1.71) |
| Belize | 2 (1-3) | 2.29 (1.39-3.48) | 9 (5-14) | 2.81 (1.69-4.3) | 1.06 (0.14-1.98) |
| Benin | 23 (12-40) | 1.09 (0.56-1.87) | 64 (31-123) | 1.1 (0.53-2.1) | 0.24 (-0.95-1.43) |

**Continued Supplementary Table 8.** Numbers, ASR, and Trends in Occupational Carcinogen-Attributable LC DALYs across countries and territories 1990–2021.

| Characteristics | 1990 | | 2021 | | 1990–2021 |
| --- | --- | --- | --- | --- | --- |
|  | Number of DALYs cases (95% UI) | The age-standardized DALYs rate/100000 (95% UI) | Number of DALYs cases (95% UI) | The age-standardized DALYs rate/100000 (95% UI) | Number of DALYs cases (95% UI) |
| Bermuda | 5 (3-7) | 8.15 (5.12-11.6) | 4 (2-6) | 2.73 (1.54-4.25) | -2.09 (-2.7--1.47) |
| Bhutan | 8 (3-15) | 2.67 (1.09-5.15) | 14 (7-27) | 2.26 (1.03-4.16) | 0.32 (-0.33-0.98) |
| Bolivia (Plurinational State of) | 66 (34-117) | 2.02 (1.06-3.5) | 166 (82-294) | 1.8 (0.91-3.13) | 0.16 (-0.49-0.82) |
| Bosnia and Herzegovina | 160 (74-285) | 3.46 (1.7-6.12) | 164 (73-304) | 2.69 (1.16-5.05) | -0.31 (-0.87-0.24) |
| Botswana | 25 (13-43) | 4.25 (2.18-7.22) | 35 (17-65) | 2.34 (1.14-4.17) | -2.62 (-3.69--1.53) |
| Brazil | 4847 (2815-7680) | 4.98 (3-7.8) | 9307 (5469-14696) | 3.59 (2.13-5.64) | -0.04 (-0.37-0.29) |
| Brunei Darussalam | 2 (1-4) | 2.37 (1.25-3.8) | 3 (1-4) | 0.85 (0.46-1.34) | -2.1 (-3.23--0.95) |
| Bulgaria | 303 (97-644) | 2.45 (0.76-5.23) | 333 (123-703) | 2.77 (0.99-5.92) | 0.95 (0.29-1.61) |
| Burkina Faso | 53 (27-96) | 1.1 (0.57-1.96) | 113 (52-204) | 1.09 (0.51-1.94) | -0.04 (-1.08-1.01) |
| Burundi | 67 (27-131) | 2.58 (1.03-5.04) | 106 (46-211) | 1.75 (0.76-3.43) | -1.73 (-2.79--0.65) |
| Cabo Verde | 2 (1-3) | 0.85 (0.41-1.57) | 4 (2-8) | 0.78 (0.37-1.52) | 0.01 (-0.75-0.78) |
| Cambodia | 102 (44-182) | 1.98 (0.88-3.48) | 293 (119-560) | 2.09 (0.86-3.95) | 1.1 (0.36-1.83) |
| Cameroon | 52 (26-92) | 1.04 (0.5-1.81) | 197 (81-384) | 1.34 (0.57-2.6) | 1.05 (-0.03-2.14) |
| Canada | 1469 (869-2108) | 4.47 (2.63-6.43) | 1178 (740-1644) | 1.55 (0.96-2.19) | -2.47 (-3.07--1.87) |
| Central African Republic | 31 (13-63) | 2.24 (0.92-4.38) | 47 (18-95) | 1.61 (0.67-3.39) | -1.12 (-2.11--0.12) |
| Chad | 20 (10-36) | 0.68 (0.34-1.24) | 77 (33-145) | 1.18 (0.5-2.21) | 1.48 (0.27-2.71) |
| Chile | 282 (170-436) | 2.73 (1.66-4.19) | 252 (152-384) | 0.98 (0.59-1.51) | -2.15 (-2.41--1.89) |

**Continued Supplementary Table 8.** Numbers, ASR, and Trends in Occupational Carcinogen-Attributable LC DALYs across countries and territories 1990–2021.

| Characteristics | 1990 | | 2021 | | 1990–2021 |
| --- | --- | --- | --- | --- | --- |
|  | Number of DALYs cases (95% UI) | The age-standardized DALYs rate/100000 (95% UI) | Number of DALYs cases (95% UI) | The age-standardized DALYs rate/100000 (95% UI) | Number of DALYs cases (95% UI) |
| China | 18817 (8790-34104) | 2.02 (0.96-3.59) | 28347 (12838-50814) | 1.28 (0.59-2.28) | -0.47 (-0.78--0.16) |
| Colombia | 530 (282-896) | 2.8 (1.52-4.66) | 627 (342-1064) | 1.13 (0.62-1.92) | -2.26 (-2.68--1.83) |
| Comoros | 3 (1-7) | 1.43 (0.57-2.8) | 7 (3-13) | 1.16 (0.51-2.31) | -0.4 (-1.16-0.36) |
| Congo | 24 (11-48) | 2.02 (0.92-3.96) | 37 (16-72) | 1.15 (0.49-2.23) | -2.02 (-2.95--1.08) |
| Cook Islands | 0 (0-0) | 0.37 (0.18-0.64) | 0 (0-0) | 0.25 (0.12-0.47) | -0.14 (-0.5-0.22) |
| Costa Rica | 37 (19-64) | 2.09 (1.05-3.56) | 46 (24-80) | 0.83 (0.43-1.42) | -2.27 (-2.71--1.83) |
| Croatia | 351 (184-564) | 5.33 (2.79-8.56) | 517 (278-793) | 5.87 (3.17-9.17) | 1.96 (1.05-2.88) |
| Cuba | 502 (256-861) | 4.95 (2.52-8.51) | 1212 (605-2190) | 6.26 (3.1-11.35) | 2.03 (1.63-2.44) |
| Cyprus | 37 (21-57) | 4.86 (2.71-7.53) | 31 (16-50) | 1.45 (0.73-2.37) | -3.37 (-3.72--3.03) |
| Czechia | 510 (239-885) | 3.86 (1.77-6.75) | 333 (159-593) | 1.74 (0.79-3.18) | -1.82 (-2.42--1.22) |
| Democratic People's Republic of Korea | 235 (98-445) | 1.21 (0.52-2.28) | 449 (196-790) | 1.26 (0.56-2.22) | 0.61 (0.43-0.8) |
| Democratic Republic of the Congo | 263 (112-500) | 1.42 (0.62-2.65) | 517 (224-942) | 1.15 (0.5-2.16) | -0.69 (-1.71-0.34) |
| Denmark | 407 (231-574) | 5.3 (3-7.63) | 328 (197-455) | 2.71 (1.62-3.8) | -1.76 (-2.46--1.05) |
| Djibouti | 3 (1-5) | 1.45 (0.58-2.84) | 13 (5-25) | 1.54 (0.64-3.07) | 0.68 (-0.21-1.58) |
| Dominica | 2 (1-3) | 3.53 (2.02-5.5) | 4 (2-6) | 4.12 (2.22-6.65) | 1.42 (1.2-1.64) |
| Dominican Republic | 72 (32-127) | 1.79 (0.82-3.17) | 206 (93-377) | 1.99 (0.91-3.64) | 1.52 (1.03-2.01) |
| Ecuador | 58 (29-101) | 1.05 (0.53-1.81) | 122 (61-214) | 0.74 (0.37-1.29) | -0.25 (-0.78-0.27) |
| Egypt | 333 (141-608) | 0.98 (0.42-1.8) | 680 (279-1285) | 0.87 (0.37-1.62) | 0.15 (-0.41-0.72) |

**Continued Supplementary Table 8.** Numbers, ASR, and Trends in Occupational Carcinogen-Attributable LC DALYs across countries and territories 1990–2021.

| Characteristics | 1990 | | 2021 | | 1990–2021 |
| --- | --- | --- | --- | --- | --- |
|  | Number of DALYs cases (95% UI) | The age-standardized DALYs rate/100000 (95% UI) | Number of DALYs cases (95% UI) | The age-standardized DALYs rate/100000 (95% UI) | Number of DALYs cases (95% UI) |
| El Salvador | 26 (10-48) | 0.82 (0.32-1.55) | 45 (18-80) | 0.75 (0.31-1.35) | 0.37 (-0.12-0.86) |
| Equatorial Guinea | 4 (1-8) | 1.59 (0.66-3.43) | 6 (2-12) | 0.92 (0.34-1.92) | -2.59 (-3.79--1.37) |
| Eritrea | 41 (18-80) | 2.47 (1.06-4.85) | 78 (31-147) | 2.08 (0.85-3.9) | -0.51 (-1.46-0.46) |
| Estonia | 63 (25-121) | 3.1 (1.21-5.99) | 27 (13-47) | 1.18 (0.57-2.1) | -3.17 (-3.79--2.55) |
| Eswatini | 18 (9-33) | 6.01 (3.09-10.7) | 27 (12-50) | 4.54 (2.1-8.39) | -0.11 (-1.42-1.22) |
| Ethiopia | 389 (160-771) | 1.64 (0.68-3.2) | 501 (220-973) | 1 (0.45-1.97) | -2.03 (-3.16--0.89) |
| Fiji | 2 (1-4) | 0.61 (0.32-1.05) | 6 (3-10) | 0.67 (0.34-1.2) | 1.75 (1.16-2.34) |
| Finland | 160 (92-232) | 2.27 (1.3-3.31) | 144 (83-210) | 1.15 (0.65-1.67) | -1.2 (-1.98--0.42) |
| France | 9461 (5495-13842) | 12.08 (6.89-17.78) | 6230 (3587-9036) | 4.77 (2.8-6.92) | -1.95 (-2.73--1.17) |
| Gabon | 14 (6-26) | 2.28 (0.96-4.31) | 17 (7-33) | 1.41 (0.58-2.67) | -1.73 (-2.41--1.05) |
| Gambia | 2 (1-4) | 0.5 (0.25-0.92) | 5 (2-9) | 0.43 (0.21-0.79) | -0.58 (-1.69-0.54) |
| Georgia | 337 (142-613) | 5.06 (2.13-9.17) | 284 (163-442) | 4.97 (2.86-7.76) | 1.05 (0.44-1.68) |
| Germany | 5376 (2958-7977) | 4.42 (2.45-6.66) | 4360 (2518-6310) | 2.29 (1.29-3.3) | -1.25 (-2.09--0.4) |
| Ghana | 77 (36-136) | 1.06 (0.51-1.85) | 143 (66-290) | 0.72 (0.35-1.44) | -0.95 (-1.8--0.11) |
| Greece | 581 (320-885) | 3.78 (2.08-5.82) | 445 (241-681) | 2.04 (1.11-3.2) | -1.54 (-2.29--0.78) |
| Greenland | 1 (0-2) | 2.29 (0.85-4.43) | 2 (1-3) | 2.86 (1.55-4.44) | 2.94 (2.36-3.52) |
| Grenada | 2 (1-3) | 2.55 (1.5-3.99) | 2 (1-4) | 1.89 (1.1-3.14) | 0.52 (0.01-1.03) |
| Guam | 0 (0-1) | 0.49 (0.28-0.75) | 0 (0-1) | 0.15 (0.07-0.27) | -2.8 (-3.27--2.33) |
| Guatemala | 68 (32-120) | 1.74 (0.85-3.02) | 65 (31-112) | 0.57 (0.27-0.97) | -3.23 (-4.04--2.42) |
| Guinea | 24 (12-43) | 0.69 (0.33-1.21) | 67 (31-119) | 1.09 (0.51-1.95) | 1.59 (0.69-2.5) |

**Continued Supplementary Table 8.** Numbers, ASR, and Trends in Occupational Carcinogen-Attributable LC DALYs across countries and territories 1990–2021.

| Characteristics | 1990 | | 2021 | | 1990–2021 |
| --- | --- | --- | --- | --- | --- |
|  | Number of DALYs cases (95% UI) | The age-standardized DALYs rate/100000 (95% UI) | Number of DALYs cases (95% UI) | The age-standardized DALYs rate/100000 (95% UI) | Number of DALYs cases (95% UI) |
| Guinea-Bissau | 7 (3-13) | 1.59 (0.8-2.98) | 14 (7-27) | 1.57 (0.74-3.1) | 0.22 (-0.97-1.42) |
| Guyana | 7 (4-12) | 1.76 (0.97-2.91) | 12 (6-22) | 1.69 (0.85-2.97) | 1.57 (0.95-2.19) |
| Haiti | 136 (66-233) | 3.94 (1.97-6.63) | 307 (136-575) | 3.96 (1.75-7.39) | 0.6 (-0.24-1.44) |
| Honduras | 29 (15-51) | 1.33 (0.7-2.29) | 143 (73-243) | 2.16 (1.11-3.59) | 2.41 (1.53-3.29) |
| Hungary | 744 (336-1336) | 5.31 (2.31-9.67) | 602 (278-1104) | 3.59 (1.6-6.72) | -1.2 (-1.79--0.61) |
| Iceland | 6 (3-8) | 1.96 (1.07-2.93) | 6 (3-9) | 1.01 (0.59-1.52) | -1.53 (-1.96--1.09) |
| India | 20724 (9978-36092) | 3.72 (1.85-6.38) | 37436 (19376-64544) | 2.9 (1.52-4.92) | -0.42 (-0.85-0.01) |
| Indonesia | 1404 (615-2514) | 1.19 (0.53-2.13) | 3377 (1479-6339) | 1.19 (0.54-2.2) | 0.75 (0.34-1.16) |
| Iran (Islamic Republic of) | 611 (262-1122) | 1.91 (0.84-3.48) | 1080 (477-1960) | 1.21 (0.54-2.19) | -0.24 (-0.76-0.29) |
| Iraq | 235 (123-412) | 2.87 (1.51-4.96) | 558 (278-967) | 2.18 (1.11-3.65) | -0.79 (-1.74-0.16) |
| Ireland | 186 (107-275) | 4.45 (2.58-6.73) | 104 (59-152) | 1.3 (0.74-1.95) | -3.3 (-3.68--2.92) |
| Israel | 85 (46-132) | 1.81 (0.97-2.82) | 127 (69-202) | 1.07 (0.58-1.73) | -1.75 (-1.95--1.55) |
| Italy | 9579 (5782-13389) | 10.63 (6.39-14.96) | 4895 (3003-6821) | 3.32 (2.02-4.59) | -3.02 (-3.92--2.11) |
| Jamaica | 30 (17-48) | 1.75 (0.97-2.88) | 52 (26-93) | 1.7 (0.84-3.01) | 0.23 (-0.25-0.72) |
| Japan | 1594 (866-2424) | 0.93 (0.51-1.4) | 2184 (1254-3130) | 0.54 (0.31-0.78) | -0.18 (-1.25-0.9) |
| Jordan | 28 (14-47) | 1.99 (1.07-3.21) | 56 (28-97) | 0.71 (0.36-1.2) | -3.54 (-4.62--2.45) |
| Kazakhstan | 851 (472-1383) | 5.95 (3.31-9.55) | 380 (208-622) | 1.86 (1.03-3.05) | -4.56 (-4.9--4.22) |
| Kenya | 102 (43-195) | 1.08 (0.45-2.02) | 483 (207-884) | 1.74 (0.77-3.17) | 2.18 (1.07-3.3) |

**Continued Supplementary Table 8.** Numbers, ASR, and Trends in Occupational Carcinogen-Attributable LC DALYs across countries and territories 1990–2021.

| Characteristics | 1990 | | 2021 | | 1990–2021 |
| --- | --- | --- | --- | --- | --- |
|  | Number of DALYs cases (95% UI) | The age-standardized DALYs rate/100000 (95% UI) | Number of DALYs cases (95% UI) | The age-standardized DALYs rate/100000 (95% UI) | Number of DALYs cases (95% UI) |
| Kiribati | 0 (0-0) | 0.05 (0.02-0.1) | 0 (0-0) | 0.11 (0.04-0.22) | 3.7 (3.03-4.37) |
| Kuwait | 10 (5-18) | 1.31 (0.69-2.23) | 13 (7-22) | 0.42 (0.24-0.68) | -1.67 (-2.58--0.75) |
| Kyrgyzstan | 90 (43-162) | 2.75 (1.31-4.92) | 56 (26-100) | 0.97 (0.46-1.72) | -3.1 (-3.56--2.63) |
| Lao People's Democratic Republic | 49 (20-95) | 2.08 (0.85-3.95) | 72 (30-137) | 1.34 (0.6-2.55) | -1.19 (-1.93--0.44) |
| Latvia | 127 (62-226) | 3.56 (1.72-6.36) | 60 (30-100) | 1.79 (0.89-3.13) | -2.23 (-2.84--1.61) |
| Lebanon | 125 (66-209) | 5.41 (2.89-8.75) | 183 (101-282) | 3.11 (1.72-4.86) | -0.96 (-1.25--0.67) |
| Lesotho | 35 (19-59) | 4.03 (2.18-6.72) | 98 (43-178) | 8.53 (3.8-15.41) | 2.99 (2.19-3.8) |
| Liberia | 11 (5-21) | 0.91 (0.41-1.71) | 25 (10-51) | 1 (0.42-1.95) | -0.03 (-1.09-1.03) |
| Libya | 57 (23-113) | 2.63 (1.08-5.17) | 125 (49-243) | 1.89 (0.77-3.62) | -0.2 (-0.93-0.53) |
| Lithuania | 186 (90-328) | 4.11 (1.98-7.28) | 102 (48-169) | 2.05 (0.95-3.52) | -2.27 (-2.84--1.69) |
| Luxembourg | 39 (22-56) | 7.12 (4.04-10.15) | 26 (15-38) | 2.43 (1.41-3.6) | -3.08 (-3.63--2.53) |
| Madagascar | 113 (45-210) | 1.9 (0.76-3.51) | 216 (95-411) | 1.42 (0.64-2.66) | -0.89 (-1.88-0.1) |
| Malawi | 30 (12-57) | 0.66 (0.28-1.26) | 74 (32-150) | 0.82 (0.36-1.63) | 0.4 (-0.7-1.52) |
| Malaysia | 180 (83-323) | 1.74 (0.82-3.12) | 429 (207-749) | 1.41 (0.69-2.44) | -0.1 (-0.64-0.45) |
| Maldives | 1 (1-2) | 1.32 (0.67-2.23) | 2 (1-3) | 0.49 (0.26-0.87) | -2.86 (-3.75--1.96) |
| Mali | 46 (23-81) | 1 (0.52-1.72) | 91 (45-171) | 0.88 (0.44-1.63) | -0.35 (-1.36-0.68) |
| Malta | 23 (13-34) | 5.36 (3.04-7.93) | 21 (12-30) | 2.16 (1.23-3.26) | -1.73 (-2.41--1.05) |
| Marshall Islands | 0 (0-0) | 0.64 (0.29-1.17) | 0 (0-0) | 0.55 (0.22-1.05) | 0.67 (-0.29-1.64) |
| Mauritania | 8 (4-13) | 0.73 (0.37-1.29) | 16 (7-32) | 0.7 (0.32-1.34) | 0.05 (-0.89-0.99) |

**Continued Supplementary Table 8.** Numbers, ASR, and Trends in Occupational Carcinogen-Attributable LC DALYs across countries and territories 1990–2021.

| Characteristics | 1990 | | 2021 | | 1990–2021 |
| --- | --- | --- | --- | --- | --- |
|  | Number of DALYs cases (95% UI) | The age-standardized DALYs rate/100000 (95% UI) | Number of DALYs cases (95% UI) | The age-standardized DALYs rate/100000 (95% UI) | Number of DALYs cases (95% UI) |
| Mauritius | 19 (10-33) | 2.39 (1.22-4.13) | 28 (13-50) | 1.46 (0.69-2.56) | 0.13 (-0.47-0.74) |
| Mexico | 1232 (719-1956) | 2.82 (1.67-4.4) | 1587 (861-2528) | 1.22 (0.67-1.93) | -1.91 (-2.47--1.35) |
| Micronesia (Federated States of) | 0 (0-1) | 0.72 (0.36-1.28) | 1 (0-1) | 0.6 (0.27-1.12) | 0.29 (-0.41-0.99) |
| Monaco | 12 (6-20) | 17.42 (8.57-29.75) | 9 (5-14) | 9.24 (4.96-15.08) | -1.93 (-3.02--0.82) |
| Mongolia | 20 (10-38) | 1.78 (0.85-3.29) | 29 (14-57) | 0.98 (0.49-1.88) | -1.34 (-2--0.67) |
| Montenegro | 29 (12-54) | 4.39 (1.81-7.98) | 42 (19-77) | 4.26 (1.89-7.8) | 0.44 (-0.02-0.89) |
| Morocco | 279 (113-522) | 1.81 (0.75-3.36) | 544 (216-1009) | 1.42 (0.57-2.62) | 0.15 (-0.3-0.61) |
| Mozambique | 129 (50-237) | 1.8 (0.73-3.28) | 335 (146-629) | 2.44 (1.11-4.56) | 1.2 (0.16-2.26) |
| Myanmar | 288 (118-561) | 1.11 (0.46-2.11) | 508 (249-982) | 0.94 (0.47-1.79) | 0.07 (-0.35-0.49) |
| Namibia | 39 (21-63) | 5.76 (3.17-9.18) | 65 (33-108) | 4.65 (2.28-7.74) | -1.12 (-2.12--0.11) |
| Nauru | 0 (0-0) | 0.81 (0.4-1.43) | 0 (0-0) | 0.61 (0.27-1.14) | -0.79 (-1.61-0.04) |
| Nepal | 403 (155-749) | 3.6 (1.42-6.6) | 542 (247-1035) | 2.17 (1.02-4.1) | -0.92 (-1.51--0.32) |
| Netherlands | 1185 (714-1649) | 5.97 (3.58-8.37) | 863 (528-1228) | 2.35 (1.42-3.31) | -2.62 (-3.27--1.95) |
| New Zealand | 134 (80-186) | 3.33 (1.97-4.6) | 110 (65-158) | 1.25 (0.74-1.78) | -2.6 (-3.1--2.09) |
| Nicaragua | 15 (6-27) | 0.89 (0.39-1.6) | 46 (20-86) | 0.88 (0.38-1.63) | 1.12 (0.28-1.96) |
| Niger | 31 (14-55) | 0.94 (0.46-1.67) | 82 (35-160) | 0.86 (0.38-1.67) | -0.16 (-1.43-1.11) |
| Nigeria | 670 (318-1276) | 1.37 (0.66-2.56) | 940 (444-1731) | 0.87 (0.42-1.56) | -1.9 (-2.9--0.89) |
| Niue | 0 (0-0) | 0.48 (0.23-0.87) | 0 (0-0) | 0.37 (0.17-0.65) | -0.68 (-1.02--0.34) |
| North Macedonia | 54 (19-113) | 2.64 (0.94-5.43) | 81 (28-167) | 2.34 (0.81-4.81) | 0.22 (-0.19-0.62) |

**Continued Supplementary Table 8.** Numbers, ASR, and Trends in Occupational Carcinogen-Attributable LC DALYs across countries and territories 1990–2021.

| Characteristics | 1990 | | 2021 | | 1990–2021 |
| --- | --- | --- | --- | --- | --- |
|  | Number of DALYs cases (95% UI) | The age-standardized DALYs rate/100000 (95% UI) | Number of DALYs cases (95% UI) | The age-standardized DALYs rate/100000 (95% UI) | Number of DALYs cases (95% UI) |
| Northern Mariana Islands | 0 (0-0) | 0.7 (0.32-1.29) | 0 (0-0) | 0.44 (0.22-0.76) | -0.8 (-1.41--0.18) |
| Norway | 150 (92-206) | 2.19 (1.33-3.02) | 101 (60-145) | 0.96 (0.56-1.37) | -2.19 (-2.88--1.5) |
| Oman | 4 (2-7) | 0.57 (0.28-0.98) | 15 (8-26) | 0.7 (0.37-1.21) | 2.46 (1.16-3.78) |
| Pakistan | 2867 (1463-5001) | 4.67 (2.42-8.08) | 8114 (3888-14496) | 5.59 (2.78-9.94) | 0.37 (-0.44-1.2) |
| Palau | 0 (0-0) | 0.4 (0.18-0.74) | 0 (0-0) | 0.32 (0.15-0.59) | 0.54 (0.22-0.86) |
| Palestine | 8 (4-16) | 0.92 (0.44-1.77) | 16 (7-31) | 0.55 (0.25-1.01) | -1.47 (-2.52--0.42) |
| Panama | 21 (11-37) | 1.4 (0.72-2.42) | 32 (15-53) | 0.72 (0.34-1.21) | -1.55 (-1.96--1.15) |
| Papua New Guinea | 7 (3-13) | 0.3 (0.12-0.62) | 17 (7-33) | 0.29 (0.13-0.53) | -0.09 (-1-0.83) |
| Paraguay | 40 (19-72) | 1.71 (0.83-3.04) | 181 (86-336) | 2.96 (1.43-5.47) | 2.81 (2.25-3.37) |
| Peru | 216 (117-352) | 1.81 (0.99-2.9) | 255 (125-456) | 0.75 (0.38-1.34) | -2.55 (-3.12--1.98) |
| Philippines | 357 (171-636) | 1.02 (0.5-1.81) | 856 (408-1531) | 0.91 (0.44-1.61) | 0.22 (-0.37-0.81) |
| Poland | 2040 (948-3707) | 4.66 (2.14-8.51) | 2979 (1665-4498) | 4.25 (2.36-6.51) | 0.91 (0.36-1.47) |
| Portugal | 596 (281-1059) | 4.44 (2.04-8.01) | 493 (264-790) | 2.29 (1.2-3.78) | -1.34 (-1.98--0.7) |
| Puerto Rico | 78 (42-130) | 2.19 (1.17-3.63) | 48 (24-80) | 0.74 (0.35-1.28) | -2.49 (-3.01--1.96) |
| Qatar | 4 (2-7) | 2.69 (1.38-4.76) | 17 (8-33) | 1.39 (0.68-2.58) | -1.8 (-3.06--0.53) |
| Republic of C么te d'Ivoire | 69 (34-129) | 1.43 (0.73-2.52) | 162 (69-322) | 1.17 (0.52-2.34) | -0.8 (-1.94-0.36) |
| Republic of Korea | 763 (302-1539) | 2.37 (1-4.64) | 502 (226-913) | 0.52 (0.24-0.95) | -4.03 (-4.47--3.6) |
| Republic of Moldova | 130 (54-256) | 2.75 (1.14-5.42) | 79 (35-151) | 1.35 (0.59-2.65) | -1.99 (-2.43--1.54) |

**Continued Supplementary Table 8.** Numbers, ASR, and Trends in Occupational Carcinogen-Attributable LC DALYs across countries and territories 1990–2021.

| Characteristics | 1990 | | 2021 | | 1990–2021 |
| --- | --- | --- | --- | --- | --- |
|  | Number of DALYs cases (95% UI) | The age-standardized DALYs rate/100000 (95% UI) | Number of DALYs cases (95% UI) | The age-standardized DALYs rate/100000 (95% UI) | Number of DALYs cases (95% UI) |
| Romania | 1294 (510-2652) | 4.47 (1.75-9.15) | 1191 (538-2214) | 3.68 (1.64-6.94) | -0.52 (-1.04-0.01) |
| Russian Federation | 8590 (4019-15565) | 4.53 (2.11-8.22) | 4463 (2285-7306) | 1.9 (0.95-3.18) | -3.05 (-3.54--2.55) |
| Rwanda | 98 (41-179) | 2.96 (1.23-5.43) | 102 (41-203) | 1.37 (0.56-2.71) | -3.13 (-4.13--2.11) |
| Saint Kitts and Nevis | 2 (1-3) | 4.92 (2.93-7.27) | 3 (2-5) | 4.39 (2.57-6.67) | 0.26 (-0.11-0.63) |
| Saint Lucia | 3 (1-4) | 3.02 (1.67-4.93) | 6 (3-10) | 2.3 (1.12-3.93) | 0.35 (-0.08-0.78) |
| Saint Vincent and the Grenadines | 2 (1-4) | 3.17 (1.74-5.31) | 5 (3-9) | 3.7 (1.95-6.09) | 1.77 (1.36-2.17) |
| Samoa | 0 (0-0) | 0.24 (0.11-0.41) | 0 (0-1) | 0.17 (0.08-0.32) | -0.95 (-1.57--0.33) |
| San Marino | 2 (1-3) | 5.2 (2.41-8.85) | 2 (1-3) | 2.16 (0.83-4.13) | -1.26 (-2.09--0.43) |
| Sao Tome and Principe | 0 (0-1) | 0.55 (0.27-0.96) | 1 (0-1) | 0.49 (0.24-0.86) | -0.7 (-1.67-0.28) |
| Saudi Arabia | 47 (19-91) | 0.65 (0.26-1.26) | 123 (52-252) | 0.4 (0.17-0.81) | -0.91 (-1.66--0.16) |
| Senegal | 42 (22-72) | 1.19 (0.62-2.05) | 85 (41-152) | 0.99 (0.48-1.79) | -0.29 (-1.25-0.68) |
| Serbia | 520 (223-1040) | 4.05 (1.72-8.12) | 478 (198-920) | 3.14 (1.29-6.19) | -0.74 (-1.34--0.13) |
| Seychelles | 3 (2-5) | 5.63 (2.85-9.36) | 5 (3-10) | 4.16 (1.96-7.21) | 0.65 (0.29-1.01) |
| Sierra Leone | 19 (9-35) | 0.91 (0.44-1.64) | 34 (17-67) | 0.82 (0.41-1.59) | -0.27 (-1.26-0.73) |
| Singapore | 67 (37-103) | 3.26 (1.9-4.9) | 57 (30-86) | 0.67 (0.35-1.03) | -4.56 (-5--4.12) |
| Slovakia | 295 (121-595) | 5.07 (2.07-10.31) | 226 (97-439) | 2.51 (1.06-4.96) | -1.62 (-2.06--1.18) |
| Slovenia | 116 (62-186) | 4.61 (2.44-7.43) | 133 (70-213) | 3.19 (1.66-5.11) | -0.13 (-0.85-0.58) |
| Solomon Islands | 1 (0-1) | 0.44 (0.2-0.87) | 3 (1-6) | 0.69 (0.28-1.33) | 2.68 (1.77-3.61) |

**Continued Supplementary Table 8.** Numbers, ASR, and Trends in Occupational Carcinogen-Attributable LC DALYs across countries and territories 1990–2021.

| Characteristics | 1990 | | 2021 | | 1990–2021 |
| --- | --- | --- | --- | --- | --- |
|  | Number of DALYs cases (95% UI) | The age-standardized DALYs rate/100000 (95% UI) | Number of DALYs cases (95% UI) | The age-standardized DALYs rate/100000 (95% UI) | Number of DALYs cases (95% UI) |
| Somalia | 74 (31-151) | 2.23 (0.95-4.55) | 163 (63-328) | 2 (0.81-3.99) | -0.84 (-1.99-0.31) |
| South Africa | 918 (526-1472) | 4.24 (2.43-6.79) | 1400 (825-2078) | 2.94 (1.74-4.39) | -1.09 (-1.82--0.35) |
| South Sudan | 53 (22-103) | 1.89 (0.8-3.68) | 85 (33-170) | 1.72 (0.68-3.43) | -0.52 (-1.69-0.66) |
| Spain | 4215 (2370-6403) | 7.93 (4.44-12.25) | 2703 (1471-4149) | 2.9 (1.55-4.49) | -2.8 (-3.46--2.13) |
| Sri Lanka | 102 (52-170) | 0.9 (0.47-1.47) | 325 (140-661) | 1.17 (0.51-2.36) | 3.08 (2.65-3.51) |
| Sudan | 197 (77-425) | 1.91 (0.75-4.13) | 332 (139-651) | 1.42 (0.6-2.7) | -0.97 (-1.91--0.03) |
| Suriname | 2 (1-4) | 0.86 (0.4-1.54) | 7 (3-12) | 0.95 (0.45-1.79) | 1.51 (1.17-1.86) |
| Sweden | 210 (125-294) | 1.37 (0.81-1.91) | 164 (98-237) | 0.7 (0.42-1) | -1.41 (-2.3--0.51) |
| Switzerland | 449 (256-658) | 4.55 (2.59-6.69) | 319 (190-466) | 1.78 (1.04-2.6) | -2.05 (-2.75--1.34) |
| Syrian Arab Republic | 87 (34-168) | 1.42 (0.58-2.72) | 126 (50-245) | 0.8 (0.32-1.53) | -1.1 (-2--0.2) |
| Taiwan (Province of China) | 285 (138-483) | 1.61 (0.8-2.71) | 397 (211-655) | 0.96 (0.51-1.59) | -0.71 (-1.13--0.28) |
| Tajikistan | 52 (23-93) | 1.74 (0.78-3.09) | 61 (27-110) | 0.88 (0.41-1.55) | -2.03 (-2.85--1.21) |
| Thailand | 1010 (489-1728) | 2.48 (1.26-4.2) | 1940 (853-3522) | 1.78 (0.78-3.26) | -0.11 (-0.49-0.28) |
| Timor-Leste | 3 (1-6) | 0.92 (0.36-1.79) | 8 (4-15) | 0.9 (0.43-1.72) | 0.4 (-0.42-1.22) |
| Togo | 16 (8-30) | 1.16 (0.58-2.12) | 67 (31-123) | 1.43 (0.67-2.61) | 1.38 (0.29-2.49) |
| Tokelau | 0 (0-0) | 0.44 (0.2-0.87) | 0 (0-0) | 0.31 (0.14-0.58) | -1.02 (-1.16--0.88) |
| Tonga | 0 (0-0) | 0.37 (0.17-0.68) | 0 (0-0) | 0.31 (0.15-0.58) | -0.51 (-1.06-0.04) |
| Trinidad and Tobago | 16 (9-28) | 1.94 (1.08-3.24) | 33 (16-57) | 1.68 (0.82-2.9) | 0.56 (0.23-0.9) |

**Continued Supplementary Table 8.** Numbers, ASR, and Trends in Occupational Carcinogen-Attributable LC DALYs across countries and territories 1990–2021.

| Characteristics | 1990 | | 2021 | | 1990–2021 |
| --- | --- | --- | --- | --- | --- |
|  | Number of DALYs cases (95% UI) | The age-standardized DALYs rate/100000 (95% UI) | Number of DALYs cases (95% UI) | The age-standardized DALYs rate/100000 (95% UI) | Number of DALYs cases (95% UI) |
| Tunisia | 115 (51-231) | 2.08 (0.94-4.12) | 212 (89-395) | 1.48 (0.63-2.75) | -0.17 (-0.5-0.17) |
| Turkey | 3722 (1951-6256) | 10.4 (5.52-17.23) | 3704 (2043-5750) | 3.84 (2.13-5.99) | -2.61 (-3.04--2.18) |
| Turkmenistan | 56 (28-98) | 2.55 (1.29-4.42) | 58 (27-103) | 1.2 (0.57-2.13) | -1.85 (-2.39--1.31) |
| Tuvalu | 0 (0-0) | 0.58 (0.28-1.01) | 0 (0-0) | 0.46 (0.23-0.87) | -0.51 (-0.71--0.3) |
| Uganda | 170 (71-316) | 2.29 (0.95-4.23) | 378 (158-754) | 2.08 (0.88-4.12) | -1.32 (-2.6--0.02) |
| Ukraine | 3167 (1515-5599) | 4.42 (2.11-7.89) | 1489 (677-2719) | 2.06 (0.91-3.83) | -3.14 (-3.71--2.56) |
| United Arab Emirates | 12 (4-24) | 1.54 (0.59-3.14) | 53 (20-105) | 0.65 (0.29-1.21) | -1.8 (-2.9--0.69) |
| United Kingdom | 4656 (2858-6283) | 5.17 (3.17-6.98) | 3660 (2285-4989) | 2.76 (1.71-3.75) | -1.88 (-2.58--1.17) |
| United Republic of Tanzania | 272 (107-519) | 2.17 (0.85-4.1) | 496 (203-988) | 1.62 (0.67-3.23) | -1.24 (-2.22--0.25) |
| United States of America | 9916 (5666-14107) | 3.11 (1.75-4.47) | 9558 (5432-13742) | 1.6 (0.89-2.35) | -2.12 (-2.62--1.62) |
| United States Virgin Islands | 3 (2-5) | 2.93 (1.72-4.95) | 4 (2-7) | 2.25 (1.15-3.95) | 0.85 (0.32-1.37) |
| Uruguay | 298 (169-473) | 7.92 (4.45-12.66) | 214 (128-333) | 4.25 (2.52-6.68) | -1.58 (-1.93--1.22) |
| Uzbekistan | 296 (137-534) | 2.34 (1.09-4.2) | 261 (121-511) | 0.81 (0.39-1.57) | -1.94 (-2.57--1.3) |
| Vanuatu | 0 (0-1) | 0.48 (0.21-0.91) | 1 (0-2) | 0.42 (0.2-0.77) | -0.15 (-0.98-0.7) |
| Venezuela (Bolivarian Republic of) | 319 (162-552) | 3.09 (1.58-5.31) | 731 (336-1319) | 2.29 (1.06-4.12) | -0.55 (-1.08--0.02) |

**Continued Supplementary Table 8.** Numbers, ASR, and Trends in Occupational Carcinogen-Attributable LC DALYs across countries and territories 1990–2021.

| Characteristics | 1990 | | 2021 | | 1990–2021 |
| --- | --- | --- | --- | --- | --- |
|  | Number of DALYs cases (95% UI) | The age-standardized DALYs rate/100000 (95% UI) | Number of DALYs cases (95% UI) | The age-standardized DALYs rate/100000 (95% UI) | Number of DALYs cases (95% UI) |
| Viet Nam | 616 (294-1152) | 1.47 (0.71-2.74) | 2268 (1047-4364) | 2 (0.93-3.84) | 2.48 (2.01-2.95) |
| Yemen | 118 (45-247) | 2 (0.77-4.2) | 259 (94-515) | 1.54 (0.58-3.04) | -0.78 (-1.88-0.33) |
| Zambia | 85 (36-155) | 2.49 (1.06-4.47) | 251 (72-745) | 2.81 (0.81-8.06) | 0.16 (-1.05-1.39) |
| Zimbabwe | 115 (55-202) | 2.51 (1.22-4.41) | 180 (85-317) | 2.12 (1.07-3.68) | -0.17 (-1.21-0.87) |
